# The roles of species’ relatedness and climate of origin in determining optical leaf traits over a large set of taxa growing at high elevation and high latitude

**DOI:** 10.3389/fpls.2022.1058162

**Published:** 2022-12-16

**Authors:** Saara M. Hartikainen, T. Matthew Robson

**Affiliations:** ^1^ Canopy Spectral Ecology and Ecophysiology Group (CanSEE), Organismal and Evolutionary Biology (OEB), Viikki Plant Science Centre (ViPS), Faculty of Biological and Environmental Sciences, University of Helsinki, Helsinki, Finland; ^2^ National School of Forestry, University of Cumbria, Ambleside, United Kingdom

**Keywords:** botanical gardens, flavonoids, leaf traits, mountain species, photoprotection, UV radiation

## Abstract

Climate change is driving many mountain plant species to higher elevations and northern plant species to higher latitudes. However, various biotic or abiotic constraints may restrict any range shift, and one relevant factor for migration to higher elevations could be species’ ability to tolerate high UV-doses. Flavonoids are engaged in photoprotection, but also serve multiple ecological roles. We compared plant optical leaf trait responses of a large set of taxa growing in two botanical gardens (French Alps and southern Finland), considering potential constraints imposed by the relatedness of taxa and the legacy of climatic conditions at plants’ original collection sites. The segregation of optically measured leaf traits along the phylogeny was studied using a published mega-tree GBOTB.extended.tre for vascular plants as a backbone. For a subset of taxa, we investigated the relationship between climatic conditions (namely solar radiation, temperature and precipitation at a coarse scale) at the plants’ original collection site and current trait values. Upon testing the phylogenetic signal (Pagel’s λ), we found a significant difference but intermediate lambda values overall for flavonol or flavone index (I_flav_) and anthocyanin index (I_ant_), indicating that phylogenetic relatedness alone failed to explain the changes in trait values under a Brownian motion model of trait evolution. The local analysis (local indicator of phylogenetic association) indicated mostly positive autocorrelations for I_flav_ i.e. similarities in optically measured leaf traits, often among species from the same genus. We found significant relationships between climatic variables and leaf chlorophyll index (I_chl_), but not I_flav_, particularly for annual solar radiation. Changes in plants’ I_flav_ across microhabitats differing in UV irradiance and predominately high *F*_v_ */F*_m_ indicated that most plants studied had sufficient flexibility in photoprotection, conferred by I_flav_, to acclimate to contemporary UV irradiances in their environment. While not explaining the mechanisms behind observed trait values, our findings do suggest that some high-elevation taxa display similar leaf flavonoid accumulation responses. These may be phylogenetically constrained and hence moderate plants’ capacity to adjust to new combinations of environmental conditions resulting from climate change.

## 1 Introduction

Plants growing in mountain regions are exposed to high solar and UV radiation (Blumthaler, 2012), while low temperatures restrict the period of growth and retard physiological processes (Wielgolaski and Karlsen, 2007; Körner et al., 2019). Plant species have adapted to these conditions by evolving multiple protective mechanisms against excessive sunlight which often function simultaneously (Streb and Cornic, 2012 and references therein; Fernández-Marín et al., 2021). These mechanisms include avoiding excess light energy through leaf structure and position (angle), chloroplast relocation, the dissipation of excess absorbed energy *via* xanthophyll cycle pigments and antioxidant systems which prevent damage to PSII (Ort, 2001; Demmig-Adams and Adams, 2006). Flavonoids are multifunctional secondary metabolites ubiquitous in plants (Grotewold, 2006) that contribute to photoprotection (Agati et al., 2020). This potentially makes the flavonoid responses particularly relevant for plant species from high elevation environments.

In response to climate change, species must adjust to new conditions or migrate to a location where conditions are suited to their ecological requirements. Some mountain species have been reported to shift their distribution to higher elevations (Rumpf et al., 2018; He et al., 2019), but where suitable microhabitats remain the displacement of species may be slower (Scherrer and Körner, 2010 and Scherrer and Körner, 2011). Climate-change-associated range shifts are predicted for many species globally (Chen et al., 2011; Parmesan and Hanley, 2015), including projected changes in high latitude plant species (Wróblewska and Mirski, 2018). Whether *in situ* or during migration, trait adaptation and phenotypic plasticity are the processes that determine how well plants tolerate and acclimate to new conditions (Merilä, 2012; Merilä and Hendry, 2014). The potential limitations imposed by traits involved in photoprotection may constrain migration to higher elevations, which typically exposes plants to larger fluctuations in temperature and sunlight, including higher maximum UV-B irradiance.

The phylogenetic relatedness of species affects the extent of independence in their traits (Harvey and Pagel, 1991), albeit the presence of a phylogenetic signal should not be presumed (Losos, 2008). A phylogenetic signal refers to the “*tendency for related species to resemble each other more than they resemble species drawn at random from the* (phylogenetic) *tree*” (Blomberg and Garland, 2002). However, similar trait values among related taxa may be a result of various evolutionary processes (Losos, 2008; Revell et al., 2008). It is therefore important to study how plant functional traits are distributed across different taxonomic levels (genus, species and infraspecific taxa) to gain knowledge of their inter-relatedness and potential constraints in their responses. Some species or populations have been found to maintain high concentrations of leaf flavonoids or UV-absorbing compounds, appearing relatively insensitive to changes in environmental conditions, including UV-B irradiance (Barnes et al., 1987; Ziska et al., 1992; Nybakken et al., 2004a). In contrast, the vast majority of species studied show variation in leaf flavonoid or phenolic contents according to environmental cues, such as fluctuating solar radiation (Searles et al., 2001; Agati et al., 2009; Barnes et al., 2013) or temperature (Bilger et al., 2007). For instance, *Silene vulgaris* (Moench) Garcke plants growing at high elevations contain more foliar non-anthocyanin flavonoids than those at low elevation (Berardi et al., 2016). Clines in species’ traits such as these are often found along environmental gradients (Woods et al., 2012; de Villemereuil et al., 2018), and in some cases a relationship between leaf UV-absorbing compounds and original elevation has been reported (Ziska et al., 1992; Rozema et al., 1997). For instance, ecotypes of dwarf shrub *Dryas octopetala* L. have been found to differ in their epidermal UV-B transmittance according to latitude and received UV-B radiation (Nybakken et al., 2004a). While elevated UV-B radiation is well known to elicit accumulation of flavonoids and related phenolic compounds in plants (Searles et al., 2001), there is often limited evidence supporting a general positive correlation between leaf flavonoid contents and received solar UV radiation (Nybakken et al., 2004b; Jaakola and Hohtola, 2010; Barnes et al., 2016b). Such a relationship may be conditioned by leaf development (Laitinen et al., 2002) and leaf inclination angle (Grant, 1999), respectively affecting patterns of leaf flavonoids, and the incident UV-B doses received by plants.

Plant functional traits may be used to study species responses to increasing temperatures with climate change (Cornelissen and Makoto, 2014). Furthermore, leaf trait responses to UV-B radiation and their constraints have been assessed for some species under controlled experimental settings (e.g. Watermann et al., 2020). However, it can be difficult to scale up the results from experimental approaches to interpret how differing taxa will respond to changes in their habitats. We assessed optically measured leaf traits, particularly the index estimating leaf adaxial flavonols and/or flavones (I_flav_), from a large set of taxa growing together in a common environment mostly at high-elevation and tried to detect the signals of any potential constraints on trait values connected to phylogenetic relatedness and legacy of climate at plants’ origin. We further investigated whether the studied taxa show flexibility in I_flav_ according to spectral irradiance in their microenvironment at their current growing location, in an effort to distinguish potential drivers of I_flav_. We specifically ask: (1) whether patterns in these optically measured leaf traits, following the phylogenetic relatedness of taxa can be identified from a sample of 622 taxa, and (2) whether climatic conditions at the site of origin of the plants were associated with their I_flav_. If present, these two response patterns could potentially constrain the I_flav_ response. To study these patterns in leaf traits, we made optical measurements from 622-672 plant taxa growing in a high-elevation environment at an alpine botanical garden (Col du Lautaret, France) and from 86 taxa (including 27 of the same species found in the alpine botanical garden) from a high-latitude environment in the Kumpula Botanical Garden (Helsinki, Finland). These two study sites differ in their solar irradiance, but also in air temperatures and seasonality (**Figure S1**).

## 2 Materials and methods

### 2.1 Study sites

The study was conducted in an alpine botanical garden at the Joseph Fourier Field Station (Université Grenoble Alps, France) on the Col du Lautaret in the French Alps over two consecutive summers (19.06.-01.07.2014 and 21.06.-06.07.2015). This alpine botanical garden (2100 m a.s.l.; 45°2’ 9” N, 6°23’ 59” E) holds a collection of mainly high-elevation species and some high-latitude species originally collected from different parts of the world. These plants were grown from seeds acquired either directly from plants in their original habitat, or from other botanical gardens. Prior to planting, seeds germinated at the university facilities in Grenoble and acclimated to conditions in the alpine botanical garden at their seedling nursery. We only measured plants that were well-established growing in the alpine botanical garden. Only few trees were sampled, while most plants were forbs, dwarf shrubs or shrubs, representing typical features of mountain flora (e.g., for high snowbed species: Körner et al., 2019). Weather data were obtained from e-METSYS/JFAS weather station (Vantage Pro 2 Plus, Davis Instruments, Hayward, CA, USA) located at the study site. Daily maximum photosynthetically active radiation (PAR: 400-700 nm) over the sampling period in both years is presented in **Figure S2**.

To provide a counterpoint to conditions at the alpine botanical garden and investigate potential variation in optically measured leaf traits, plants were also sampled from 86 taxa growing at high latitude in the Kumpula Botanical Garden, Helsinki (14 m a.s.l.; 60° 12’ 7” N, 24° 57’ 26” E; LUOMUS, University of Helsinki, Finland) during June of 2015. The Kumpula Botanical Garden contains plant taxa from Europe, eastern North America, western North America, the continental Far East, and Japan (LUOMUS, 2021). Sampled taxa represented the same genera and families, or in 27 cases the same species, as those sampled in France. Most sampled plant taxa from Kumpula Botanical Garden were native to France and little over half were part of the native Finnish flora, while 40% were native to both areas. Temperature and solar radiation data were obtained from a weather station located in Kumpula maintained by the Finnish Meteorological Institute. Daily solar radiation (global, direct and diffuse) for the sampling period in 2015 are presented in **Figure S3**. Monthly fluctuations in air temperature, solar radiation and UV-B radiation at both sites are given in **Figure S1**.

Collection locations of the plant taxa growing in Kumpula were extracted from the database of Finnish Museum of Natural History (LUOMUS). The locations of the original seed collection sites of the taxa in the alpine botanical garden were obtained from the records of the alpine botanical garden courtesy of Rolland Douzet. The approximate coordinates were obtained based on this information using maximum, typically southern, slope exposure. The same protocol was used for those taxa from Kumpula Botanical Garden missing origin coordinates, but with elevation and specific location information. Because of the scope for small-scale micro-environmental effects, these coordinates present only rough approximations of the plants’ environmental conditions.

### 2.2 Solar radiation and weather data for study and original collection locations

In addition to data obtained from weather stations on site, climate data for the botanical gardens and the plants’ original collection sites were obtained for variables reflecting their temperature, precipitation (annual mean temperature/precipitation and their seasonality, min/max temperature of the coldest and warmest months) and solar radiation (annual mean solar radiation, its seasonality, highest and lowest weekly solar radiation, solar radiation of the wettest/driest/warmest/coldest quarters in W m^-2^) from WorldClim (Bio 1-15, Fick and Hijmans, 2017) and CliMond (Bio 20-27, Hutchinson et al., 2009; Kriticos et al., 2014) databases based on observations for years 1970-2000 and 1961-1990 respectively. Additionally, mean monthly solar radiation (kJ m^-2^ d ^-1^) was acquired from WorldClim (Fick and Hijmans, 2017). The resolution of the data is 30 arc seconds for Bio 1-15 and 10 arc minutes for Bio 20-27. A global UV-B radiation climatology based on remotely sensed records from NASAs Ozone Monitoring Instrument (Aura-OMI) over 2004-2013 (Beckmann et al., 2014) was used to compare UV-B radiation at study sites and at the 58 original collection locations (resolution of 15 arc minutes).

To account for micro-environmental variation in solar irradiance throughout the alpine botanical garden (29 locations), we measured solar spectral irradiance from the UV-B to near-infrared (NIR) regions (290-900 nm) with a CCD array spectroradiometer Maya 2000 Pro (Ocean Optics, Dunedin, FL, USA) with D7-H-SMA cosine diffuser (Bentham Instruments Ltd., Reading, UK) (**Supplementary A2**).

### 2.3 Optical measurement of leaf traits, chlorophyll fluorescence and estimation of quenching parameters

Indices of relative absorbance by leaf adaxial side flavonols or flavones i.e. I_flav_ (suggested major absorbers: flavonols in dicotyledons and flavones in monocotyledons, Cerovic et al., 2002 and Cerovic et al., 2012) and anthocyanins (I_ant_), and leaf chlorophyll content (I_chl_) were measured non-invasively with an optical leaf-clip Dualex Scientific ^+^ (Force-A, Paris-Orsay, FR; henceforth Dualex). The formula for each index is given in **Table S1**. Very low values of I_chl_ < 7 AU were eliminated as potential errors. In order to compare leaves under equivalent light environments when UV irradiance is expected to be maximal, measurements were centred around solar noon (no more than ± 3 hours) to exclude significant diurnal changes in UV absorbance by flavonoids and chloroplast movement (Williams et al., 2003; Barnes et al., 2016a). The first distal adult leaf of the main stem was sampled: usually this was the 3^rd^ or 4^th^ leaf from the top which was not shaded by other leaves. In addition to optical measurements, locations within the study sites were categorised according to solar radiation exposure on a scale of 1 (mostly shaded during the day by other plants) to 4 (exposed to full sunlight throughout the day). Plants growing in the alpine botanical garden were visually categorised for their phenology (scale of 1-10, e.g. not fully opened leaves = 1, flower buds about to open = 5, senescent = 10), and approximate leaf inclination angle; the angle between the horizon and leaf blade (scale of 1-9, e.g. for erect = 1, horizontal = 5, and downward leaves = 9).

In 2014, all species growing in the alpine botanical garden were assessed apart from those with leaves too small or thick to be measured with a Dualex. In total, plants from 672 taxa, including some plants at differing developmental stages, were measured (10 172 measurements). These taxa mainly included 572 species plus 42 taxa identified only to the genus (**Table S2**), covering 229 different genera overall. Some of the nomenclature in botanical garden was outdated or lacking, and during sampling period not all measured taxa could be fully identified, Thus, some taxa were identified after this period from photographs, but these were not always sufficient for species-level identification. These plants were identified only to genus-level. Some analyses allowed for the distinction of different developmental stages (e.g. seedlings) in those species where such an approach was deemed necessary (**Table S2**). Sets of leaf optical measurements were made from individual plants, or ramets originating from vegetative growth which were otherwise indistinguishable (≥ 4 individuals per species). For most large tree and shrub species (e.g. *Salix*), the individual count was below this, but we included these species in the analysis to provide coverage of a wide variety of life-forms. Hence, the results for shrub and tree species with fewer than four individuals should be considered indicative. Based on these data, the following year (2015) a set of 86 taxa, representing differing strategies in leaf flavonol/flavone, anthocyanin and chlorophyll content, were selected to test the relationship between I_flav_ and chlorophyll fluorescence. Measurements were made with a mini-PAM fluorometer (Heinz-Walz GmbH, Effeltrich, Germany) from an equivalent set of leaves to those sampled with the Dualex from same group of plants (428 plants in total). Maximum quantum efficiency of PSII photochemistry (henceforth: *F*_v_ */F*_m_) was measured from leaves predawn (1-3 h before sunrise) in the dark, and at midday (approximately ± 3 h from solar noon) after 30 min dark adaptation. Operating efficiency of PSII photochemistry (henceforth: ФPSII) was measured from intact sun-adapted leaves at midday (± 1 h around solar noon) and mid-morning (between 2-4 h after daybreak). The diurnal measurements of ФPSII were made to coincide with peak solar irradiance at midday, but to control for potential midday depression in photosynthetic capacity and ФPSII (e.g. Guo et al., 2009; Koyama & Takemoto, 2014) we repeated these measurements at mid-morning. Non-photochemical quenching (NPQ) was calculated as (*F*_m_ *-F*_m_ *’)/F*_m_ *’* (Murchie and Lawson, 2013), using both predawn and midday *F*_m_ values. Related quenching parameters Y(NO) and Y(NPQ) were calculated according to Klughammer and Schreiber (2008) for both mid-morning and midday measurements. These parameters describe the fraction of energy either passively dissipated in form of heat and fluorescence, Y(NO), or dissipated in form of heat through the regulated photoprotective NPQ mechanism, Y(NPQ) (Klughammer and Schreiber, 2008). Since repeated fluorescence measurements were not exclusively made from the same leaves, we mainly used species’ means for NPQ, Y(NO), Y(NPQ) to account for variability among same group of plants. To assess the relationship between I_flav_ and absorbance of leaf extracts measured with a spectrophotometer, we sampled plants of 49 taxa from the alpine botanical garden in 2015. All related details may be found in **Supplementary A3**. The same protocol described above was used for optically measured leaf traits from the set of plants growing in Kumpula Botanical Garden.

### 2.4 Data analyses

A multivariate approach was used to distinguish groups of taxa with similar optically measured leaf traits from 672 plant taxa growing in the alpine botanical garden. This approach entailed creating hexagonal Kohonen self-organising maps (SOM) with 25 nodes (Kohonen, 1982; R package kohonen by Wehrens and Kruisselbrink, 2018). The number of nodes were selected based on retaining a minimum number of observations (taxa) per node i.e. no empty nodes. This technique provides a simple low-dimensional visualisation of patterns found in high-dimensional data using unsupervised learning (Kohonen, 1982; Wehrens and Kruisselbrink, 2018). Similarly, Kohonen SOMs were also used to segregate optically measured leaf traits from a smaller subset of taxa re-sampled in 2015 in the alpine botanical garden.

To investigate whether patterns in optically measured leaf trait values among the 622 taxa (i.e. leaving out genus -level measurements, hybrids and seedlings) from the alpine botanical garden followed the relatedness of the taxa, we first used V.PhyloMaker (Jin and Qian, 2019), a tool which generates phylogenies using a mega-tree as a backbone (GBOTB.extended.tre, Jin and Qian, 2019). This mega-tree is a combination of previously published phylogenies: GBOTB for the seed plants by Smith and Brown (2018), and the pteridophytes clade from Zanne et al. (2014) phylogeny, with some updates and corrections (further details in Jin and Qian, 2019, also see Qian and Jin, 2016). We used nomenclature following the Plant List database (Plantlist.org), but if originally marked species were unresolved without a clear indication of their accepted nomenclature, the original name was used and species was typically added as a basal polytomy within its family or genus (scenario 1 in Jin and Qian, 2019). Infraspecific taxa (i.e. subspecies and variety) were included in our phylogeny by combining them with their parental species as suggested in Qian and Jin (2016). We then tested for a phylogenetic signal among leaf traits by using Pagel’s lambda (λ) test, which relies on a Brownian motion model of trait evolution (Pagel, 1999), using phytools R package (Revell, 2012 and Revell, 2013) (transformation illustrated in **Figure S4**). These results were further compared with an autocorrelation-based method, Moran’s *I*, and with Blomberg’s *K*, both calculated according to methods in R package phylosignal (Keck et al., 2016; Moran’s *I*: Gittleman and Kot, 1990; Blomberg’s *K*: Blomberg et al., 2003). Finally, to identify local trait patterns, and particular taxa of interest expressing these patterns, we used local indicator of phylogenetic association i.e. local Moran’s *I* (Anselin, 1995) calculated with R function lipaMoran (R package phylosignal; Keck et al., 2016). We repeated above-mentioned phylogenetic analyses separately for leaf traits measured from 86 taxa growing in Kumpula Botanical Garden.

To explore the relationship between climatic conditions at the original collection site of the plants (mostly data available from plant taxa sampled in Finland) and their mean I_flav_ from each location we used Spearman’s rank correlation (*n* = 58, including 62 taxa). For 49 locations trait value was from one taxon, while elsewhere two or more taxa were present, whereby a mean trait value was calculated. The differences between predawn and midday *F*_v_ */F*_m_ for each species, and I_flav_ between the two consecutive years were assessed using Student’s *t*-test or Welch *t*-test for homo- or heterogenous variances respectively. A non-parametric Wilcoxon rank sum test was used for non-normally distributed data and *p*- values were adjusted using Benjamini and Hochberg (1995) correction method. All data exploration and analyses were performed in R version 3.6.1 (2019, The R Foundation for Statistical Computing, Vienna, Austria). All figures except SOM and phylogenetic trees, were created with R package ggplot2 (Wickham, 2016).

## 3 Results

### 3.1 General patterns among taxa in optically measured leaf traits and chlorophyll fluorescence

**Figure 1:**
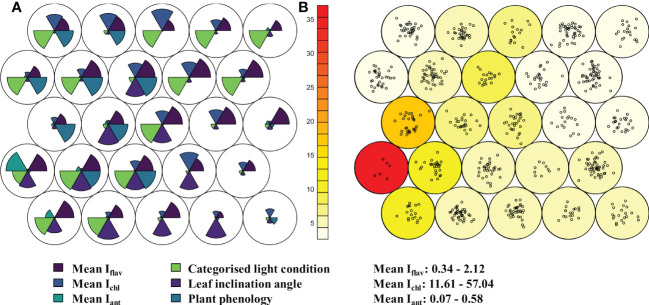
Relative differences in leaf traits from 672 taxa in the alpine botanical garden (Col du Lautaret, France) shown in a hexagonally arranged Kohonen self-organising map (SOM) with 25 nodes. Plants were sampled for optically measured leaf traits; indices of adaxial flavonol/flavone (I_flav_, dark blue, above right wedge) and anthocyanin (I_ant_, turquoise, above left wedge), and leaf chlorophyll (I_chl_, blue, above middle wedge) during the summer of 2014. Panel **(A)** shows the relative differences (radius of each wedge) in three scaled mean leaf traits in the upper half circle. The lower half circle contains relative differences in categorised light condition of the plants, categorised phenological advancement, and categorised leaf inclination angle. Panel **(B)** shows loadings of the nodes i.e., number of taxa grouped in the node as points, and neighbour distance (sum of distances to all immediate neighbours) as colour scale with red for the greatest distance. These analyses allowed for distinction of different developmental stages of those species where such an approach was deemed necessary. Range in mean optically measured leaf traits among taxa are given lower right.

Based on optically measured mean leaf traits of the 672 taxa or developmental stages sampled in the alpine botanical garden, taxa were classified into nodes representing similar trait values using unsupervised Kohonen self-organising map (SOM). The six nodes with highest loadings (covering 249 taxa) were characterised mostly by relatively high I_flav_ and exposed light condition, while the other leaf trait values were mixed in these nodes (**Figure 1**). Only a few nodes were characterised by relatively low I_flav_ (**Figure 1**), and overall only two taxa out of 672 taxa had mean I_flav_ below 0.5 AU (*Adoxa moschatellina* L., *Maianthemum*: 0.3% of taxa), and 25 taxa (3.7% of taxa) had a mean I_flav_ below 1.0 AU (**Table S3**). While I_flav_ was high among many of the plants sampled (**Figure S5**), mean I_ant_ was rarely relatively high, and relatively high mean I_chl_ was not found in most plants (**Figure 1**). Although shaded light conditions did not consistently coincide with relatively low I_flav_ in SOM, nodes with the lowest I_flav_ tended to contain plants from more-shaded conditions (**Figure 1**). In fact, plants experiencing prolonged shading over the day had lower I_flav_ values compared to the overall mean I_flav_ (*p* ≤ 0.015, **Figure S6**), but the I_flav_ of plants experiencing only light or minor shading over the day did not differ from the I_flav_ of those taxa in full sun (Wilcoxon rank sum test: adjusted *p* -value ≥ 0.1). Although neither plant phenology nor approximate leaf inclination angle produced any general distinguishable patterns of segregation by leaf traits in the SOM (**Figure 1**), leaves especially with below horizontal angles, and those few newly produced leaves measured, had a higher I_flav_ than the overall mean (*p* ≤ 0.048, **Figures S7**, **S8**). The range (max - min) of I_flav_ by taxon had a significant but very weak negative relationship with mean I_flav,_ although this relationship was stronger for plants from Kumpula Botanical Garden than from the alpine botanical garden (French Alps: *r* = -0.16, *p* < 0.0001, Kumpula: *r* = -0.43, *p* < 0.0001) and slightly stronger in unshaded plants (**Figure S9**).

Patterns of optically measured leaf traits from taxa growing in Kumpula Botanical Garden roughly resembled those of plants growing in the alpine botanical garden, in that their mean I_flav_ was generally high, and likewise only a small minority of taxa (only *Oxalis acetosella* L. from Kumpula Botanical Garden) had a mean I_flav_ < 0.5 AU (**Table S3**). However, lower I_flav_ values were generally more common from Kumpula Botanical Garden (< 1.0 AU, 16.8%) than among plants growing in the alpine botanical garden (< 1.0 AU, 5.9%) (**Figure S5**; **Table S3**). As in the alpine botanical garden, plants growing in the most shaded environments over the whole day in Kumpula had a lower I_flav_ than the mean I_flav_ from all plants (*p* < 0.0001, **Figure S10**). Among plants with low (< 1.0 AU) mean I_flav_, only one taxon, the genus *Rubus* L., was common to both botanical gardens, whereas among those taxa with the highest mean I_flav_ (> 1.8 AU), several genera were common to both sites (*Geranium*, *Primula*, *Rhododendron* and *Vaccinium*) (**Table S3**). However, these genera had diverse patterns of I_flav_, whereby an individual genus often included some species among those with the highest and the lowest mean I_flav_ (**Table S3**). We did not discern any strict patterns related to life history, leaf longevity, leaf form, or whether taxa were mono- or dicotyledons, that segregated among plants with high or low I_flav_, although these relationships were not explicitly tested.

Finally, there were no significant differences between predawn and midday *F*_v_ */F*_m_ values from most of the taxa we sampled (67 from 86 taxa, 77.01%, **Table S4**). However, in 36 taxa one or more predawn *F*_v_ */F*_m_ value was below 0.78 i.e. a drop of ≥ 0.05 (data not shown) beneath our optimal *F*_v_ */F*_m_ value of 0.83 (Maxwell and Johnson, 2000). In all taxa where significant differences between predawn and midday *F*_v_ */F*_m_ were found (19 taxa, 22.99%), mean values for predawn *F*_v_ */F*_m_ were higher than midday values (**Table S4**). The mean NPQ for taxa sampled from the alpine botanical garden was generally high (**Table S4**). Mean (± SE) NPQ calculated at mid-morning was 4.30 ± 0.13, and at midday it was 3.76 ± 0.12 for the 86 taxa assessed (**Table S4**).

We used a Kohonen SOM based on those taxa growing in the alpine botanical garden, to explore the taxon -level relationships between photochemical quenching parameters and optically measured leaf traits. Nodes with a relatively low predawn *F*_v_ */F*_m_ were also characterised by relatively high Y(NO) (describing the fraction of energy passively dissipated in form of heat and fluorescence) and relatively low NPQ (**Figure S11**), while only one of these taxa, *Lilium martagon* L., also had a notably high mean I_flav_ (1.93 AU). Considering the relative trait values among taxa, those taxa with the highest NPQ also had the highest mean I_ant_ (e.g. *Vaccinium*, *Salix* and *Penstemon* species; 0.22-0.49 AU). The node with most taxa (35) was characterised by relatively high I_flav_ and relatively high *F*_v_ */F*_m_ (**Figure S11**).

### 3.2 Variation in I_flav_ according to the local light environment and interannual comparison

We found a significant overall positive correlation between the mean I_flav_ of plants and their local unweighted UV irradiance (*r* = 0.45-0.47, *p* ≤ 0.046), likewise when expressed as biologically effective UV doses calculated according to various biological spectral weighting functions (*r* = 0.44-0.52, *p* ≤ 0.047) (**Supplementary A2**). On the other hand, the relationship between spectral irradiance and mean I_flav_ was not significant for spectral regions within the PAR nor for calculated spectral photon ratios (**Supplementary A2**).

The differences in I_flav_ between two consecutive summers among plants growing in the alpine botanical garden were significant for the total of 86 taxa sampled in both years (**Figure S12**, right panel). However, the Spearman’s rank correlation for I_flav_ from these same taxa between consecutive summers was significant with *r* = 0.58 and *p* < 0.0001 (**Figure S12**, left panel), indicating consistencies among I_flav_ of taxa between these two years.

### 3.3 Variation in optically measured leaf traits with the environmental conditions at plants’ original collection sites

We did not find significant relationships between the climatic conditions at plant origin and their current I_flav_ after correcting for multiple testing (**Table S5**). Among the optically measured leaf traits, I_chl,_ had the strongest relationship with variables associated with solar radiation (Bio 20, Bio 26, *r* ≥ 0.55, *p* ≤ 0.0007), UV-B radiation (annual mean, seasonality, during highest month and highest quarter, mean during April, August and September, *r* > 0.49, *p* ≤ 0.0018) and elevation (*r* = 0.52, *p* = 0.00103) at the original collection site (**Table S5**). Weaker relationship between I_chl_ and latitude (*r* = -0.38, *p* = 0.019) was also recorded, and contrary to I_chl_, I_ant_ had a negative relationship with elevation (*r* = -0.37, *p* = 0.023) (**Table S5**). Because the correlation of I_chl_ was negative with latitude and was positive with elevation of the original collection site, I_chl_ was also significantly correlated with many of those climatic variables that change concurrently along these geographical gradients (**Table S5**). Similar results were obtained when analyses were rerun using minimum, usually northern, slope exposures for those species with approximated location (data not shown). There was also no confounding evidence that current light conditions where the plants were growing were related to the climatic conditions of origin (**Table S5**).

### 3.4 Comparing variation in optically measured leaf traits and relatedness of taxa

**Figure 2:**
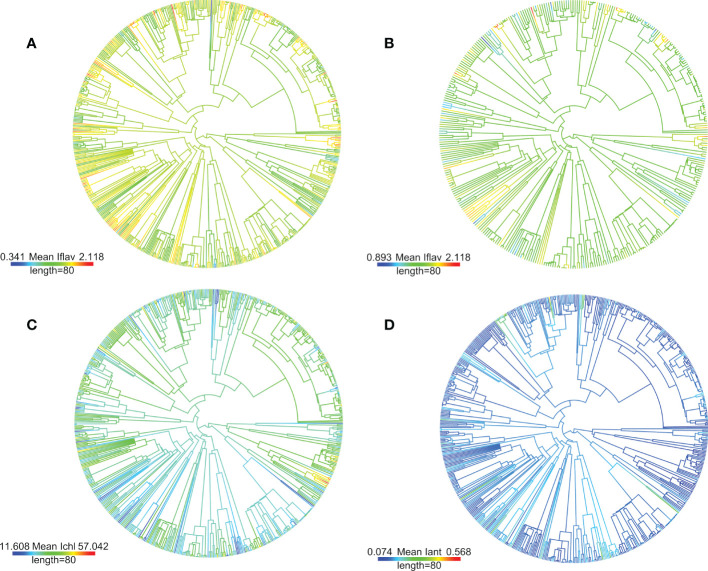
Used phylogeny for the studied 622 taxa from the alpine botanical garden (Col du Lautaret, French Alps) based on previously published mega-tree (GBOTB.extended.tre) and methodology (scenario 1) from Jin and Qian (2019), is plotted with mean leaf trait values (I_flav_ =**A** & **B**, I_chl_ = **C** and I_ant_ = **D**) as colour scale by using R package phytools (Revell, 2012 and Revell, 2013). Colour bar length is a scale for branch lengths (million years, Myr). The plotting method estimates ancestral trait values but estimate of uncertainty is not shown, and ancestral values are mainly shown for visual clarity. The **(B)** panel shows mean I_flav_ of taxa with no plants from shaded microhabitats (categorised light condition maximum of minor shade over day) with differing scale of values. Infraspecific taxa (i.e. subspecies and variety) were included in the phylogeny by combining them with their parental species. High-quality pdf -versions with tip labels may be found at the end of the **Supplementary A1**.

Pagel’s λ test estimating the phylogenetic signal, namely whether related taxa would have more similar leaf trait values, found that the fitted λ value was intermediate (0 < λ < 1), but the likelihood-ratio test against the λ = 0 transformation gave significant differences for all leaf traits measured from plants growing in the alpine botanical garden (**Table 1**). The fitted λ value was slightly higher for I_flav_ and I_ant_ compared to I_chl_ (**Table 1**). It was also slightly reduced for I_flav_ and I_ant_ when only unshaded taxa were included (**Table 1**; **Figure 2** and **Figure S6**). On the contrary, for the smaller set of taxa growing in the Kumpula Botanical Garden, Pagel’s λ test gave significant results only for I_chl_ and the fitted λ value was higher than for the set of taxa growing in the alpine botanical garden (**Table 1**). A phylogenetic-signal estimation method based on autocorrelation (Moran’s *I*), as well as Blomberg’s *K*, gave results that mirrored those of Pagel’s λ for plants growing in both botanical gardens, whereby the values were low but gave significant results for some leaf traits (**Table 1**). Some differences between the methods were found, whereby the highest value of Moran’s *I* amongst optically measured leaf traits was for I_ant_, and Blomberg’s *K* gave non-significant results for traits other than I_flav_ from the alpine botanical garden (**Table 1**; **Figure S13**). Graphical representations of the autocorrelation at different lags of distance (i.e. phylogenetic correlograms assessing the signal depth), indicated a weak positive autocorrelation with slightly shorter lags of distance and no negative autocorrelation with longer lags of distance for I_flav_, compared to I_chl_ and I_ant_, for the set of taxa from the alpine botanical garden (**Figure S14**). Local Moran’s *I* used to characterise local patterns in leaf traits, performed for the phylogeny of taxa from the alpine botanical garden, produced mostly local positive autocorrelations for I_flav_ (83.1%), I_chl_ (80.9%) and I_ant_ (91.1%) (**Table S6**). Based on local Moran’s *I*, we were able to distinguish taxa which mainly had high mean leaf trait values (I_flav_: *Alchemilla, Penstemon* and *Rhaponticum*; I_chl_: *Allium, Iris* and *Narcissus*; I_ant_: *Lonicera, Penstemon, Ribes* and *Vaccinium*) and others which mainly had low mean leaf trait values (I_flav_: *Cerastium, Hieracium*, some *Sedum* and many Poaceae species; I_chl_: *Lonicera, Ribes* and *Rubus*; I_ant_: *Allium* and *Iris*), all with mostly local positive autocorrelations (**Table S6**). Similarly to the larger phylogeny, local Moran’s *I* for mean I_flav_ from the phylogeny excluding shaded plants gave significant positive autocorrelations for many species from *Cerastium*, *Hieracium*, *Penstemon* and *Rhaponticum*, but also distinguished some other taxa than those in the analysis made for the larger phylogeny (**Table S6**). The same analysis repeated separately for the mean I_flav_ of taxa sampled in Kumpula Botanical Garden did not distinguish the same taxa as the phylogeny used for plants growing in the alpine botanical garden, although some similarities in local autocorrelation were found for I_chl_ (*Rubus* genus, *Iris* species) and I_ant_ (*Iris* species) (**Figure S15**; **Table S6**).

**Table 1:** Phylogenetic signal (Pagel’s λ) calculated for a phylogeny based on Jin and Qian (2019) mega-tree and optically measured mean leaf traits (I_flav,_ I_chl_ and I_ant_, all with arbitrary units).

	Site	Mean I_flav_	Mean I_chl_	Mean I_ant_
Fitted value of λ	Alpine botanical garden (*n* = 622)	0.56	0.30	0.49
Log-likelihood		17.02	-2110.09	1116.45
Log-likelihood (λ = 0)		59.39	56.16	88.38
Significance†		****	****	****
Moran’s *I*		0.04	0.04	0.07
Significance†		***	***	***
Blomberg’s *K*		0.03	0.01	0.02
Significance†		***	NS	NS
Fitted value of λ	Alpine botanical garden without shaded plants (*n* = 375)	0.41	0.28	0.46
Log-likelihood		77.15	-1266.20	657.00
Log-likelihood (λ = 0)		14.86	34.61	37.06
Significance†		***	****	****
Moran’s *I*		0.04	0.04	0.08
Significance†		***	***	***
Blomberg’s *K*		0.04	0.03	0.04
Significance†		**	NS	NS
Fitted value of λ	Kumpula Botanical Garden (*n* = 86)	0.15	0.55	0.05
Log-likelihood		-28.21	-285.58	161.87
Log-likelihood (λ = 0)		1.23	20.64	0.31
Significance†		NS	****	NS
Moran’s *I*		0.05	0.12	-0.02
Significance†		NS	**	NS
Blomberg’s *K*		0.02	0.08	0.03
Significance†		NS	**	NS

†Significance levels: NS ≥ 0.05, * <0.05, **≤0.01, ***≤0.001, ****≤0.0001.

Results for other methods (Moran’s *I* and Blomberg’s *K*) are shown for comparison (R package phytools; Keck et al., 2016). Leaf trait sampling was done for plants growing in the alpine botanical garden (Col du Lautaret, France) during summer of 2014, and in the Kumpula Botanical Garden (Helsinki, Finland) during summer of 2015.

## 4 Discussion

### 4.1 General patterns in optically measured leaf traits

The majority of plants had high I_flav_ (**Figure 1** and **S5**) and when data from both botanical gardens were combined, we found only three taxa in total with mean I_flav_ < 0.5 AU and 36 taxa with mean I_flav_ < 1 AU (**Table S3**). A reference point to interpreting these values may be taken from our earlier study of forest understoreys in Finland where seasonally lowest I_flav_ values were on average ≤ 0.5 AU during closed canopy phase (Hartikainen et al., 2020). Shaded plants have been shown to have constitutive phenolics present in leaf epidermis (Agati et al., 2020 and references therein). Although Dualex uses a reference beam and hence the index value is not directly comparable to absorbance, it may be roughly approximated that for instance at index value two, 99% of UV-A beam is attenuated (Aphalo, 2020). We also found a weak positive relationship between mean I_flav_ and UV irradiance received in plants’ local microhabitat (**Supplementary A2**). Comparing between the I_flav_ values of 27 species growing at high elevation and high latitude sites without accounting for local differences in solar radiation, eleven species showed no significant differences between the two sites, nine species had lower I_flav_ in Kumpula Botanical Garden and the other seven species lower I_flav_ in the alpine botanical garden (**Figure S16**). The significant but weak negative relationship between I_flav_ range (max-min) and mean I_flav_ among taxa (**Figure S9**), implies that certain taxa with a high I_flav_ may have less variation in their I_flav_. In line with this, some studies have reported a less pronounced response of UV-absorbing compounds to elevated UV-B radiation among high elevation species (reviewed by Turunen and Latola, 2005). Furthermore, some native mountain species lack flexibility in epidermal UV-A transmittance along elevational gradient compared to exotic species (Barnes et al., 2017). These combined results suggest that other drivers apart from local UV irradiance, may explain some differences in I_flav_ among taxa. These patterns of species’ I_flav_ concurred with an earlier study in identifying some species whose leaf flavonoids are relatively unresponsive to changes in their environment (Nybakken et al., 2004a): for example, we found that *Sanguisorba dodecandra* Moretti did not show differences in mean I_flav_ between our two sites and had a narrow range in I_flav_ (**Figure S16**). Differences in leaf flavonoids have also been found between *Vicia faba* L. accessions originating from regions of high and low UV radiation, that differed in responses to solar UV and blue light in their flavonoid profiles (Yan et al., 2019). Although compound-specific differences are beyond the scope of this study, these types of qualitative differences are likely to be present among the taxa in our dataset.

Unlike the generally high I_flav_ in our dataset, we found high leaf I_ant_ to be rare, and the node with relatively high I_ant_ had the largest neighbour distance in the SOM (**Figure 1**). In some taxa, an association has been found between flower colours produced by anthocyanins and floral concentrations of flavones, which may stem from enzyme sharing in the flavonoid pathway (Tripp et al., 2018). However, we generally did not find that leaf I_flav_ and I_ant_ were well correlated across our dataset, with the exception that among some *Penstemon* species (*P. ellipticus* Coult. & Fisher, *P. fruticosus* (Pursh) Greene, *P. fruticosus* var. *scouleri* (Lindl.) Cronquist and *P. newberryi* Gray) there was a positive local autocorrelation for both high I_flav_ and I_ant_ (**Table S6**). The lack of correlation between I_flav_ and I_ant_ may also stem from other interactions within the same shared biosynthetic pathway (discussed in Tripp et al., 2018, reviewed by Araguirang and Richter, 2022). Considering photoprotection strategy, our SOM linking chlorophyll fluorescence parameters with optically measured leaf traits, placed the taxa with highest I_ant_ and highest mean NPQ and Y(NPQ) in the same node, which might suggest coordinated activation of multiple protection mechanisms. It should be noted that studies sometimes report lower NPQ in anthocyanin-rich leaves, for instance in basil cultivars under high light (Tattini et al., 2014).

The relationship across taxa in I_flav_ values recorded between the two consecutive years was consistent, but those interannual differences found in I_flav_ may stem from variation in the weather, particularly temperature (Bilger et al., 2007; Coffey and Jansen, 2019) which also drives spring phenology affecting the plant development at the time of measurement. A study adjacent to the alpine botanical garden comparing four species differing in their specialisation towards snowbed habitats, reported high I_flav_ values in the early spring (mean I_flav_: 1.90-1.97 AU, Fernández-Marín et al., 2021) compared to our measurements of the same species in summer (mean I_flav_: 1.36-1.75 AU). This difference suggests that some seasonal changes in leaf flavonoid content may have occurred towards lower values from spring to summer.

Although *F*_v_ */F*_m_ was mostly high from plants in alpine botanical garden, some taxa experienced significant midday depression of *F*_v_ */F*_m_ producing a diurnal pattern (**Table S4**). This diurnal pattern is often recorded from plants under unobscured solar radiation, pointing to transient photoinhibition (Long et al., 1994). Sometimes *F*_v_ */F*_m_ depression has been recorded in mountain species (Fernández-Marín et al., 2020; Fernández-Marín et al., 2021), which could indicate a conservative strategy for light capture by avoiding potential damage during limiting conditions for carbon assimilation (Fernández-Marín et al., 2021). We found generally high NPQ, which appeared to be present particularly in those taxa with diurnally significant differences in *F*_v_ */F*_m_. Furthermore, SOMs distinguished some differences between nodes where groups of taxa (e.g. *Iris graminea* L. & *I. sibirica* L., *Rhododendron hirsutum* L.) had relatively low predawn *F*_v_ */F*_m_ and relatively high Y(NO), but not particularly high I_flav_. Flavonoids are suggested to be engaged in photoprotection following an increase from low to high solar irradiance (Talhouët et al., 2020), although chronic and acute exposure to UV-B radiation sometimes have different effects (Hideg et al., 2013). Our results suggest that the most common trait combination among plants from the 86 taxa tested, was high NPQ and high I_flav_ which potentially implies a strategy to avoid damage to PSII, while that group of taxa displaying relatively high Y(NO) and lowered *F*_v_ */F*_m_ may have suffered from photodamage, but interestingly without notable accumulation of leaf flavonoids.

### 4.2 Do optically measured leaf traits reflect climatic conditions at the place of origin?

We did not find a relationship between I_flav_ and climatic conditions at the original collection sites of plants (**Table S5**). This is surprising in light of previous findings that reported long-lasting population origin association (local adaptation) in flavonoids of *Pinus sylvestris* L. (Oleszek et al., 2002), population-specific differences in flavonoid profiles (mainly myricetin concentration) of *Pinus halepensis* Mill. (Kaundun et al., 1998) and some indication of differences among flavonoid compounds in response to UV-B treatments according to latitude of the population origin in two *Betula* species (Lavola, 1998). Furthermore, an increasing number of studies report transgenerational effects in response to UV-radiation (Yan et al., 2020; Jiang et al., 2021; Zhang et al., 2021). The accession-specific transgenerational effect on foliar flavonoids is illustrated in an experiment on *Vicia faba*, where exposure of parental plants to ambient UV radiation < 350 nm was shown to increase the induction of quercetin in the next generation in the presence of UV-B radiation (Yan et al., 2020). Greater long-term effects in leaf phenolics might be expected in trees because of their long generation times. Considering our setup in the botanical gardens, for instance, a selection of seeds from wild populations and their subsequent cultivation for eight generations altered the trait responses and fitness of the herbaceous plant species, in comparison to the wild population (Pizza et al., 2021). This type of an effect may have compounded any effect of original climate in our data. Nevertheless, acclimation of plants to a different environment in the botanical gardens also accentuates the importance of flexibility in leaf flavonoid accumulation. For example, taxon among the five highest mean I_flav_ (*Primula nutans* subsp. *finmarchica* var. *jokelae*) originated from the most northern site (**Figure S17, Figure S15**), and despite this subspecies showing signs of adaptational lag, it was still performing relatively well in its current location in Helsinki, Finland (Hällfors et al., 2020). Some differences in flavonoids can be compound-specific or otherwise difficult to detect, such as contrasting patterns in concentrations of different flavonoid and phenolic compounds of vascular plants with elevation, and in comparison to phenolic compounds of lichens with elevation (Asplund et al., 2021). The relatively low resolution of the climate data for our purpose may have failed to reflect the micro-environment at the plant origin, potentially explaining why original climate did not show a relationship across our species pool. For instance, weather station data does not always represent the temperature micro-environments of small alpine plants nearby (Scherrer and Körner, 2010; Körner and Hiltbrunner, 2018). Furthermore, comparing native range of the taxa may have been interesting since previous studies on coloration of autumnal leaves in tree and woody species suggest geographic origin, and namely their differences in autumnal solar radiation and temperature, to be important for explaining differences in leaf anthocyanins (reviewed by Renner and Zohner, 2019).

Climatic patterns in I_chl_ were clearer than for I_flav_, whereby I_chl_ decreased with the latitude and increased with the elevation of plants’ site of origin (**Table S5**). We detected a positive relationship between I_chl_ and dry weight of the leaf-disks obtained from a subset of taxa (**Supplementary A3**). The specific leaf area (SLA) is often found to decrease with elevation (Hultine and Marshall, 2000), so this relationship may partly explain the increase in I_chl_ with elevation.

Many of the original collections sites of plants studied here are changing from a cold or polar to a temperate climate according to the projected Köppen-Geiger classification for 2071-2100 (Beck et al., 2018) (**Figures S18, S19**), although there is considerable uncertainty about whether changes in climate will cause solar radiation to increase or decrease in the Alps (Gobiet et al., 2014). A recent study of alpine plant species range shifts (limits, optima and distribution) suggested that historically low-elevation species increased their abundance and range more effectively than high-elevation species, likely in response to current warming and nitrogen deposition (Rumpf et al., 2018). The increase in UV radiation over a rise of 1000 m elevation (annual altitude effect of total global radiation) in the Alps may be 11-19% (Blumthaler et al., 1992), meaning that any migration uphill driven by changes in climate could expose plants to a higher maximum UV-B radiation. I_flav_ varied according to micro-environmental differences in UV irradiance (**Supplementary A2**), suggesting that most taxa express flexibility in I_flav_ in response to their ambient conditions. Given these patterns of results and relationships, it is likely that biotic interactions and temperature changes will impose greater restrictions on high-elevation plant success and fitness (HilleRisLambers et al., 2013; Mondoni et al., 2015; Rumpf et al., 2018) than exposure to higher UV radiation.

### 4.3 Importance of the relatedness of taxa for the optically measured leaf trait distribution

In our analysis looking for general patterns in the phylogeny, both the phylogenetic signal and correlograms gave significant results, but respectively showed low λ values and weak autocorrelations, which suggests that phylogenetic relatedness alone failed to explain the observed trait values (**Table 1**; **Figure S14**). The relationship we describe in the previous section between solar irradiance at local microhabitats and mean I_flav_, might be expected to reduce the phylogenetic signal. It is not unprecedented that there is no phylogenetic relationship among secondary metabolites, for instance individual pyrrolizidine alkaloids, have been found to be randomly distributed among *Jacobaea* species (Chen et al., 2020). Despite these findings, the significant positive autocorrelations with short lags of distance we obtained in correlograms for I_flav_ indicated signal between closely related species. Accordingly, we were able to distinguish a local positive autocorrelation in I_flav_ (local Moran’s *I*) among some closely related species or infraspecific taxa (e.g. *Alchemilla, Penstemon, Rhaponticum, Cerastium* and *Hieracium*) (**Table S6**). Although our analysis of methanol extracts of leaf phenolic compounds (**Supplementary A3**) did not formally identify similarities in the shape of UV absorption spectra at the genus level, in most cases these spectra bore a strong resemblance to each other among species from the same genus. Previous studies have reported detailed patterns in flavones within one genus, *Ruellia*, identifying a local autocorrelation associated with clades from xeric and dry environments (Tripp et al., 2018). This suggests that using a larger number of species per genus could better disentangle phylogenetic patterns in leaf flavonoids. Interestingly, we found fewer negative than positive local autocorrelations, suggesting that diverging species-specific patterns in I_flav_ were relatively uncommon among close relatives, perhaps pointing to the importance of the underlying evolution of the flavonoid synthetic pathway in conditioning the nature of this response. Similarities in trait values of related taxa were found even among taxa which originates from very different geographical locations: *Rhaponticum heleniifolium* subsp. *bicknellii* (Briq.) Greuter with range in Maritime Alps and *Rhaponticum carthamoides* (Willd.) Iljin originating from Central Asia to Siberia, both with high I_flav_. The identification from our analysis of those taxa which had either mainly high or mainly low I_flav_, may indicate that their leaf flavonoid accumulation was relatively inflexible. Taxa restricted to low I_flav_ values (e.g. some *Hieracium*) could face constraints against migrating to environments receiving higher UV radiation, if their other compensatory photoprotective mechanisms are not effective, whereas those genera which maintained high I_flav_ may better tolerate an increase in UV radiation.

The intermediate values of fitted λ (i.e. 0 < λ < 1) given by our analysis for the entire phylogeny indicate that the observed trait values of taxa were less similar than expected for this phylogeny under a Brownian motion model of trait evolution. These results were significant when testing against λ = 0, in other words a situation where relatedness explains none of the trait similarities (Revell, 2012; Swenson, 2014). Overall, this large phylogenetic analysis of plants from two botanical gardens suggests that our results were robust, although the analysis of I_flav_ and I_ant_ from a smaller set of taxa growing at high latitude gave a lower fitted λ and insignificant results. This difference in outcome may relate to the finding that most methods for inferring phylogenetic signals are sensitive to the size of the phylogeny, with smaller sizes usually increasing the uncertainty (Münkemüller et al., 2012). Although many polytomies were present in our phylogeny, Pagel’s λ should be robust against potential issues stemming from the use of incompletely resolved phylogenies, and phylogenies with suboptimal branch-length information (Molina-Venegas and Rodríguez, 2017). Our results do not allow for conclusions concerning the mechanism underlying the patterns found (i.e. low λ values, but similarities in related species from some genera in the local analysis), as evolutionary processes have different relationships with the phylogenetic signal under simulations (Revell et al., 2008). For instance, stabilizing selection towards a single optimum may lead to low phylogenetic species’ covariance (Hansen and Martins, 1996; Revell et al., 2008). Overall, these results suggest that among some specific taxa, relatedness, for instance among those genera we identified with mainly high I_flav,_ may explain some similarities in optically measured leaf traits, while in other taxa we failed to detect such a relationship, suggesting that other drivers were probably more important in determining these trait values.

## 5 Conclusions

We used optical measurements to survey the leaf traits of a set of over 600 taxa, mostly growing in high-elevation environments, to better understand potential constraints set by their relatedness, and climate of origin, particularly considering the influence of UV radiation in these contexts. Large surveys covering many taxa enhance trait databases, in this case for optically measured leaf traits, and will help to improve our knowledge of plant responses to climate change and received UV radiation. It was not possible to give a definitive hierarchy of drivers of I_flav_ among taxa, as the patterns we found were complex, and likely involved multiple drivers of flavonoid synthesis and accumulation. We conclude that these leaf traits showed flexibility in most taxa we examined, and in most plants responded to the exposure to sunlight in their immediate micro-environment. We did not identify potential for constraints in leaf flavonoid accumulation set by plants’ climate of origin. Within some species from the same genus, I_flav_ had relatively similar values, suggesting some constraints associated with phylogenetic relatedness on the extent of their induction could be responsible for the lack of variation. These taxa merit further study, when considering whether an increase in exposure to UV radiation, through migration or a change in climate *in situ*, could reduce their fitness.

## Data availability statement

Dataset containing records of the optically measured mean leaf traits from all plant taxa may be found from https://doi.org/10.5281/zenodo.7398305.

## Author contributions

SH and TR conceived the ideas; SH designed the field methodology; SH collected and analysed all data, except the spectroradiometer data collected by TR; SH wrote the manuscript and TR supervised all stages. Both authors gave editorial input and final approval for publication.

## References

[B1] Agati G. Brunetti C. Fini A. Gori A. Guidi L. Landi M.. (2020). Are flavonoids effective antioxidants in plants? twenty years of our investigation. Antioxidants 9 (11), 1098. doi: 10.3390/antiox9111098 33182252 PMC7695271

[B2] Agati G. Stefano G. Biricolti S. Tattini M. (2009). Mesophyll distribution of a’ntioxidant’ flavonoid glycosides in ligustrum vulgare leaves under contrasting sunlight irradiance. Ann. Bot. 104 (5), 853–861. doi: 10.1093/aob/mcp177 19633310 PMC2749533

[B3] Anselin L. (1995). Local indicators of spatial association-LISA. Geographical Anal. 27 (2), 93–115. doi: 10.1111/j.1538-4632.1995.tb00338.x

[B4] Aphalo P. J. (2020). Absorbance, absorptance and friends. UV4Plants Bull. 2020 (1), 45–60. doi: 10.19232/uv4pb.2020.1.12

[B5] Araguirang G. E. Richter A. S. (2022). Activation of anthocyanin biosynthesis in high light – what is the initial signal? New Phytol. 23 (6), 2037–2043. doi: 10.1111/nph.18488 36110042

[B6] Asplund J. Zuijlen K. Roos R. E. Birkemoe T. Klanderud K. Lang S. I.. (2021). Contrasting responses of plant and lichen carbon-based secondary compounds across an elevational gradient. Funct. Ecol. 35 (2), 330–341. doi: 10.1111/1365-2435.13712

[B7] Barnes P. W. Flint S. D. Caldwell M. M. (1987). Photosynthesis damage and protective pigments in plants from a latitudinal Arctic/Alpine gradient exposed to supplemental UV-b radiation in the field. Arctic Alpine Res. 19 (1), 21. doi: 10.2307/1550996

[B8] Barnes P. W. Flint S. D. Tobler M. A. Ryel R. J. (2016b). Diurnal adjustment in ultraviolet sunscreen protection is widespread among higher plants. Oecologia 181 (1), 55–63. doi: 10.1007/s00442-016-3558-9 26809621

[B9] Barnes P. W. Kersting A. R. Flint S. D. Beyschlag W. Ryel R. J. (2013). Adjustments in epidermal UV-transmittance of leaves in sun-shade transitions. Physiologia Plantarum 149 (2), 200–213. doi: 10.1111/ppl.12025 23330642

[B10] Barnes P. W. Ryel R. J. Flint S. D. (2017). UV Screening in native and non-native plant species in the tropical alpine: Implications for climate change-driven migration of species to higher elevations. Front. Plant Sci. 8. doi: 10.3389/fpls.2017.01451 PMC5572244 28878792

[B11] Barnes P. W. Tobler M. A. Keefover-Ring K. Flint S. D. Barkley A. E. Ryel R. J.. (2016a). Rapid modulation of ultraviolet shielding in plants is influenced by solar ultraviolet radiation and linked to alterations in flavonoids. Plant Cell Environ. 39 (1), 222–230. doi: 10.1111/pce.12609 26177782

[B12] Beckmann M. Václavík T. Manceur A. M. Šprtová L. von Wehrden H. Welk E.. (2014). glUV: A global UV-b radiation data set for macroecological studies. Methods Ecol. Evol. 5 (4), 372–383. doi: 10.1111/2041-210X.12168

[B13] Beck H. E. Zimmermann N. E. McVicar T. R. Vergopolan N. Berg A. Wood E. F. (2018). Present and future köppen-Geiger climate classification maps at 1-km resolution. Sci. Data 5 (1), 180214. doi: 10.1038/sdata.2018.214 30375988 PMC6207062

[B14] Benjamini Y. Hochberg Y. (1995). Controlling the false discovery rate: A practical and powerful approach to multiple testing. J. R. Stat. Society: Ser. B (Methodological) 57 (1), 289–300. doi: 10.1111/j.2517-6161.1995.tb02031.x

[B15] Berardi A. E. Fields P. D. Abbate J. L. Taylor D. R. (2016). Elevational divergence and clinal variation in floral color and leaf chemistry in *Silene vulgaris* . Am. J. Bot. 103 (8), 1508–1523. doi: 10.3732/ajb.1600106 27519429

[B16] Bilger W. Rolland M. Nybakken L. (2007). UV Screening in higher plants induced by low temperature in the absence of UV-b radiation. Photochemical Photobiological Sci. 6 (2), 190. doi: 10.1039/b609820g 17277843

[B17] Blomberg S. P. Garland T. (2002). Tempo and mode in evolution: Phylogenetic inertia, adaptation and comparative methods: Phylogenetic inertia. J. Evolutionary Biol. 15 (6), 899–910. doi: 10.1046/j.1420-9101.2002.00472.x

[B18] Blomberg S. P. Garland T. Ives A. R. (2003). Testing for phylogenetic signal in comparative data: behavioral traits are more labile. Evolution 57 (4), 717–745. doi: 10.1111/j.0014-3820.2003.tb00285.x 12778543

[B19] Blumthaler M. (2012). “Solar radiation of the high Alps,” in Plants in alpine regions. Ed. Lütz C. (Vienna: Springer). doi: 10.1007/978-3-7091-0136-0

[B20] Blumthaler M. Ambach W. Rehwald W. (1992). Solar UV-a and UV-b radiation fluxes at two alpine stations at different altitudes. Theor. Appl. Climatology 46 (1), 39–44. doi: 10.1007/BF00866446

[B21] Cerovic Z. G. Masdoumier G. Ghozlen N. B. Latouche G. (2012). A new optical leaf-clip meter for simultaneous non-destructive assessment of leaf chlorophyll and epidermal flavonoids. Physiologia Plantarum 146 (3), 251–260. doi: 10.1111/j.1399-3054.2012.01639.x 22568678 PMC3666089

[B22] Cerovic Z. G. Ounis A. Cartelat A. Latouche G. Goulas Y. Meyer S.. (2002). The use of chlorophyll fluorescence excitation spectra for the non-destructive *in situ* assessment of UV-absorbing compounds in leaves: UV-absorption spectra estimated from fluorescence. Plant Cell Environ. 25 (12), 1663–1676. doi: 10.1046/j.1365-3040.2002.00942.x

[B23] Chen I.-C. Hill J. K. Ohlemuller R. Roy D. B. Thomas C. D. (2011). Rapid range shifts of species associated with high levels of climate warming. Science 333 (6045), 1024–1026. doi: 10.1126/science.1206432 21852500

[B24] Chen Y. Mulder P. P. J. Schaap O. Memelink J. Klinkhamer P. G. L. Vrieling K. (2020). The evolution of pyrrolizidine alkaloid diversity among and within *Jacobaea* species. J. Systematics Evol. 60 (2), 361–376. doi: 10.1111/jse.12671

[B25] Coffey A. Jansen M. A. K. (2019). Effects of natural solar UV-b radiation on three arabidopsis accessions are strongly affected by seasonal weather conditions. Plant Physiol. Biochem. 134, 64–72. doi: 10.1016/j.plaphy.2018.06.016 29958807

[B26] Cornelissen J. H. C. Makoto K. (2014). Winter climate change, plant traits and nutrient and carbon cycling in cold biomes. Ecol. Res. 29 (4), 517–527. doi: 10.1007/s11284-013-1106-1

[B27] Demmig-Adams B. Adams W. W. (2006). Photoprotection in an ecological context: The remarkable complexity of thermal energy dissipation. New Phytol. 172 (1), 11–21. doi: 10.1111/j.1469-8137.2006.01835.x 16945085

[B28] de Villemereuil P. Mouterde M. Gaggiotti O. E. Till-Bottraud I. (2018). Patterns of phenotypic plasticity and local adaptation in the wide elevation range of the alpine plant *Arabis alpina* . J. Ecol. 106 (5), 1952–1971. doi: 10.1111/1365-2745.12955

[B29] Fernández-Marín B. Nadal M. Gago J. Fernie A. R. López-Pozo M. Artetxe U.. (2020). Born to revive: Molecular and physiological mechanisms of double tolerance in a paleotropical and resurrection plant. New Phytol. 226 (3), 741–759. doi: 10.1111/nph.16464 32017123

[B30] Fernández-Marín B. Sáenz-Ceniceros A. Solanki T. Robson T. M. García-Plazaola J. I. (2021). Alpine forbs rely on different photoprotective strategies during spring snowmelt. Physiologia Plantarum, 1–12. doi: 10.1111/ppl.13342 33483975

[B31] Fick S. E. Hijmans R. J. (2017). WorldClim 2: New 1-km spatial resolution climate surfaces for global land areas. Int. J. Climatology 37, 4302–4315. doi: 10.1002/joc.5086

[B32] Gittleman J. L. Kot M. (1990). Adaptation: Statistics and a null model for estimating phylogenetic effects. Systematic Zoology 39 (3), 227. doi: 10.2307/2992183

[B33] Gobiet A. Kotlarski S. Beniston M. Heinrich G. Rajczak J. Stoffel M. (2014). 21st century climate change in the European Alps–a review. Sci. Total Environ. 493, 1138–1151. doi: 10.1016/j.scitotenv.2013.07.050 23953405

[B34] Grant R. H. (1999). Potential effect of soybean heliotropism on ultraviolet-b irradiance and dose. Agron. J. 91 (6), 1017–1023. doi: 10.2134/agronj1999.9161017x

[B35] Grotewold E. (2006). The science of flavonoids (New York, NY: Springer).

[B36] Guo W.-D. Guo Y.-P. Liu J.-R. Mattson N. (2004). Midday depression of photosynthesis is related with carboxylation efficiency decrease and D1 degradation in bayberry (Myrica rubra) plants. Scientia Hortic. 123 (2), 188–196. doi: 10.1016/j.scienta.2009.07.014

[B37] Hällfors M. Lehvävirta S. Aandahl T. Lehtimäki I.-M. Nilsson L. O. Ruotsalainen A.. (2020). Translocation of an arctic seashore plant reveals signs of maladaptation to altered climatic conditions. PeerJ 8, e10357. doi: 10.7717/peerj.10357 33240662 PMC7682418

[B38] Hansen T. F. Martins E. P. (1996). Translating between microevolutionary process and macroevolutionary patterns: the correlation structure of interspecific data. Evolution 50 (4), 1404–1417. doi: 10.1111/j.1558-5646.1996.tb03914.x 28565714

[B39] Hartikainen S. M. Pieristè M. Lassila J. Robson T. M. (2020). Seasonal patterns in spectral irradiance and leaf UV-a absorbance under forest canopies. Front. Plant Sci. 10. doi: 10.3389/fpls.2019.01762 PMC7040076 32133015

[B40] Harvey P. H. Pagel M. (1991). The comparative method in evolutionary biology (Oxford: Oxford University Press).

[B41] He X. Burgess K. S. Yang X. Ahrends A. Gao L. Li D. (2019). Upward elevation and northwest range shifts for alpine meconopsis species in the himalaya–hengduan mountains region. Ecol. Evol. 9 (7), 4055–4064. doi: 10.1002/ece3.5034 31015987 PMC6467849

[B42] Hideg É. Jansen M. A. K. Strid Å. (2013). UV-B exposure, ROS, and stress: Inseparable companions or loosely linked associates? Trends Plant Sci. 18 (2), 107–115. doi: 10.1016/j.tplants.2012.09.003 23084465

[B43] HilleRisLambers J. Harsch M. A. Ettinger A. K. Ford K. R. Theobald E. J. (2013). How will biotic interactions influence climate change-induced range shifts? Ann. New York Acad. Sci. 1297 (1), 112–125. doi: 10.1111/nyas.12182 23876073

[B44] Hultine K. R. Marshall J. D. (2000). Altitude trends in conifer leaf morphology and stable carbon isotope composition. Oecologia 123 (1), 32–40. doi: 10.1007/s004420050986 28308741

[B45] Hutchinson M. Xu T. Houlder D. Nix H. McMahon J. (2009). ANUCLIM 6.0 user’s guide (Fenner School of Environment and Society: Australian National University).

[B46] Jaakola L. Hohtola A. (2010). Effect of latitude on flavonoid biosynthesis in plants: Effect of latitude on flavonoid biosynthesis. Plant Cell Environ. 33, 1239–1247. doi: 10.1111/j.1365-3040.2010.02154.x 20374534

[B47] Jiang J. Liu J. Sanders D. Qian S. Ren W. Song J.. (2021). UVR8 interacts with *de novo* DNA methyltransferase and suppresses DNA methylation in arabidopsis. Nat. Plants 7 (2), 184–197. doi: 10.1038/s41477-020-00843-4 33495557 PMC7889724

[B48] Jin Y. Qian H. (2019). V.PhyloMaker: An r package that can generate very large phylogenies for vascular plants. Ecography 42 (8), 1353–1359. doi: 10.1111/ecog.04434 PMC9363651 35967255

[B49] Kaundun S. S. Lebreton P. Fady B. (1998). Geographical variability of pinus halepensis mill. as revealed by foliar flavonoids. Biochem. Systematics Ecol. 26 (1), 83–96. doi: 10.1016/S0305-1978(97)00092-6

[B50] Keck F. Rimet F. Bouchez A. Franc A. (2016). Phylosignal: An r package to measure, test, and explore the phylogenetic signal. Ecol. Evol. 6 (9), 2774–2780. doi: 10.1002/ece3.2051 27066252 PMC4799788

[B51] Klughammer C. Schreiber U. (2008). Complementary PS II quantum yields calculated from simple fluorescence parameters measured by PAM fluorometry and the saturation pulse method. PAM Appl. Notes 1, 27–35.

[B52] Kohonen T. (1982). Self-organized formation of topologically correct feature maps. Biol. Cybernetics 43 (1), 59–69. doi: 10.1007/BF00337288

[B53] Körner C. Hiltbrunner E. (2018). The 90 ways to describe plant temperature. Perspect. Plant Ecology Evol. Systematics 30, 16–21. doi: 10.1016/j.ppees.2017.04.004

[B54] Körner C. Riedl S. Keplinger T. Richter A. Wiesenbauer J. Schweingruber F.. (2019). Life at 0 °C: The biology of the alpine snowbed plant soldanella pusilla. Alpine Bot. 129 (2), 63–80. doi: 10.1007/s00035-019-00220-8

[B55] Koyama K. Takemoto S. (2014). Morning reduction of photosynthetic capacity before midday depression. Sci. Rep. 4 (1), 4389. doi: 10.1038/srep04389 24633128 PMC3955906

[B56] Kriticos D. J. Jarošik V. Ota N. (2014). Extending the suite of bioclim variables: A proposed registry system and case study using principal components analysis. Methods Ecol. Evol. 5 (9), 956–960. doi: 10.1111/2041-210X.12244

[B57] Laitinen M.-L. Julkunen-Tiitto R. Rousi M. (2002). Foliar phenolic composition of European white birch during bud unfolding and leaf development. Physiologia Plantarum 114 (3), 450–460. doi: 10.1034/j.1399-3054.2002.1140315.x 12060268

[B58] Lavola A. (1998). Accumulation of flavonoids and related compounds in birch induced by UV-b irradiance. Tree Physiol. 18 (1), 53–58. doi: 10.1093/treephys/18.1.53 12651299

[B59] Long S. P. Humphries S. Falkowski P. G. (1994). Photoinhibition of photosynthesis in nature. Annu. Rev. Plant Physiol. Plant Mol. Biol. 45 (1), 633–662. doi: 10.1146/annurev.pp.45.060194.003221

[B60] Losos J. B. (2008). Phylogenetic niche conservatism, phylogenetic signal and the relationship between phylogenetic relatedness and ecological similarity among species. Ecol. Lett. 11 (10), 995–1003. doi: 10.1111/j.1461-0248.2008.01229.x 18673385

[B61] LUOMUS, Finnish Museum of natural history (2021). Kumpula botanic garden: Introduction to botanic garden. Available at: https://www.luomus.fi/en/introduction-botanic-garden.

[B62] Maxwell K. Johnson G. N. (2000). Chlorophyll fluorescence–a practical guide. J. Exp. Bot. 51 (345), 659–668. doi: 10.1093/jexbot/51.345.659 10938857

[B63] Merilä J. (2012). Evolution in response to climate change: In pursuit of the missing evidence. BioEssays 34 (9), 811–818. doi: 10.1002/bies.201200054 22782862

[B64] Merilä J. Hendry A. P. (2014). Climate change, adaptation, and phenotypic plasticity: The problem and the evidence. Evolutionary Appl. 7 (1), 1–14. doi: 10.1111/eva.12137 PMC3894893 24454544

[B65] Molina-Venegas R. Rodríguez M.Á. (2017). Revisiting phylogenetic signal; strong or negligible impacts of polytomies and branch length information? BMC Evolutionary Biol. 17 (1), 53. doi: 10.1186/s12862-017-0898-y PMC5312541 28201989

[B66] Mondoni A. Pedrini S. Bernareggi G. Rossi G. Abeli T. Probert R. J.. (2015). Climate warming could increase recruitment success in glacier foreland plants. Ann. Bot. 116, 907–916. doi: 10.1093/aob/mcv101 26133689 PMC4640126

[B67] Münkemüller T. Lavergne S. Bzeznik B. Dray S. Jombart T. Schiffers K.. (2012). How to measure and test phylogenetic signal. Methods Ecol. Evol. 3 (4), 743–756. doi: 10.1111/j.2041-210X.2012.00196.x

[B68] Murchie E. H. Lawson T. (2013). Chlorophyll fluorescence analysis: A guide to good practice and understanding some new applications. J. Exp. Bot. 64 (13), 3983–3998. doi: 10.1093/jxb/ert208 23913954

[B69] Nybakken L. Aubert S. Bilger W. (2004a). Epidermal UV-screening of arctic and alpine plants along a latitudinal gradient in Europe. Polar Biol. 27 (7), 391–398. doi: 10.1007/s00300-004-0601-9

[B70] Nybakken L. Bilger W. Johanson U. Björn L. O. Zielke M. Solheim B. (2004b). Epidermal UV-screening in vascular plants from Svalbard (Norwegian Arctic). Polar Biol. 27 (7), 383–390. doi: 10.1007/s00300-004-0602-8

[B71] Oleszek W. Stochmal A. Karolewski P. Simonet A. M. Macias F. A. Tava A. (2002). Flavonoids from pinus sylvestris needles and their variation in trees of different origin grown for nearly a century at the same area. Biochem. Systematics Ecol. 30 (11), 1011–1022. doi: 10.1016/S0305-1978(02)00060-1

[B72] Ort D. R. (2001). When there is too much light. Plant Physiol. 125 (1), 29–32. doi: 10.1104/pp.125.1.29 11154289 PMC1539318

[B73] Pagel M. (1999). Inferring the historical patterns of biological evolution. Nature 401 (6756), 877–884. doi: 10.1038/44766 10553904

[B74] Parmesan C. Hanley M. E. (2015). Plants and climate change: Complexities and surprises. Ann. Bot. 116 (6), 849–864. doi: 10.1093/aob/mcv169 26555281 PMC4640131

[B75] Pizza R. Espeland E. Etterson J. (2021). Eight generations of native seed cultivation reduces plant fitness relative to the wild progenitor population. Evolutionary Appl. 14 (7), 1816–1829. doi: 10.1111/eva.13243 PMC8288025 34295366

[B76] Qian H. Jin Y. (2016). An updated megaphylogeny of plants, a tool for generating plant phylogenies and an analysis of phylogenetic community structure. J. Plant Ecol. 9 (2), 233–239. doi: 10.1093/jpe/rtv047

[B77] Renner S. S. Zohner C. M. (2019). The occurrence of red and yellow autumn leaves explained by regional differences in insolation and temperature. New Phytol. 224 (4), 1464–1471. doi: 10.1111/nph.15900 31070794

[B78] Revell L. J. (2012). Phytools: An r package for phylogenetic comparative biology (and other things): *phytools: R package* . Methods Ecol. Evol. 3 (2), 217–223. doi: 10.1111/j.2041-210X.2011.00169.x

[B79] Revell L. J. (2013). Two new graphical methods for mapping trait evolution on phylogenies. Methods Ecol. Evol. 4 (8), 754–759. doi: 10.1111/2041-210X.12066

[B80] Revell L. J. Harmon L. J. Collar D. C. (2008). Phylogenetic signal, evolutionary process, and rate. Systematic Biol. 57 (4), 591–601. doi: 10.1080/10635150802302427 18709597

[B81] Rozema J. Chardonnens A. Tosserams M. Hafkenscheid R. Bruijnzeel S. (1997). “Leaf thickness and UV-b absorbing pigments of plants in relation to an elevational gradient along the blue mountains, Jamaica,” in UV-B and biosphere. Eds. Rozema J. Gieskes W. W. C. Van De Geijn S. C. Nolan C. De Boois H. (Netherlands: Springer), 150–159. doi: 10.1007/978-94-011-5718-6_14

[B82] Rumpf S. B. Hülber K. Klonner G. Moser D. Schütz M. Wessely J.. (2018). Range dynamics of mountain plants decrease with elevation. Proc. Natl. Acad. Sci. 115 (8), 1848–1853. doi: 10.1073/pnas.1713936115 29378939 PMC5828587

[B83] Scherrer D. Körner C. (2010). Infra-red thermometry of alpine landscapes challenges climatic warming projections. Global Change Biol. 16, 2602–2613. doi: 10.1111/j.1365-2486.2009.02122.x

[B84] Scherrer D. Körner C. (2011). Topographically controlled thermal-habitat differentiation buffers alpine plant diversity against climate warming. J. Biogeography 38 (2), 406–416. doi: 10.1111/j.1365-2699.2010.02407.x

[B85] Searles P. S. Flint S. D. Caldwell M. M. (2001). A meta-analysis of plant field studies simulating stratospheric ozone depletion. Oecologia 127 (1), 1–10. doi: 10.1007/s004420000592 28547159

[B86] Smith S. A. Brown J. W. (2018). Constructing a broadly inclusive seed plant phylogeny. Am. J. Bot. 105 (3), 302–314. doi: 10.1002/ajb2.1019 29746720

[B87] Streb P. Cornic G. (2012). “Photosynthesis and antioxidative protection in alpine herbs,” in Plants in alpine regions. Ed. Lütz C. (Vienna: Springer). doi: 10.1007/978-3-7091-0136-0

[B88] Swenson N. G. (2014). Functional and phylogenetic ecology in r (New York, NY: Springer). doi: 10.1007/978-1-4614-9542-0

[B89] Talhouët A. Meyer S. Baudin X. Streb P. (2020). Dynamic acclimation to sunlight in an alpine plant, *Soldanella alpina* l. Physiologia Plantarum 168 (3), 563–575. doi: 10.1111/ppl.12982 31090072

[B90] Tattini M. Landi M. Brunetti C. Giordano C. Remorini D. Gould K. S.. (2014). Epidermal coumaroyl anthocyanins protect sweet basil against excess light stress: Multiple consequences of light attenuation. Physiologia Plantarum 152 (3), 585–598. doi: 10.1111/ppl.12201 24684471

[B91] Tripp E. A. Zhuang Y. Schreiber M. Stone H. Berardi A. E. (2018). Evolutionary and ecological drivers of plant flavonoids across a large latitudinal gradient. Mol. Phylogenet. Evol. 128, 147–161. doi: 10.1016/j.ympev.2018.07.004 30017824

[B92] Turunen M. Latola K. (2005). UV-B radiation and acclimation in timberline plants. Environ. pollut. 137 (3), 390–403. doi: 10.1016/j.envpol.2005.01.030 16005753

[B93] Watermann L. Y. Hock M. Blake C. Erfmeier A. (2020). Plant invasion into high elevations implies adaptation to high UV-b environments: A multi-species experiment. Biol. Invasions 22 (3), 1203–1218. doi: 10.1007/s10530-019-02173-9

[B94] Wehrens R. Kruisselbrink J. (2018). Flexible self-organizing maps in kohonen 3.0. J. Stat. Software 87 (7), 1–18. doi: 10.18637/jss.v087.i07

[B95] Wickham H. (2016). Ggplot2: elegant graphics for data analysis (New York: Springer-Verlag).

[B96] Wielgolaski F. E. Karlsen S. R. (2007). Some views on plants in polar and alpine regions. Rev. Environ. Sci. Biotechnol. 6 (1–3), 33–45. doi: 10.1007/s11157-006-0014-z

[B97] Williams W. E. Gorton H. L. Witiak S. M. (2003). Chloroplast movements in the field: Chloroplast movements in the field. Plant Cell Environ. 26 (12), 2005–2014. doi: 10.1046/j.0016-8025.2003.01117.x

[B98] Woods E. C. Hastings A. P. Turley N. E. Heard S. B. Agrawal A. A. (2012). Adaptive geographical clines in the growth and defense of a native plant. Ecol. Monogr. 82 (2), 149–168. doi: 10.1890/11-1446.1

[B99] Wróblewska A. Mirski P. (2018). From past to future: Impact of climate change on range shifts and genetic diversity patterns of circumboreal plants. Regional Environ. Change 18 (2), 409–424. doi: 10.1007/s10113-017-1208-3

[B100] Yan Y. Stoddard F. L. Neugart S. Oravec M. Urban O. Sadras V. O.. (2020). The transgenerational effects of solar short-UV radiation differed in two accessions of vicia faba l. from contrasting UV environments. J. Plant Physiol. 248, 153145. doi: 10.1016/j.jplph.2020.153145 32145578

[B101] Yan Y. Stoddard F. L. Neugart S. Sadras V. O. Lindfors A. Morales L. O.. (2019). Responses of flavonoid profile and associated gene expression to solar blue and UV radiation in two accessions of *Vicia faba* l. from contrasting UV environments. Photochemical Photobiological Sci. 18 (2), 434–447. doi: 10.1039/C8PP00567B 30629071

[B102] Zanne A. E. Tank D. C. Cornwell W. K. Eastman J. M. Smith S. A. FitzJohn R. G.. (2014). Three keys to the radiation of angiosperms into freezing environments. Nature 506, 89–92. doi: 10.1038/nature12872 24362564

[B103] Zhang X. Li C. Tie D. Quan J. Yue M. Liu X. (2021). Epigenetic memory and growth responses of the clonal plant glechoma longituba to parental recurrent UV-b stress. Funct. Plant Biol 48, 827–838. doi: 10.1071/FP20303 33820599

[B104] Ziska L. H. Teramura A. H. Sullivan J. H. (1992). Physiological sensitivity of plants along an elevational gradient to UV-b radiation. Am. J. Bot. 79 (8), 863–871. doi: 10.1002/j.1537-2197.1992.tb13667.x

