# Supplementary material for: The roles of species’ relatedness and climate of origin in determining optical leaf traits over a large set of taxa growing at high elevation and high latitude
Source: Front Plant Sci. 2022 Dec 16;13:1058162. doi: 10.3389/fpls.2022.1058162 (PMC9800846; doi:10.3389/fpls.2022.1058162)

<u>Supplementary Material Appendix 1</u>: Figures (S1-S19) and Tables (S1-S6)

Figures (S1-S19)

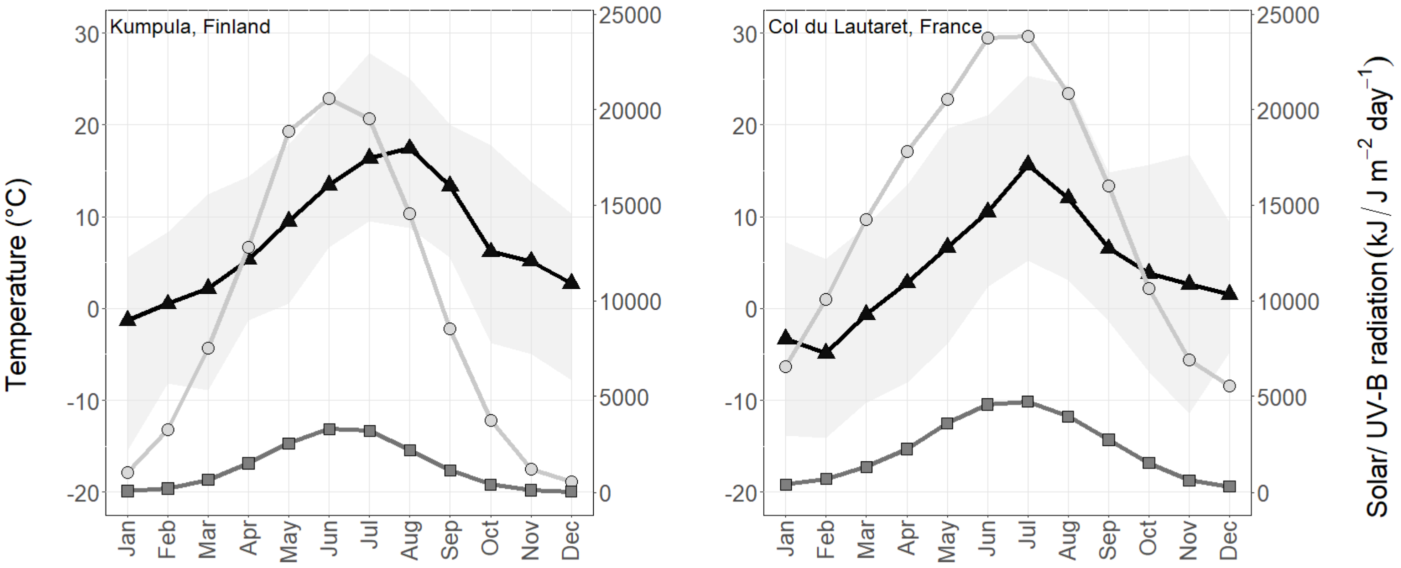

Figure S1. Monthly mean air temperature for 2015 (mean: black line with triangles, and range as grey shaded area), monthly mean solar radiation (kJ m^{-2} day^{-1}, light grey line with circular points) and UV-B radiation (J m^{-2} day^{-1}, dark grey line with square points) at the study sites. Air temperature data were obtained from weather stations within ~ 500 m (Finland) and 50 m (France) distance. Solar radiation data were obtained from WorldClim database based on observations over years 1970-2000 (Fick & Hijmans, 2017). Monthly mean UV-B radiation data over years 2004-2013 were obtained from Beckmann et al. (2014) climatology.

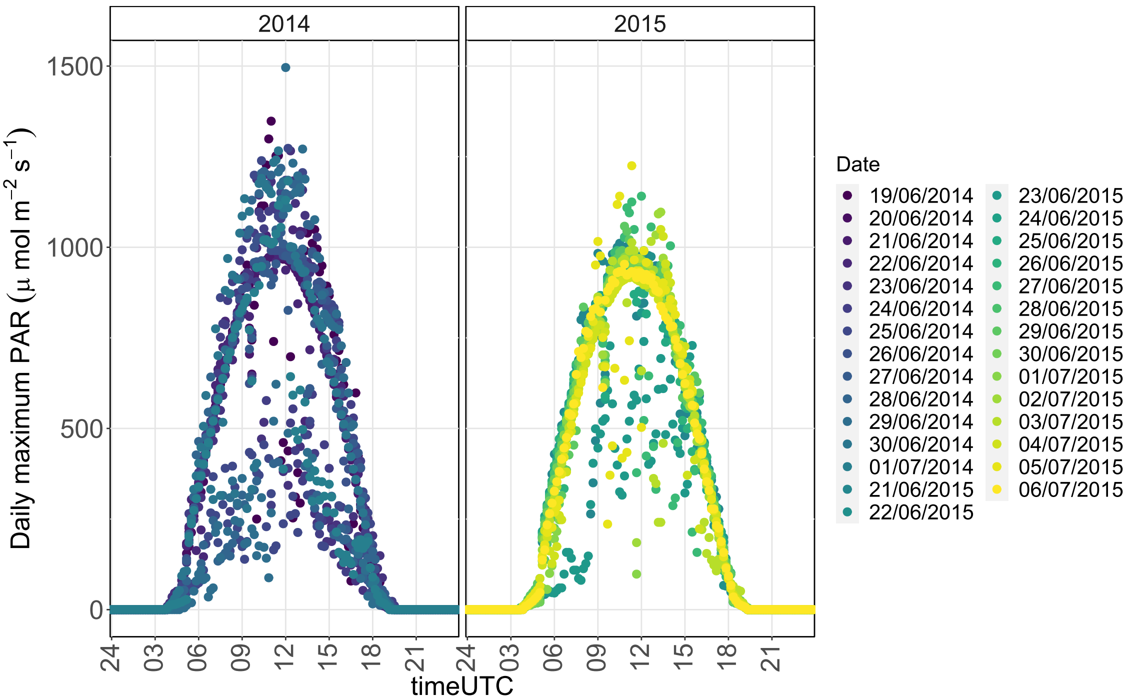

Figure S2. Daily maximum photosynthetically active radiation (PAR) (e-METSYS/JFAS weather station with PMA2132 PAR Sensor, Solar Light Co., Glenside, PA, USA) from the alpine botanical garden (Col du Lautaret, France) over the period when plant traits were sampled in 2014 and 2015. Sampling was done around solar noon (approximately ± 3 h) and overcast weather was avoided where possible.

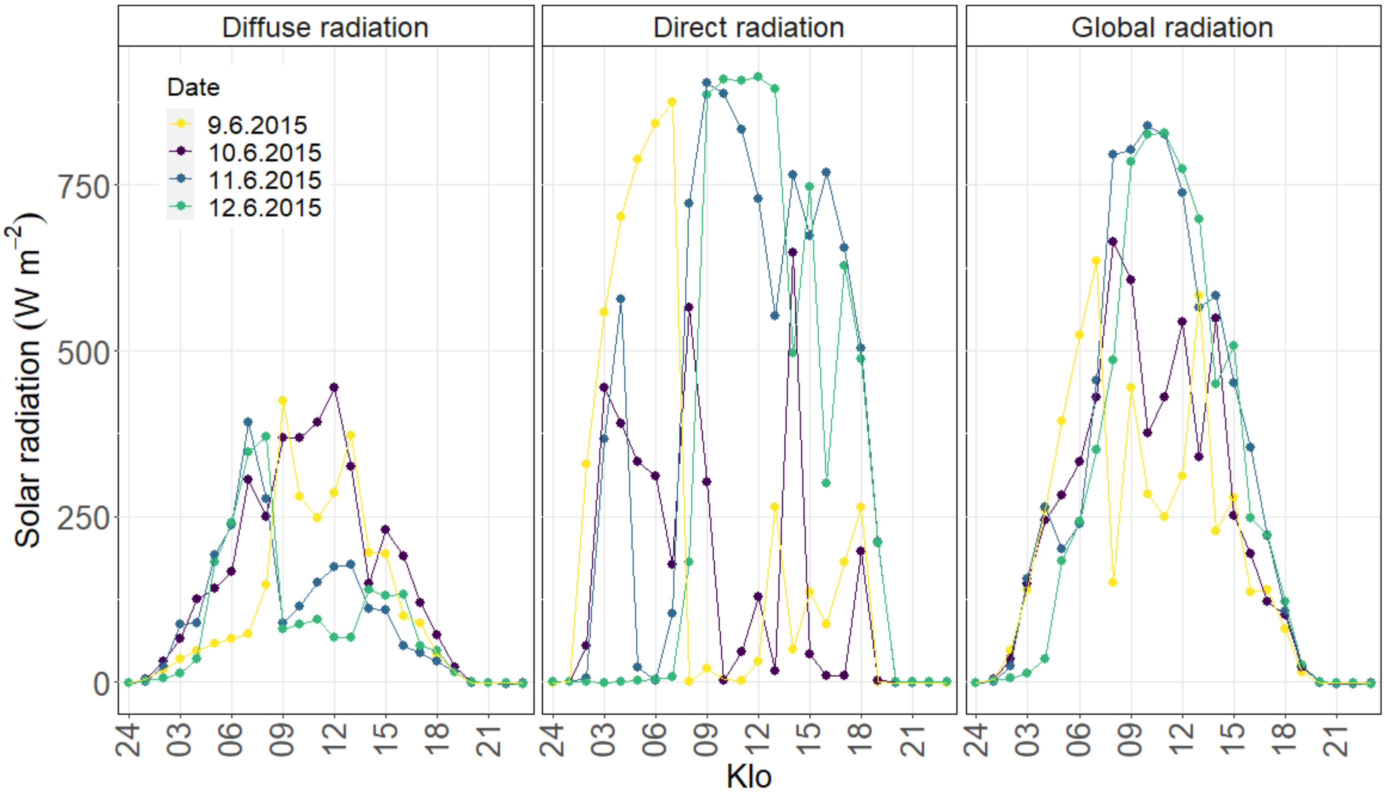

Figure S3. Diffuse, direct and global solar radiation adjacent to the Kumpula Botanical Garden (Helsinki, Finland) during sampling dates in 2015. Data were obtained from the Finnish Meteorological Institute's weather station located in Kumpula, Helsinki. Diffuse and global radiation are measured for a horizontal, while direct radiation is measured for a surface perpendicular to the direct beam.

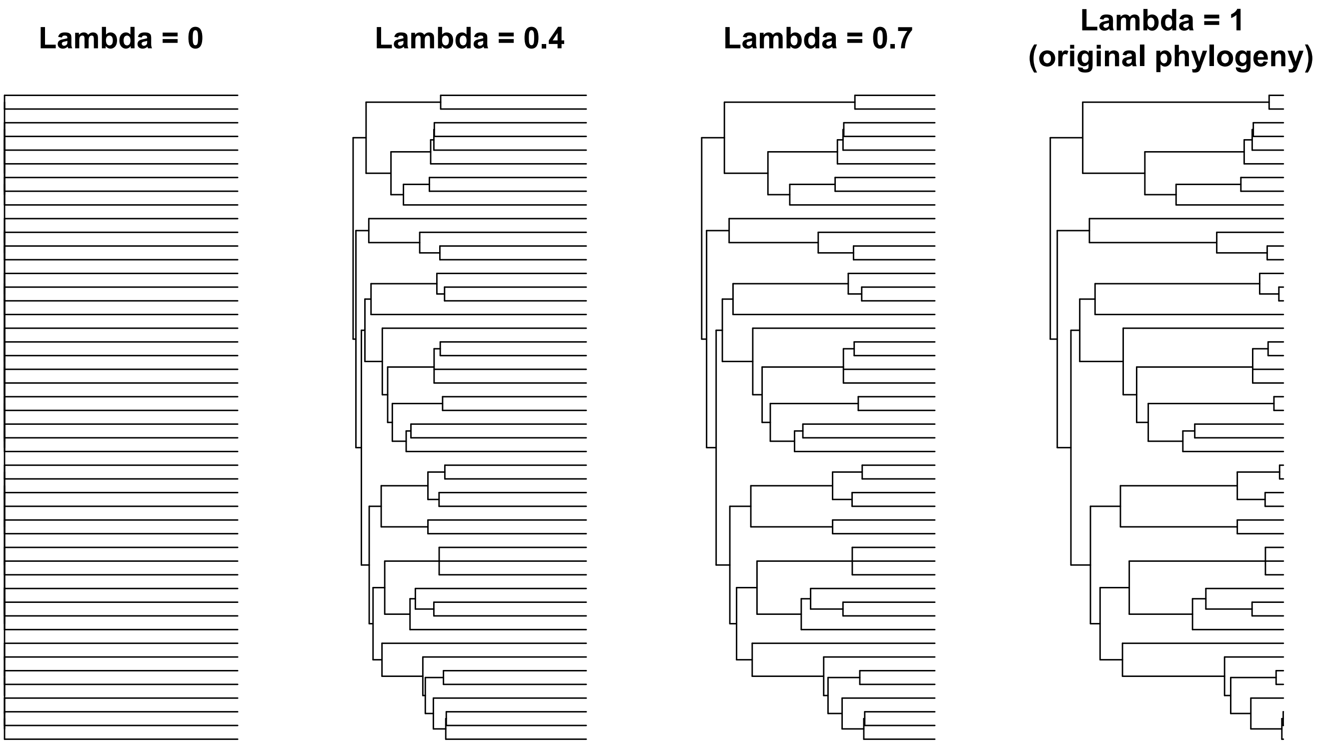

Figure S4. Figure illustrating the transformation of a phylogeny according to lambda (λ) value. In Pagel's lambda test for a phylogenetic signal, the fitted λ value is found through optimization (R package phytools, R function phylosig, Revell, 2012 & 2013) and the resulting transformed phylogeny best explains the trait values at the tips under a Brownian motion model of trait evolution. As lambda value decreases, internal nodes are moved more basal and this increases the distance to most recent common ancestor with the expectation that trait values become more dissimilar between any sister lineages (Pagel, 1999; Swenson, 2004). The plotted phylogeny used for 48 taxa from the alpine botanical garden is based on an existing mega-tree (GBOTB.extended.tre) and methodology (scenario 1) from Jin and Qian (2019). Lambda values between 0.4 to 0.7 represent the actual fitted λ values in this and other used phylogenies found in our study.

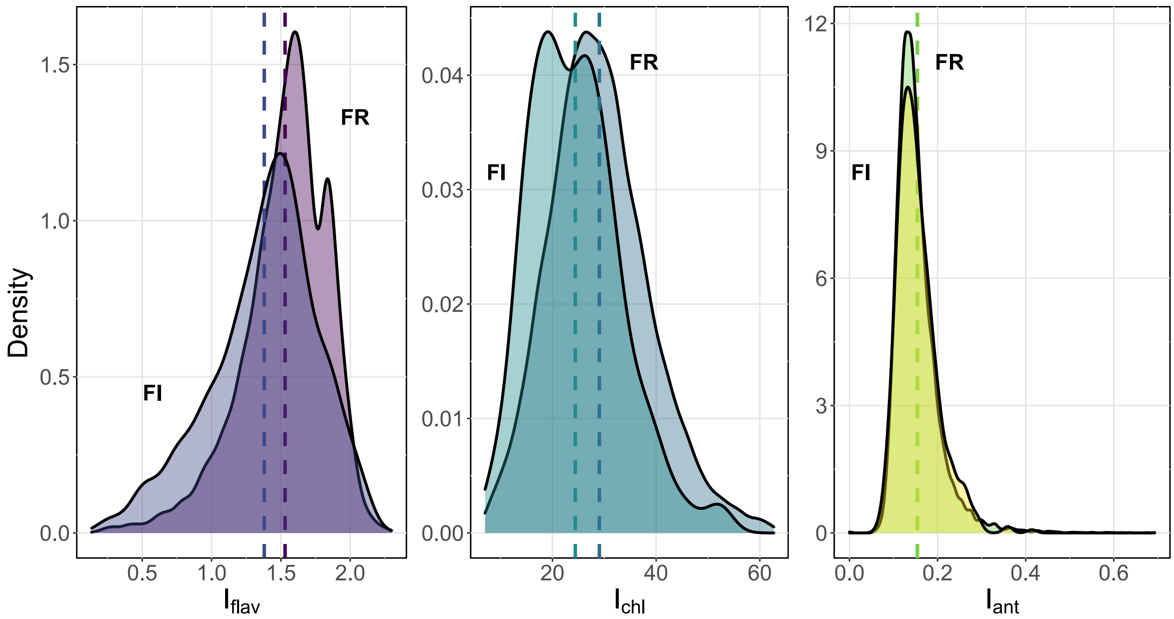

Figure S5. Kernel density and mean (dashed lines) for optically measured leaf traits (I_{flav}, I_{chl} and I_{ant}; arbitrary units) from all 672 taxa or developmental stages (10 172 measurements) sampled from alpine botanical garden (**FR**), and from all 86 taxa (501 measurements) sampled from Kumpula Botanical Garden (**FI**). Mean I_{ant} is same for both datasets from France and Finland so only one dashed line is plotted.

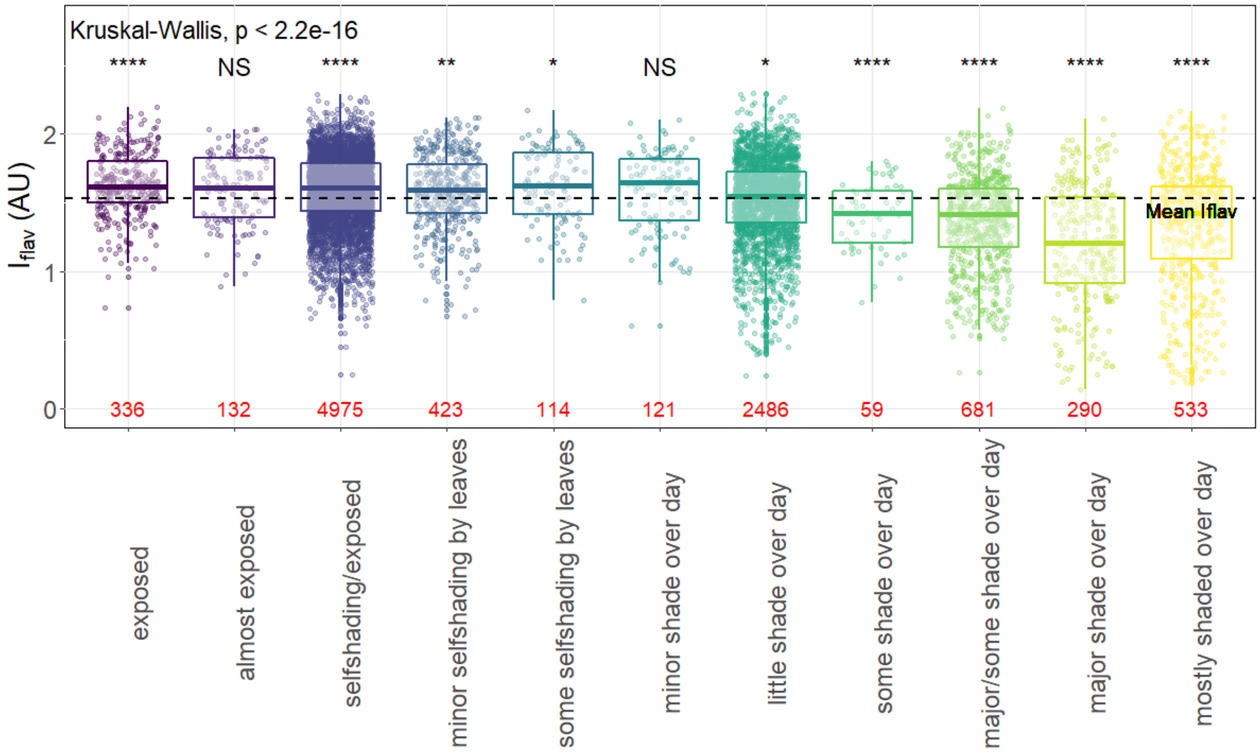

Figure S6. Mean flavonol/flavone index (I_{flav}, Arbitrary Unit) of leaves divided according to categorised plant light condition (microclimate ranked in order from exposed to mostly shaded over the day) measured in the alpine botanical garden in 2014. Red numbers below boxplots refers to the number of measurements. R function geom_jitter was used to avoid overplotting, and the non-parametric Kruskal-Wallis test result is shown. Significance levels for non-parametric Wilcoxon rank sum test comparing each group's I_{flav} against all groups' (mean I_{flav}: dotted horizontal line) are shown as: * <0.05, **≤0.01, ***≤0.001, ****≤0.0001. The *p* -values were adjusted using Benjamini and Hochberg (1995) correction method.

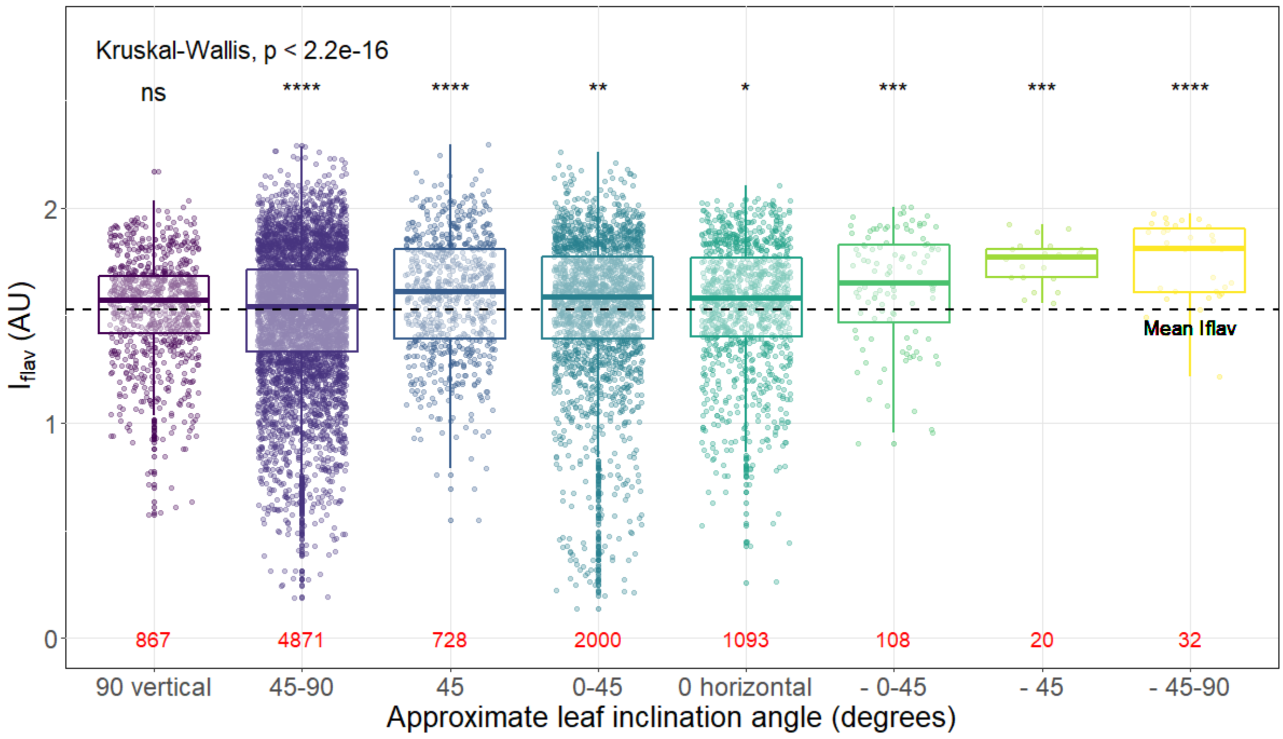

Figure S7. Mean flavonol/flavone index (I_{flav}, Arbitrary Unit) of leaves divided according to approximate categorised leaf inclination angle (from 90° vertical to below horizontal i.e. - 45-90°) measured in the alpine botanical garden (Col du Lautaret, France) in 2014. Inclination angle was censured as the angle between horizontal and the leaf blade. Format as in Figure S5.

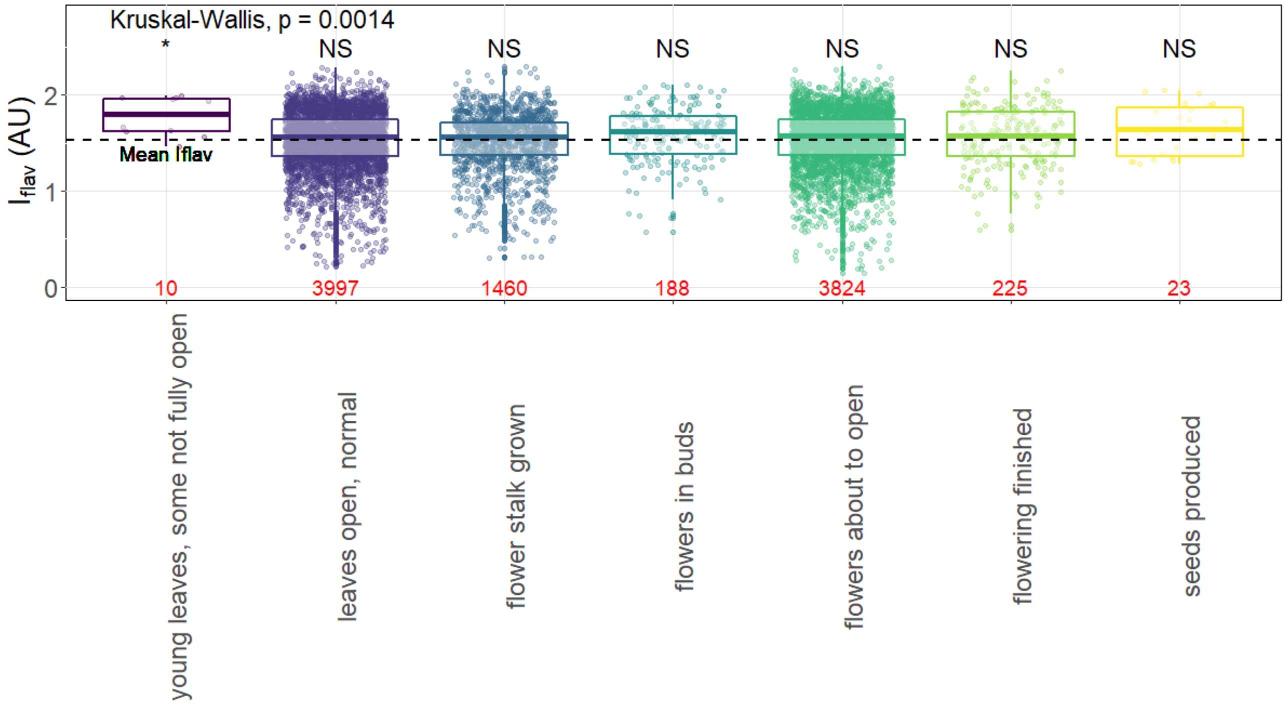

Figure S8. Mean flavonol/flavone index (I_{flav}, Arbitrary Unit) of leaves divided according to categorised plant phenology (from newly produced leaves to seeds produced) measured in the alpine botanical garden (Col du Lautaret, France) in 2014. Format as in Figure S5.

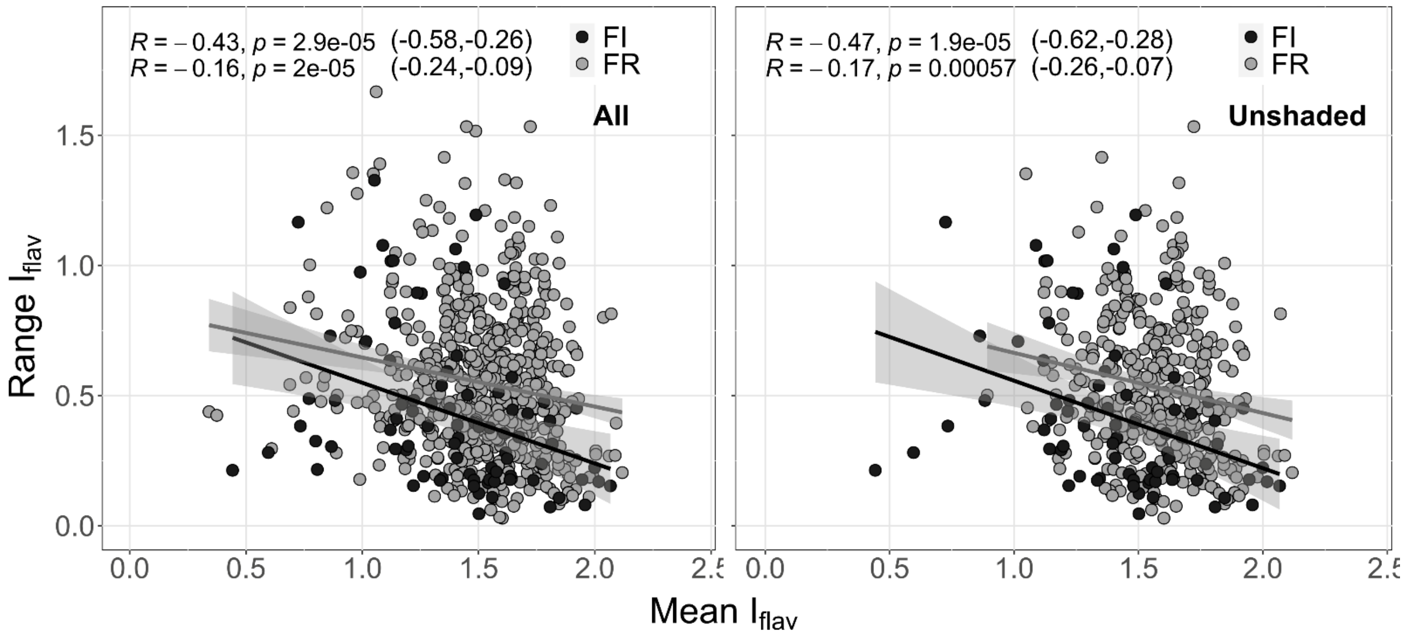

Figure S9. Relationship between mean flavonol/flavone index (I_{flav}, arbitrary units) and its range (maximum - minimum I_{flav} value) for taxa growing in Kumpula Botanical Garden (Helsinki, Finland; 86 taxa, black points) and in alpine botanical garden (Col du Lautaret, France; 672 taxa, grey points). Left panel shows the relationship for all plants with Spearman's rank correlation (Finland above, France below), respective *p*- value and 95% bootstrap confidence intervals (in parenthesis, R function spearman.ci from R package RVAideMemoire). The linear trend is given to the cloud of points for each site with a 95% CI (grey band). The right panel shows the relationship for plants which experienced at most minor shading over the day.

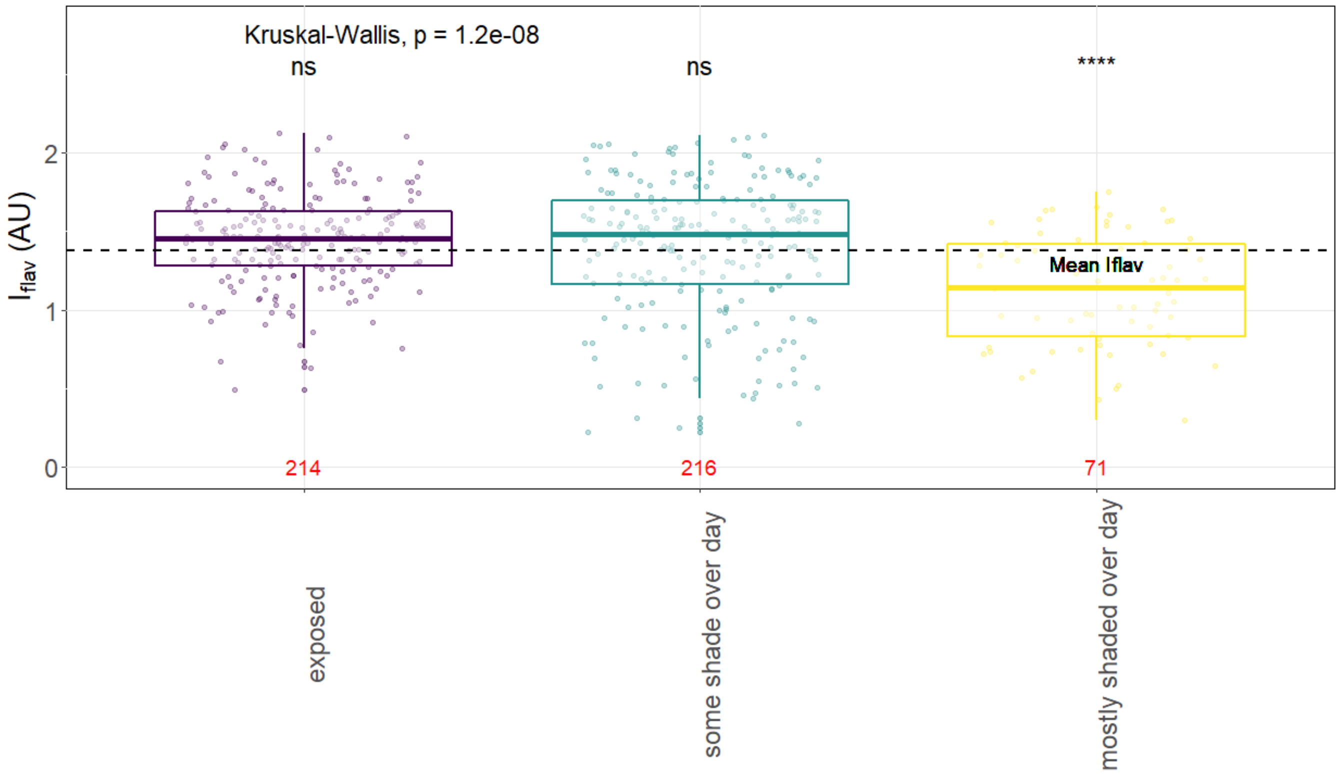

Figure S10. Mean flavonol/flavone index (I_{flav}, Arbitrary Unit) of leaves divided according to categorised plant light condition (from exposed to mostly shaded over day) measured from plants growing in Kumpula Botanical Garden (Helsinki, Finland) in 2015. Format as Figure S6.

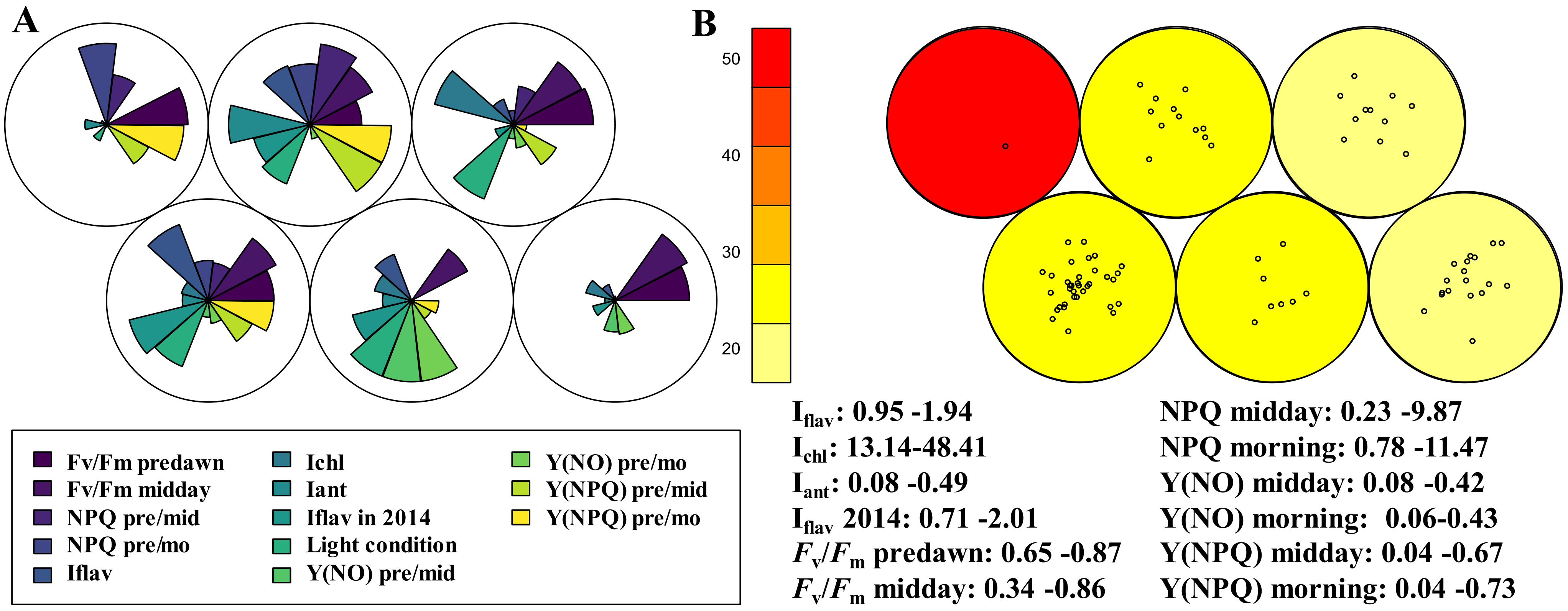

Figure S11. Relative differences in leaf traits from 84 taxa and *Eryngium alpinum* seedlings, in the alpine botanical garden (Col du Lautaret, France) shown with hexagonally arranged Kohonen self-organising map (SOM). Plants were sampled for optically measured leaf traits (I_{flav}, I_{chl}, I_{ant}), chlorophyll fluorescence parameters (predawn and dark-adapted midday F_v/F_m, NPQ (predawn F_m & either mid-morning or midday F_m'), Y(NO) (predawn F_m & either mid-morning or midday F') and Y(NPQ) (predawn F_m & either mid-morning or midday F', F_m') and for their median categorised light conditions. Panel **A** shows relative differences (radius of each wedge) in mean leaf traits and light conditions as colours, panel **B** shows loadings of the nodes i.e. number of taxa grouped in the node as points, and neighbour distance (sum of the distances to all immediate neighbours) as colour scale with red for highest distance. The range in mean optically measured leaf traits used are given below.

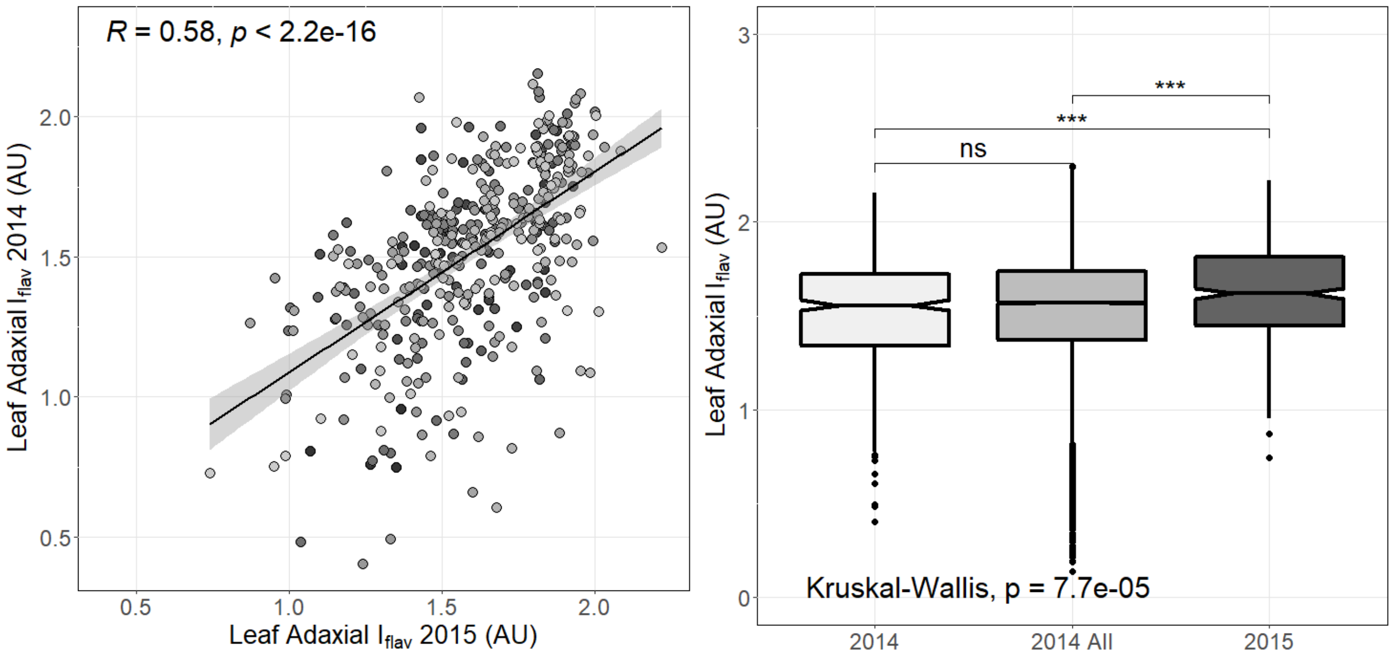

Figure S12. Differences in optically measured flavonol/flavone index (I_{flav}, Arbitrary Unit) between two consecutive years (2014 & 2015) from the alpine botanical garden. **Left panel** shows a linear trendline plotted to cloud of points with different grey colours representing 86 taxa ($n \geq 4$), with 95% confidence intervals. A Spearman's rank correlation coefficient and corresponding *p*- value are given. **Right panel** shows median I_{flav} (with notched 95 % CI) for 86 taxa sampled in both years, and for all sampled 672 taxa or developmental stages in 2014. The *p*-value obtained from a non-parametric Kruskal-Wallis test is shown, and significant differences between measurements tested with Wilcoxon rank sum test using Benjamini and Hochberg (1995) correction method to adjust *p* -values are indicated as: * <0.05, **≤0.01, ***≤0.001, ****≤0.0001.

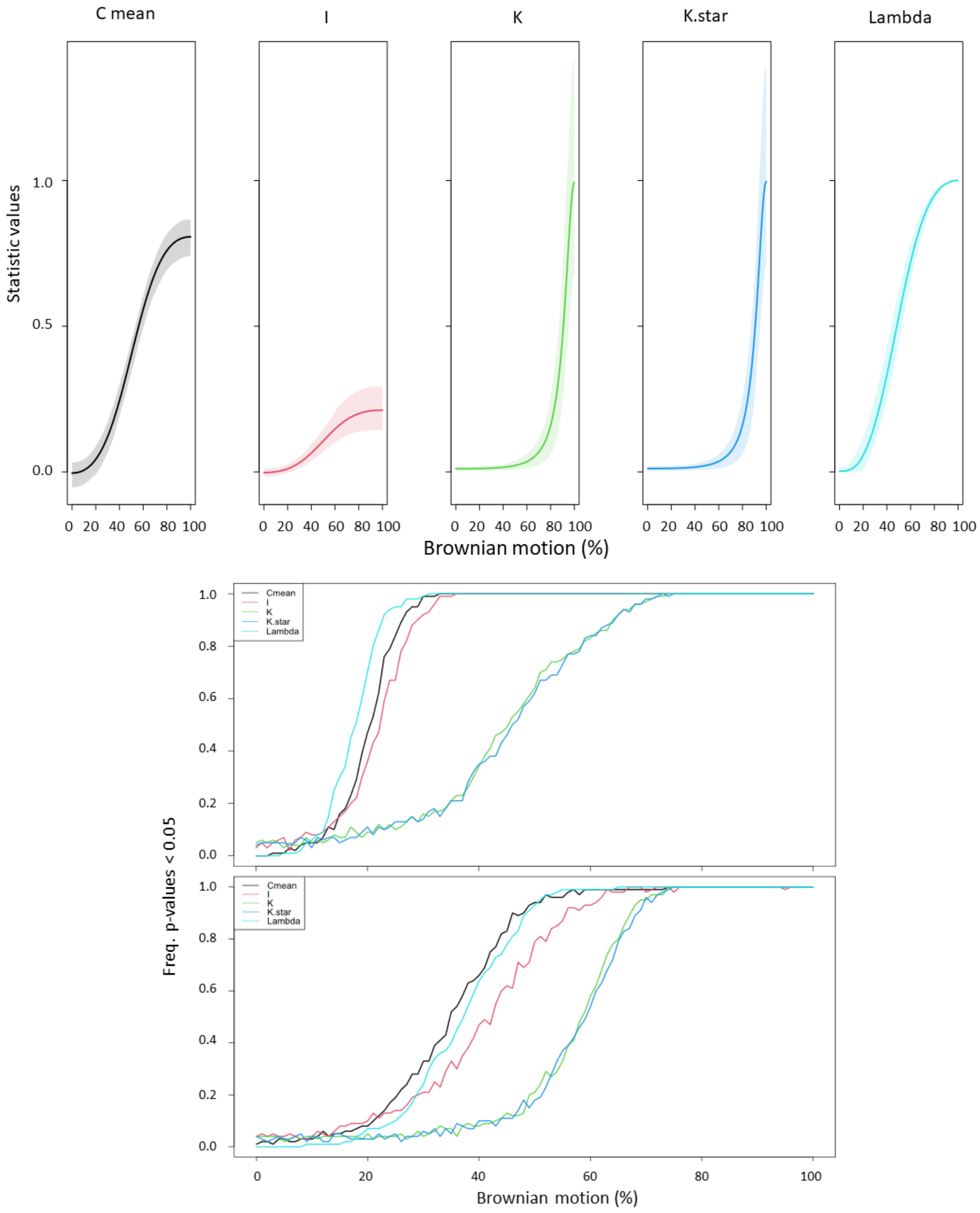

Figure S13. Simulation used to explore the behaviour of various methods (Abouheif's C mean = Cmean, Moran's *I* = I, Blomberg's *K* = K, Blomberg's *K** = K.star, Pagel's lambda = Lambda) estimating the phylogenetic signal of the phylogenies used in the study. The simulated traits with differing strength of Brownian motion were used for a tree generated using 622 taxa growing in the alpine botanical garden (Col du Lautaret, France; two figures above), and from 86 taxa growing in Kumpula Botanical Garden (Helsinki, Finland; shown below). Simulations and plots were created using R function phyloSim (R package phylosignal: Keck et al., 2016, approach in Münkemüller et al., 2012).

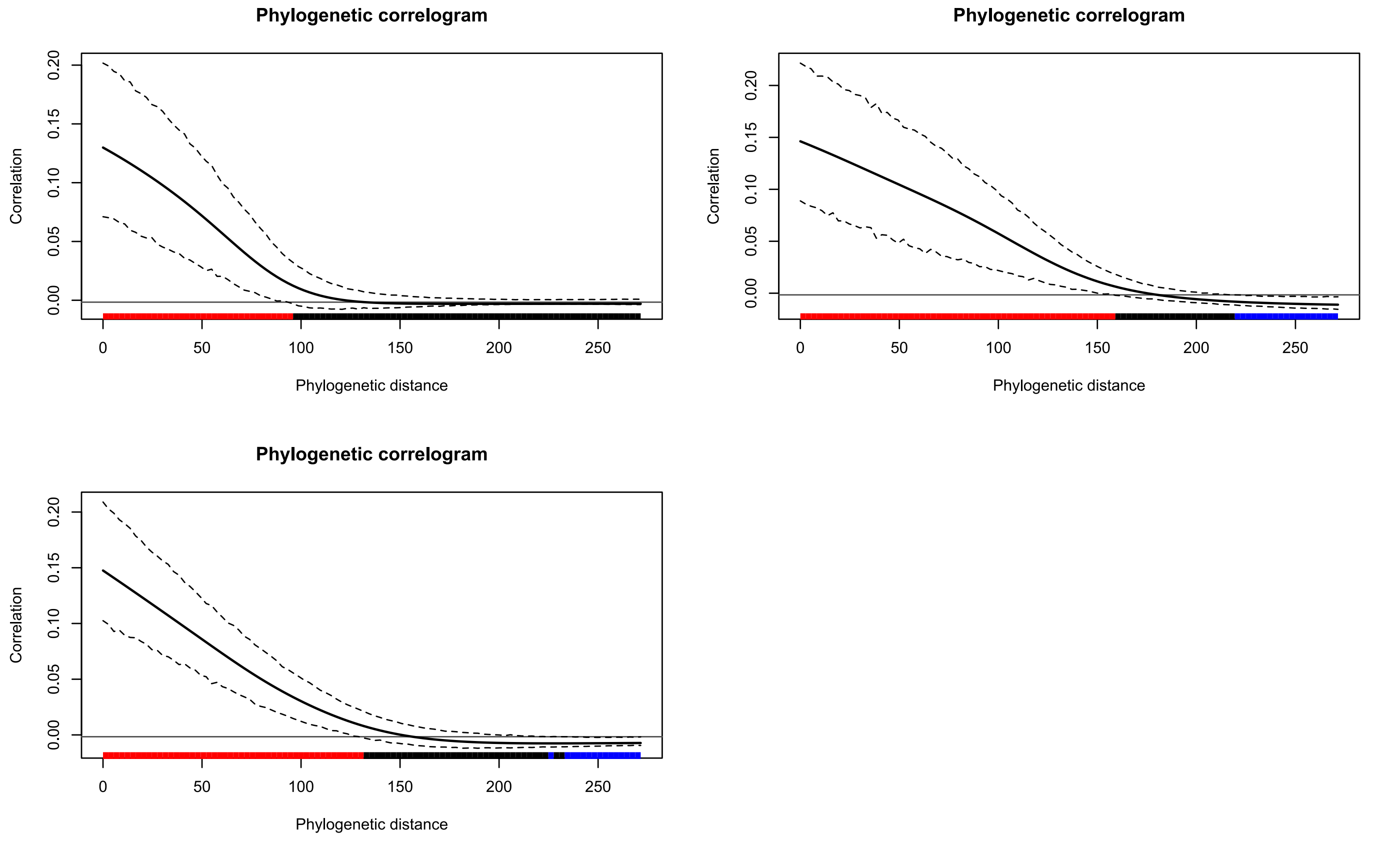

Figure S14. Correlograms for optically measured leaf traits (from upper left: I_{flav}, I_{chl} and I_{ant}, all in arbitrary units) for the phylogeny used for 622 taxa growing in the alpine botanical garden (Col du Lautaret, France). R function phyloCorrelogram (R package phylosignal: Keck et al., 2016) was used with a patristic distance matrix, and a 95% confidence envelope (created by the R function automatically, using non-parametric bootstrap re-sampling). The black horizontal line indicates the expected value of Moran's *I* when no phylogenetic autocorrelation is found (H0). The red bar indicates a significant positive and the blue line a significant negative autocorrelation based on confidence intervals.

Figure S15 (Next page). Phylogeny used for the studied 86 taxa from the Kumpula Botanical Garden (Helsinki, Finland) based on mega-tree and methodology (scenario 1) from Jin & Qian (2019) is plotted with optically measured mean trait values (I_{flav} = **A**, I_{chl} = **B** and I_{ant} = **C**, all in arbitrary units) as colours (blue to red from minimum to maximum) by using R package phytools (Revell 2012 & 2013). Colour bar length is a scale for branch lengths (million years, Myr). The plotting method estimates ancestral trait values but the estimate of uncertainty is not shown, and ancestral values are mainly given for visual clarity. Infraspecific taxa (i.e. subspecies and variety) were included in the phylogeny by combining them with their parental species.

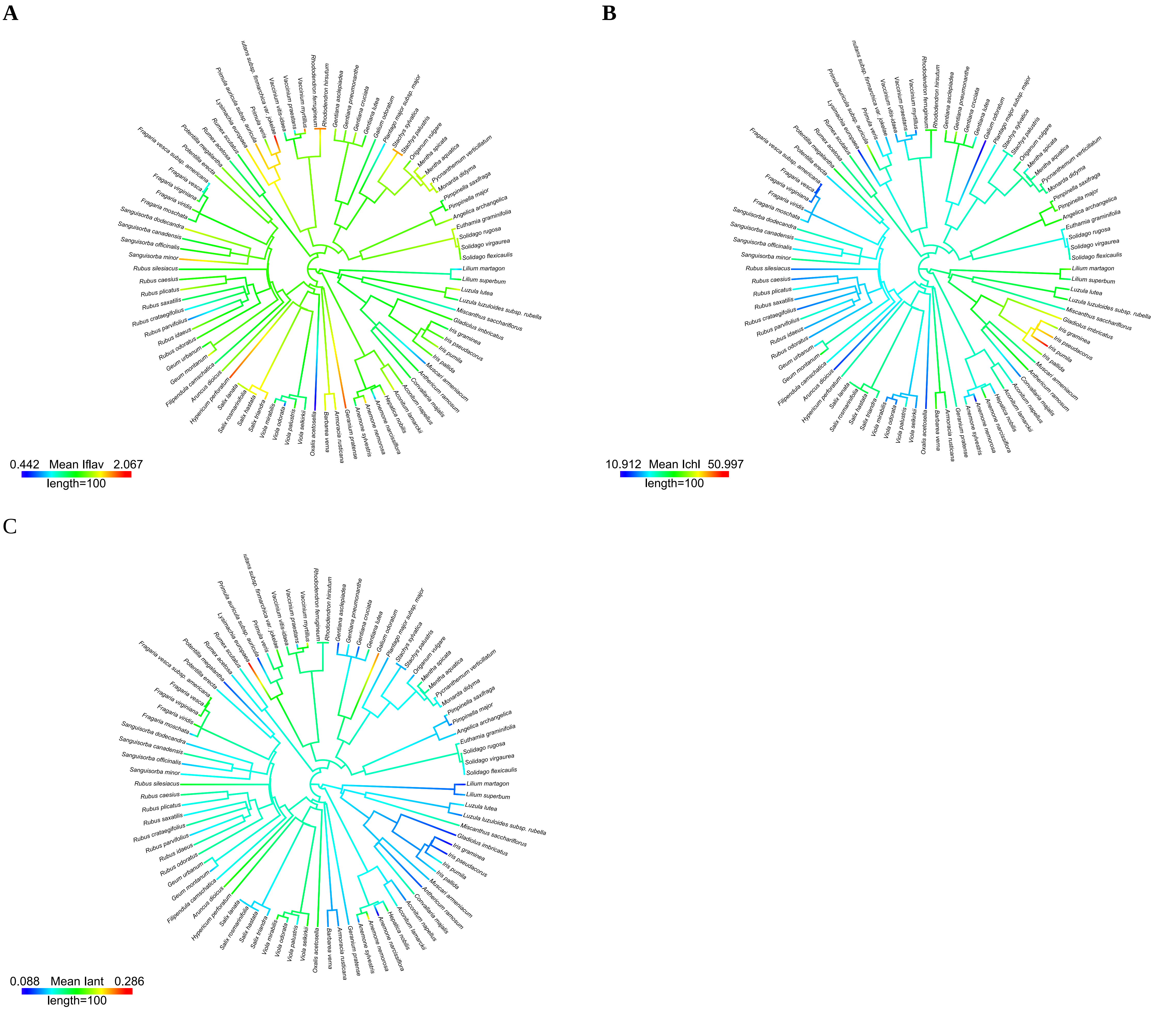
A
Rubus silesiacus
Rubus caesius
Rubus plicatus
Rubus saxatilis
Rubus crataegifolius
Rubus parvifolius
Rubus idaeus
Rubus odoratus
Geum urbanum
Geum montanum
Filipendula camschatica
Aruncus dioicus
Hypericum perforatum
Salix lanata
Salix rosmarinifolia
Salix hastata
Salix triandra
Viola mirabilis
Viola odorata
Viola palustris
Viola selkirkii
Oxalis acetosella
Barbarea verna
Armoracia rusticana
Geranium pratense
Anemone sylvestris
Anemone nemorosa
Anemone narcissiflora
Hepatica nobilis
Aconitum lamarckii
Aconitum napellus
Convallaria majalis
Anthericum ramosum
Muscari armeniacum
Iris pallida
Iris pumila
Iris pseudacorus
Iris graminea
Gladiolus imbricatus
Miscanthus sacchariflorus
Luzula luzuloides subsp. rubella
Luzula lutea
Lilium superbum
Lilium martagon
Solidago flexicaulis
Solidago virgaurea
Solidago rugosa
Euthamia graminifolia
Angelica archangelica
Pimpinella major
Pimpinella saxifraga
Monarda didyma
Pycnanthemum verticillatum
Mentha aquatica
Mentha spicata
Origanum vulgare
Stachys palustris
Stachys sylvatica
Plantago major subsp. major
Galium odoratum
Gentiana lutea
Gentiana cruciata
Gentiana pneumonanthe
Gentiana asclepiadea
Rhododendron hirsutum
Rhododendron ferrugineum
Vaccinium myrtillus
Vaccinium praestans
Vaccinium vitis-idaea
iutans subsp. finmarchica var. jokelae
Primula veris
Primula auricula subsp. auricula
Lysimachia europaea
Rumex scutatus
Rumex acetosa
Potentilla megalantha
Potentilla erecta
Fragaria vesca subsp. americana
Fragaria vesca
Fragaria virginiana
Fragaria viridis
Fragaria moschata
Sanguisorba dodecandra
Sanguisorba canadensis
Sanguisorba officinalis
Sanguisorba minor
0.442 Mean Iflav 2.067
length=100
B
10.912 Mean Ichl 50.997
length=100
C
0.088 Mean Iant 0.286
length=100

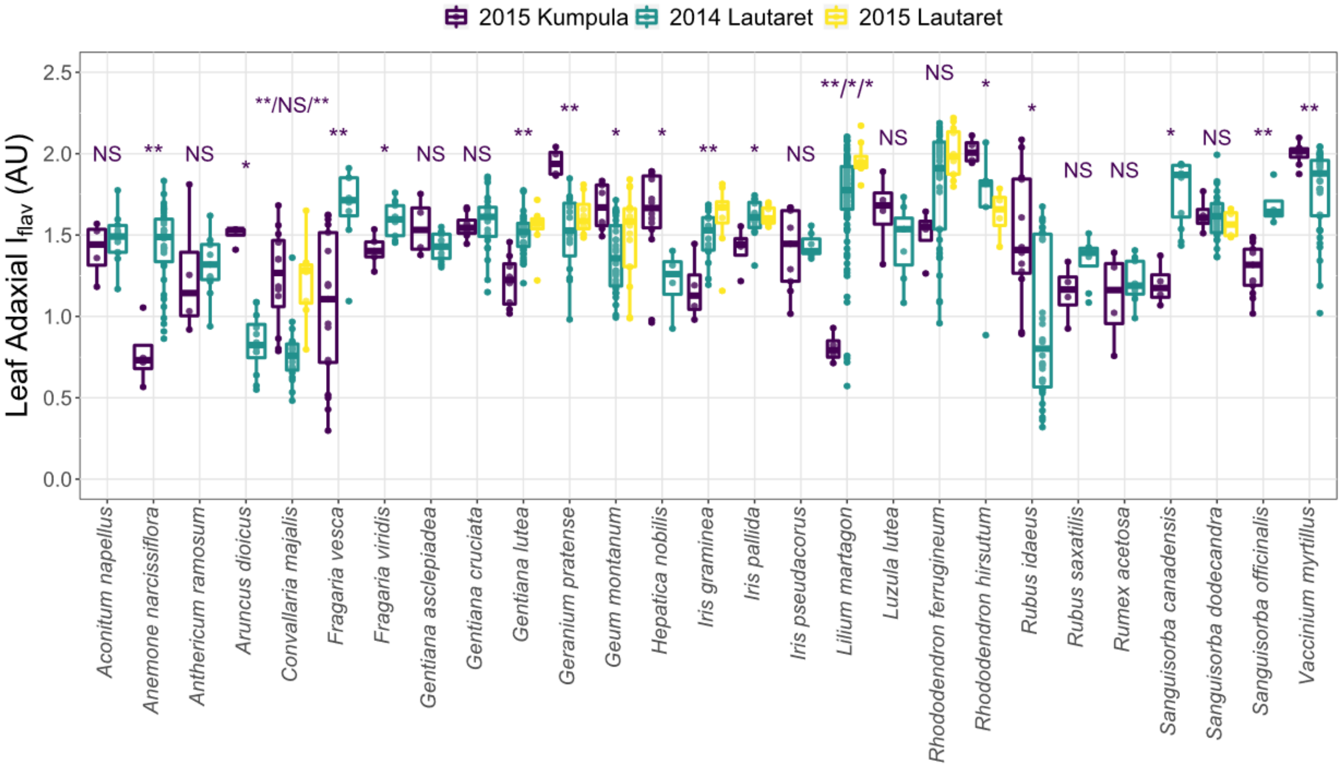

Figure S16. Leaf adaxial flavonol index (I_{flav}, Arbitrary Unit) measured from 27 species common to the high-elevation alpine botanical garden (Col du Lautaret, France) in two summers 2014 and 2015, and to the high latitude Kumpula Botanical Garden (Helsinki, Finland) in summer 2015. When no differences were found between two consecutive years of measurements from the alpine botanical garden, the samples were pooled and compared against those measured in Kumpula Botanical Garden. Interannual measurements are shown as separate boxplots, but significance level is indicated for the comparison of the two botanical gardens, except where there was a significant difference between years. For non-pooled samples, *Convallaria majalis* and *Lilium martagon*, significance levels are shown between I_{flav} measurements from Finland and France in 2014; Finland and France in 2015; and interannual differences from France. For non-pooled samples, non-parametric Kruskal-Wallis test was used, in combination with Wilcoxon rank sum test. For pooled samples, a Student's or Welch *t*-test for homo- or heterogenous variances respectively was used, or a non-parametric Wilcoxon rank sum test for non-normally distributed data. The *p*- values were adjusted using Benjamini and Hochberg (1995) correction method. Significant differences are indicated as: * <0.05, **≤0.01, ***≤0.001, ****≤0.0001.

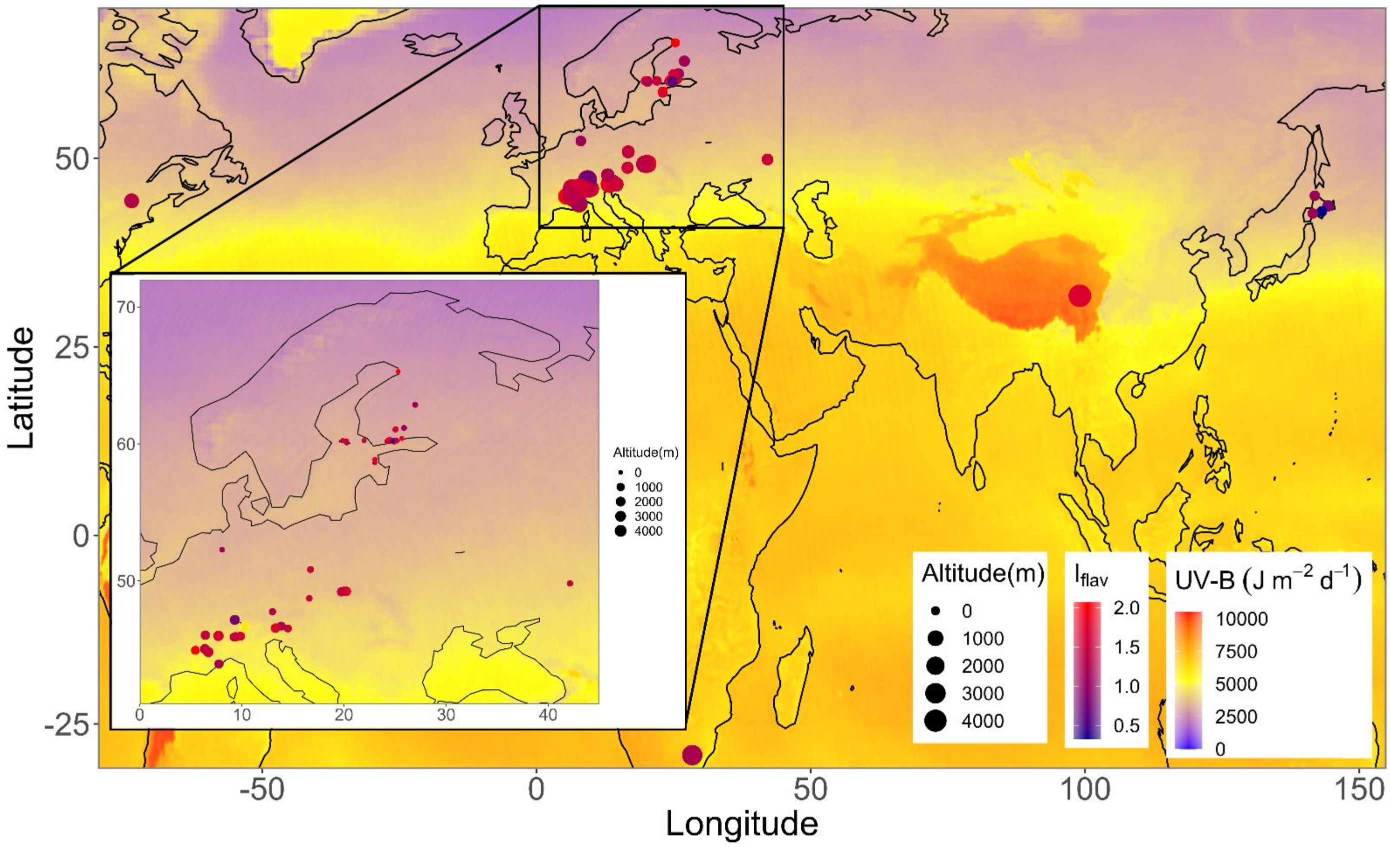

Figure S17. Mean optically measured flavonol/flavone index (I_{flav}, arbitrary unit, colour of the points) of studied plant taxa mapped according to their original collection sites. Plants were growing and sampled in alpine botanical garden (Col du Lautaret, France, *n* = 7) or Kumpula Botanical Garden (Helsinki, Finland, *n* = 51). The map colouration indicates mean UV-B radiation of the highest month (J m^{-2} d^{-1}) with resolution of 15 arc minutes (Beckmann et al., 2014). The size of the points indicate altitude (m a.s.l.) of original collection site of the plants. The mean of several taxa originating from the same location was used for nine collection sites.

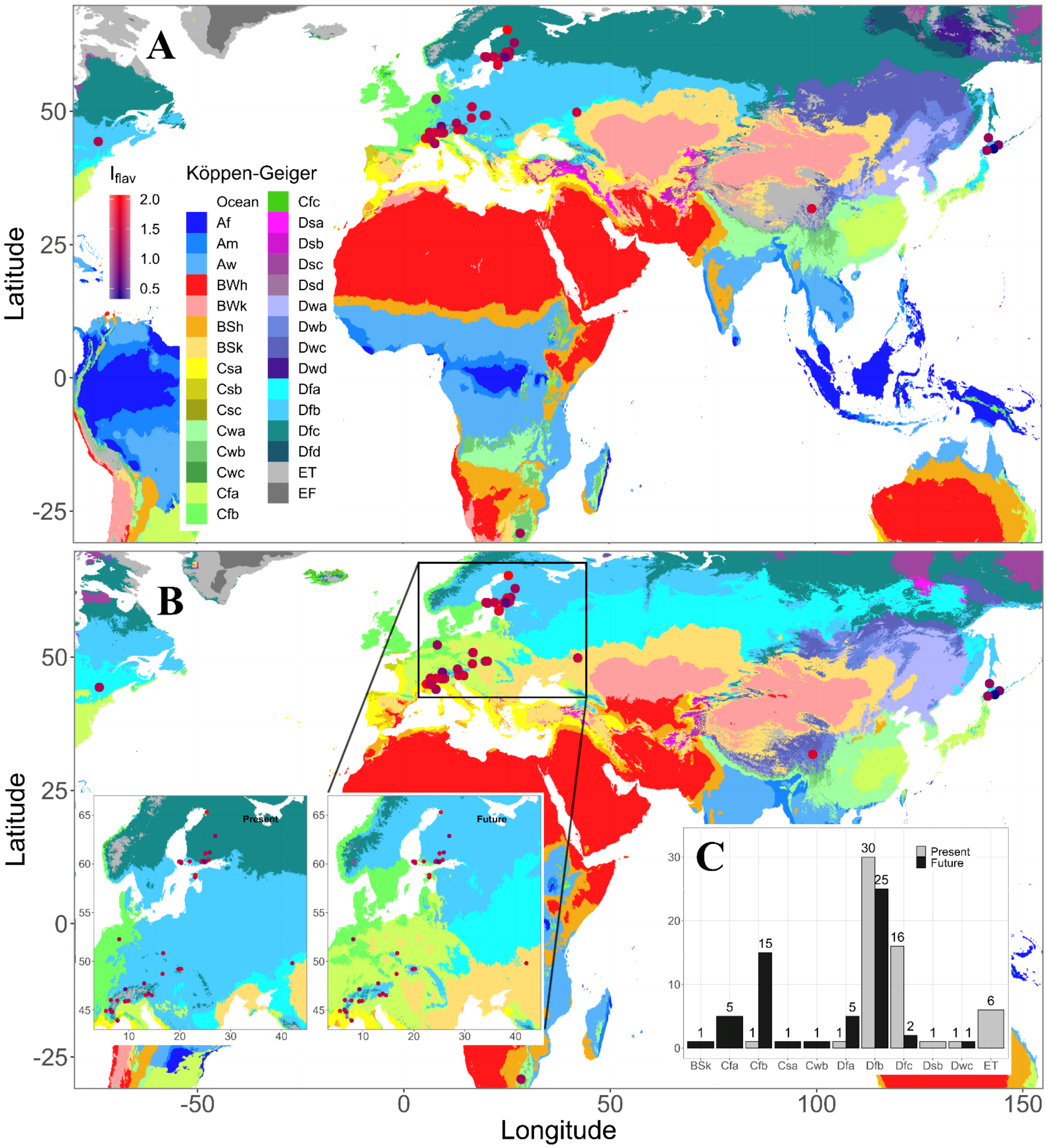

Figure S18. Original collection sites of the studied taxa shown on a map with Köppen-Geiger climate classified areas at present (**A**, 1980–2016) and projected for future (**B**, 2071–2100) (Beck et al., 2018). Optically measured mean flavonol/flavone index (I_{flav}, arbitrary unit) is indicated by the colour of the points. Panel **C** shows the number of sites in each class in the present day (grey bars) and in the future (black bars) (two datapoints located in a small island are excluded due to misclassification as ocean). Köppen-Geiger climate classes: A = Tropical climates, B = Dry climates, C = Temperate climates, D = Cold climates, E = Polar climates, further details may be found from Beck et al. (2018). Colour scheme was adopted from Beck et al. (2018).

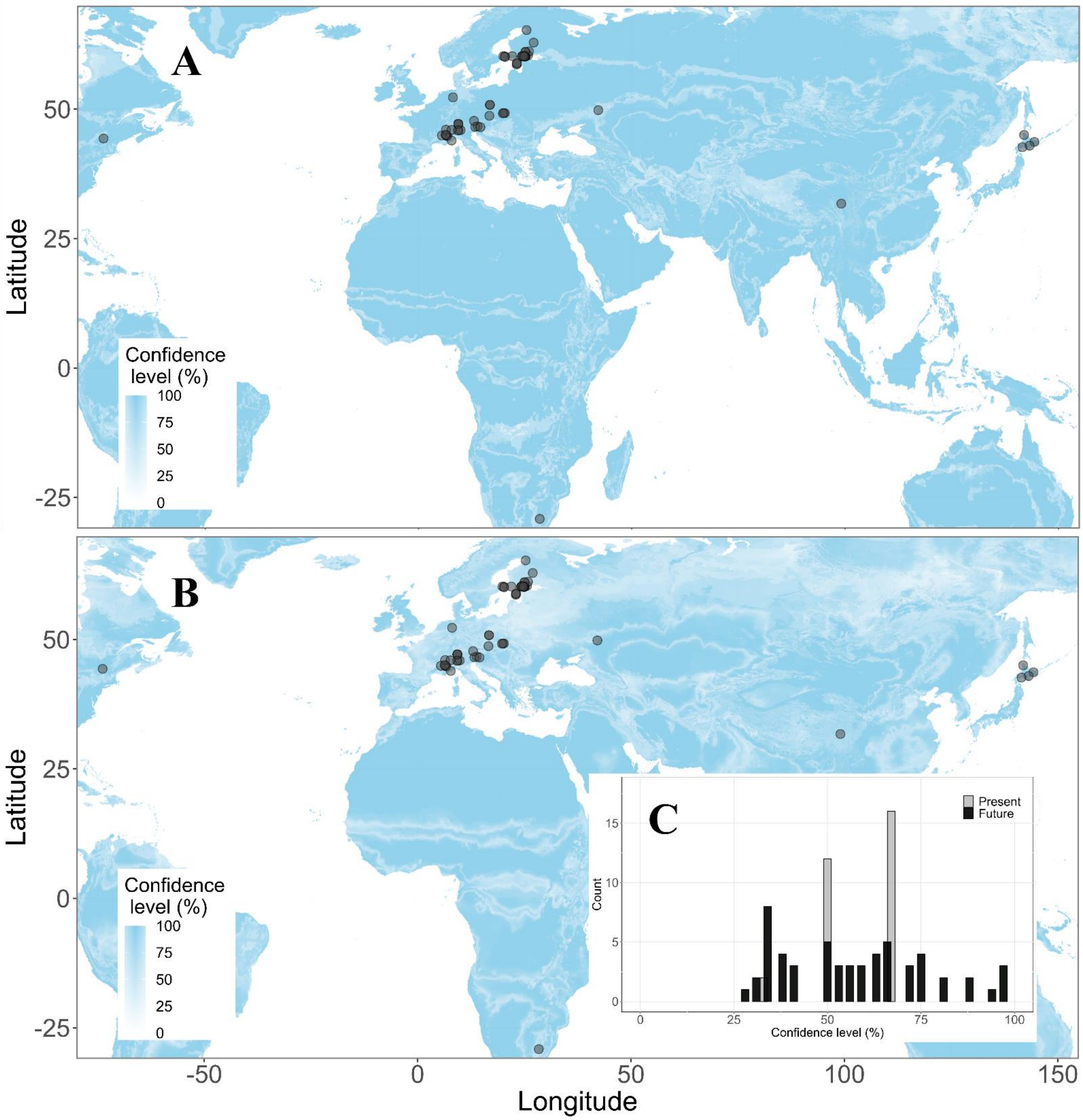

Figure S19. Confidence level (%) for Köppen-Geiger climate classification for the present day (**A**, 1980–2016) and projected future (**B**, 2071–2100) (Beck et al., 2018). Panel **C** shows confidence levels at the original collection sites of the studied plants (points) for present day (grey bars) and future projection (black bars).

Tables (S1-S6)

Table S1. Details of the parameters used by the optical leaf-clip, Dualex Scientific+ to measure adaxial flavonol/flavone (I_{flav}) and anthocyanin indices (I_{ant}), and leaf chlorophyll index (I_{chl}).

	I_{flav}	I_{chl}	I_{ant}
Range of index values	0-3	0-150	0-3
Excitation beam, nm	375	710	515
Reference beam, nm	635	850	635
Calculation	log of NIR fluorescence excited by red/ NIR fluorescence excited by UV-A	NIR transmittance - red transmittance / red transmittance	log of NIR fluorescence excited by red/ NIR fluorescence excited by green
Detection photodiode range, nm		710-900	

Table S2. Identification of sampled plants

Level of identification	*n*
Family	1
Genus	42†
Species	572 (28 + 6‡)
Subspecies	42
Variety	8
Hybrids	4
Seedlings or other§	3
Total	672

† 5/42 genera did not include measurement of species or other level of identification, ‡28 subspecies & 6 variety of different parental species § in text referred to as developmental stage e.g. taxa measured as seedling

Table S3. Taxa with highest and lowest mean adaxial flavonol/flavone index (I_{flav}) sampled from the alpine botanical garden in 2014 and the Kumpula Botanical Garden in 2015.

Site	Taxa with mean I_{flav} > 1.8 AU	Mean (± SE) I_{flav}	Taxa with mean I_{flav} < 1.0 AU	Mean (± SE) I_{flav}
Alpine botanical garden, France	*Cephalaria calcarea*	1.81 ± 0.02	*Adoxa moschatellina*	0.34 ± 0.03
	Betula seedling	1.81 ± 0.02	*Maianthemum*	0.37 ± 0.02
	Rhododendron ferrugineum	1.81 ± 0.05	*Sedum cauticola*	0.61 ± 0.05
	Primula grandis	1.81 ± 0.04	*Papaver victoris*	0.69 ± 0.06
	Gentiana alba	1.82 ± 0.04	*Hemerocallis lilioasphodelus*	0.69 ± 0.12
	Sorbus tree	1.82 ± 0.03	*Sedum anacampseros*	0.70 ± 0.05
	Iris clarkei	1.82 ± 0.04	*Geranium phaeum*	0.76 ± 0.05
	Cirsium heterophyllum	1.82 ± 0.06	*Convallaria majalis*	0.77 ± 0.04
	Gentiana dschungarica	1.82 ± 0.02	*Achillea clavennae*	0.77 ± 0.06
	Veronica gentianoides	1.82 ± 0.02	*Hieracium alpinum*	0.80 ± 0.11
	Cotoneaster juranus	1.82 ± 0.04	*Dactylis glomerata*	0.82 ± 0.05
	Prunus padus	1.82 ± 0.02	*Aruncus dioicus*	0.83 ± 0.05
	Populus tremula	1.83 ± 0.03	*Astragalus sikkimensis*	0.84 ± 0.07
	Alnus alnobetula	1.83 ± 0.02	*Festuca nigrescens*	0.85 ± 0.14
	Rodgersia podophylla	1.83 ± 0.08	*Cerastium candidissimum*	0.89 ± 0.04
	Alchemilla glomerulans	1.83 ± 0.05	*Aquilegia atrata*	0.89 ± 0.05
	Psephellus bellus	1.84 ± 0.05	*Symphytum caucasicum*	0.89 ± 0.04
	Alchemilla atropurpurea	1.84 ± 0.05	*Primula clusiana*	0.93 ± 0.06
	Primula x forsteri	1.84 ± 0.05	*Saxifraga hieraciifolia*	0.94 ± 0.06
	Vaccinium uliginosum	1.84 ± 0.03	*Primula polyneura*	0.95 ± 0.07
	Trifolium	1.84 ± 0.02	*Rubus idaeus*	0.96 ± 0.08
	Berberis vulgaris	1.84 ± 0.02	*Sedum spectabile*	0.98 ± 0.05
	Rhaponticum heleniifolium subsp. bicknellii	1.84 ± 0.02	*Hacquetia epipactis*	0.98 ± 0.14
	Nardostachys jatamansi	1.84 ± 0.03	*Podospermum roseum*	0.98 ± 0.07
	Fritillaria michailovskyi	1.84 ± 0.08	*Deschampsia cespitosa*	0.99 ± 0.02
	Knautia macedonica	1.85 ± 0.01		
	Populus seedling	1.85 ± 0.03		
	Rhamnus pumila	1.85 ± 0.10		
	Saussurea riederi	1.85 ± 0.03		
	Lilium carniolicum	1.85 ± 0.05		
	Syringa josikaea	1.86 ± 0.01		
	Dracocephalum grandiflorum	1.86 ± 0.01		
	Populus tremula seedling	1.87 ± 0.02		
	Ligularia macrophylla	1.87 ± 0.03		
	Lilium pyrenaicum	1.87 ± 0.03		
	Chenopodium bonus-henricus	1.88 ± 0.03		
	Gentiana occidentalis	1.88 ± 0.05		
	Anemone rupicola	1.88 ± 0.02		
	Alchemilla albinervia	1.89 ± 0.03		
	Rheum wittrockii	1.89 ± 0.02		
	Orthilia secunda	1.89 ± 0.15		
	Sibiraea altaiensis var. altaiensis	1.89 ± 0.01		
	Rhaponticum centauroides	1.89 ± 0.02		
	Dryas drummondii	1.89 ± 0.08		
	Leontopodium kamtschaticum	1.89 ± 0.02		
	Tussilago farfara	1.89 ± 0.04		
	Spiraea betulifolia	1.90 ± 0.06		
	Arabis soyeri subsp. subcoriacea	1.90 ± 0.08		
	Sorbus sitchensis	1.90 ± 0.03		
	Anaphalis royleana var. concolor	1.92 ± 0.09		
	Peucedanum ostruthium	1.92 ± 0.02		
	Potentilla speciosa	1.92 ± 0.01		
	Persicaria alpina	1.92 ± 0.02		
	Arnica chamissonis	1.92 ± 0.03		
	Geranium renardii	1.93 ± 0.01		
	Celmisia monroi	1.98 ± 0.01		
	Tephroseris integrifolia	1.98 ± 0.03		
	Alchemilla gorcensis	1.98 ± 0.02		
	Cortusa sibirica	1.99 ± 0.03		
	Primula warshenewskiana	2.00 ± 0.03		
	Fritillaria pallidiflora	2.00 ± 0.03		
	Buphthalmum salicifolium	2.04 ± 0.07		
	Penstemon ellipticus	2.06 ± 0.04		
	Rhaponticum carthamoides	2.07 ± 0.04		
	Penstemon fruticosus	2.09 ± 0.04		
	Veronica lutea	2.09 ± 0.04		

Site	Taxa with mean I_{flav} > 1.8 AU	Mean (± SE) I_{flav}	Taxa with mean I_{flav} < 1.0 AU	Mean (± SE) I_{flav}
	Scutellaria alpina	2.12 ± 0.04		
Kumpula Botanical Garden, Finland	*Sanguisorba minor*	1.81 ± 0.06	*Oxalis acetosella*	0.44 ± 0.05
	Pycnanthemum verticillatum	1.81 ± 0.02	*Viola odorata*	0.60 ± 0.07
	Solidago virgaurea	1.82 ± 0.07	*Rubus parvifolius*	0.72 ± 0.17
	Salix hastata	1.85 ± 0.02	*Muscari armeniacum*	0.73 ± 0.05
	Stachys palustris	1.92 ± 0.07	*Anemone narcissiflora*	0.77 ± 0.10
	Geranium pratense	1.95 ± 0.04	*Vaccinium praestans*	0.80 ± 0.07
	Hypericum perforatum	1.96 ± 0.02	*Lilium martagon*	0.81 ± 0.05
	Vaccinium myrtillus	2.00 ± 0.02	*Fragaria vesca subsp. americana*	0.86 ± 0.16
	Rhododendron hirsutum	2.02 ± 0.04	*Plantago major subsp. major*	0.87 ± 0.06
	Primula nutans subsp. finmarchica var. jokelae	2.07 ± 0.03	*Rubus crataegifolius*	0.88 ± 0.07
			Miscanthus sacchariflorus	0.99 ± 0.21

Table S4. Mean (± SE) of diurnal PSII photochemistry and optically measured I_{flav} (arbitrary unit) from taxa measured in the alpine botanical garden (Col du Lautaret, France). Non-photochemical quenching (NPQ) was calculated according to Murchie and Lawson (2013). The parameters describing the fraction of energy dissipated either passively in form of heat and fluorescence, Y(NO), or dissipated in form of heat through the regulated photoprotective NPQ mechanism, Y(NPQ), were calculated according to Klughammer and Schreiber (2008).

Optical measurement	67 taxa with no difference in diurnal F_v/F_m†	19‡ taxa with differences in diurnal F_v/F_m†	All 86‡ taxa
Number of measurements in total (≥ 4 per taxa)	312	116	428
F_v/F_m predawn	0.80 ± 0.003	0.82 ± 0.003	0.81 ± 0.003
F_v/F_m midday	0.77 ± 0.004	0.77 ± 0.004	0.77 ± 0.003
NPQ (predawn F_m & mid-morning F_m')	4.25 ± 0.153	4.46 ± 0.264	4.30 ± 0.133
NPQ (predawn F_m & midday F_m')	3.57 ± 0.143	4.28 ± 0.221	3.76 ± 0.121
NPQ (midday F_m & midday F_m')	3.08 ± 0.124	3.20 ± 0.157	3.11 ± 0.100
Y(NO) (predawn F_m & mid-morning F')	0.14 ± 0.005	0.13 ± 0.005	0.14 ± 0.004
Y(NO) (predawn F_m & midday F')	0.14 ± 0.005	0.11 ± 0.004	0.13 ± 0.004
Y(NPQ) (predawn F_m & mid-morning F', F_m')	0.45 ± 0.011	0.46 ± 0.018	0.46 ± 0.009
Y(NPQ) (predawn F_m & midday F', F_m')	0.39 ± 0.011	0.43 ± 0.016	0.40 ± 0.009
I_{flav}	1.60 ± 0.015	1.61 ± 0.018	1.60 ± 0.012

†Significant differences between diurnal measurements of F_v/F_m were tested by Student's/Welch *t*-test, or Wilcoxon test and *p*-values were adjusted using Benjamini and Hockhberg (1995) correction method. ‡ Including separate measurements from mature *Eryngium alpinum* and their seedlings.

Table S5. Relationship between optically measured plant mean leaf traits (I_{flav}, I_{chl} and I_{ant}) and common solar radiation and weather variables at plants' original collection site. Climate data was obtained from WorldClim (Bio 1-15, mean monthly solar radiation, Fick and Hijmans, 2017) and CliMond databases (Bio 20-27, Hutchinson et al., 2009, Kriticos et al., 2014). UV-B radiation data was acquired from a climatology Beckmann et al. (2014). The 95 % confidence intervals (CI) were computed by bootstrapping (R function spearman.ci from R package RVAideMemoire).

	I_{flav}, *r* (95% CI)	Sig./ adjusted sig.	I_{chl}, *r* (95% CI)	Sig./ adjusted sig.	I_{ant}, *r* (95% CI)	Sig./ adjusted sig.	Categorised light condition, *r* (95% CI)	Sig./ adjusted sig.
Latitude			-0.38<br>(-0.58, -0.21)	**/*				
Longitude								
Elevation, m a.s.l.			0.52<br>(0.31, 0.67)	****/**	-0.37<br>(-0.66, -0.21)	**/*		
Annual Mean UV-B, J m^{-2} day^{-1}			0.51<br>(0.34, 0.68)	****/**				
Annual UV-B Seasonality, J m^{-2} day^{-1}			0.54<br>(0.38, 0.70)	****/***				
Mean UV-B of the highest Month, J m^{-2} day^{-1}			0.52<br>(0.36, 0.69)	****/**				
Mean UV-B of the lowest Month, J m^{-2} day^{-1}			0.44<br>(0.27, 0.64)	***/**				
Monthly mean sum of the highest quarter UV-B, J m^{-2} day^{-1}			0.52<br>(0.34, 0.70)	****/**				
Monthly mean sum of the lowest quarter UV-B, J m^{-2} day^{-1}			0.47<br>(0.30, 0.65)	***/**				
Solar radiation January, kJ m^{-2} day^{-1}			0.43<br>(0.22, 0.60)	**/**				
Solar radiation February, kJ m^{-2} day^{-1}			0.46<br>(0.26, 0.64)	***/**	-0.27<br>(-0.49, -0.06)	*/NS		
Solar radiation March, kJ m^{-2} day^{-1}			0.45<br>(0.25, 0.62)	***/**				
Solar radiation April, kJ m^{-2} day^{-1}			0.44<br>(0.25, 0.61)	***/**				
Solar radiation May, kJ m^{-2} day^{-1}	0.30<br>(0.11, 0.56)	*/NS					0.60†<br>(-0.10, 0.46)	*/NS
Solar radiation June, kJ m^{-2} day^{-1}	0.31<br>(0.06, 0.54)	*/NS						
Solar radiation July, kJ m^{-2} day^{-1}	0.31<br>(0.06, 0.54)	*/NS						
Solar radiation August, kJ m^{-2} day^{-1}	0.28<br>(-0.01, 0.51)	*/NS	0.44<br>(0.20, 0.62)	***/**				
Solar radiation September, kJ m^{-2} day^{-1}			0.49<br>(0.28, 0.65)	***/**				
Solar radiation October, kJ m^{-2} day^{-1}			0.46<br>(0.25, 0.62)	***/**				
Solar radiation November, kJ m^{-2} day^{-1}			0.44<br>(0.26, 0.61)	***/**				
Solar radiation December, kJ m^{-2} day^{-1}			0.44<br>(0.23, 0.63)	***/**				
Mean UV-B January, J m^{-2} day^{-1}			0.44<br>(0.26, 0.63)	***/**				
Mean UV-B February, J m^{-2} day^{-1}			0.46<br>(0.26, 0.64)	***/**				
Mean UV-B March, J m^{-2} day^{-1}			0.45<br>(0.21, 0.63)	***/**				
Mean UV-B April, J m^{-2} day^{-1}			0.50<br>(0.30, 0.69)	***/**				
Mean UV-B May, J m^{-2} day^{-1}			0.46<br>(0.26, 0.68)	***/**				
Mean UV-B June, J m^{-2} day^{-1}			0.45<br>(0.25, 0.67)	***/**			0.56†<br>(0.05, 0.52)	*/NS
Mean UV-B July, J m^{-2} day^{-1}			0.45<br>(0.25, 0.65)	***/**				
Mean UV-B August, J m^{-2} day^{-1}			0.49<br>(0.32, 0.66)	***/**				

	I_{flav}, *r* (95% CI)	Sig./ adjusted sig.	I_{chl}, *r* (95% CI)	Sig./ adjusted sig.	I_{ant}, *r* (95% CI)	Sig./ adjusted sig.	Categorised light condition, *r* (95% CI)	Sig./ adjusted sig.
Mean UV-B September, J m^{-2} day^{-1}			0.52 (0.37, 0.68)	****/**				
Mean UV-B October, J m^{-2} day^{-1}			0.48 (0.31, 0.64)	***/**				
Mean UV-B November, J m^{-2} day^{-1}			0.44 (0.28, 0.64)	***/**				
Mean UV-B December, J m^{-2} day^{-1}			0.44 (0.26, 0.63)	***/**				
Mean annual radiation (Bio20), W m^{-2}			0.58 (0.38, 0.72)	****/***	-0.28 (-0.54, -0.07)	*/NS		
Highest weekly radiation (Bio21), W m^{-2}								
Lowest weekly radiation (Bio22), W m^{-2}			0.42 (0.21, 0.60)	**/**				
Radiation seasonality (Bio23), W m^{-2}			-0.36 (-0.55, -0.15)	**/*				
Radiation of wettest quarter (Bio24), W m^{-2}								
Radiation of driest quarter (Bio25), W m^{-2}					0.28 (-0.04, 0.55)	*/NS		
Radiation of warmest quarter (Bio26), W m^{-2}	0.32 (0.01, 0.52)	*/NS	0.55 (0.35, 0.71)	****/***				
Radiation of coldest quarter (Bio27), W m^{-2}			0.40 (0.19, 0.57)	**/*				
Annual mean temperature (Bio1), °C								
Temperature seasonality (Bio4), °C (sd * 100)			-0.40 (-0.61, -0.14)	**/*				
Maximum temperature of the warmest month (Bio5), °C	-0.28 (-0.45, 0.02)	*/NS						
Minimum temperature of the coldest month (Bio6), °C								
Annual precipitation (Bio12), mm			0.34 (0.07, 0.56)	*/*				
Precipitation seasonality (Bio15), coefficient of variation								

n = 58. Significant differences are indicated as: *<0.05, **≤0.01, ***≤0.001, ****≤0.0001 while NS are left completely empty for visual clarity. Benjamini and Hochberg (1995) method was used to adjust *p* -values. † Bootstrap function produced differing results where *r* = 0.17 (solar radiation May) and *r* = 0.29 (mean UV-B June).

Table S6. Taxa showing significant results ($p < 0.05$) from testing the local indicator of phylogenetic association (local Moran's *I*) for optically measured mean leaf traits (I_{flav}, I_{chl}, I_{ant}, all in arbitrary units). The phylogenies were generated using a published mega-tree and a tool by Jin and Qian (2019). The 622 taxa growing in the alpine botanical garden (Col du Lautaret, France) and 86 taxa growing in Kumpula Botanical Garden (Helsinki, Finland) were used separately in phylogenies for the analysis.

	Species with significant local Moran's *I* (I_{flav})	Local Moran's *I* for I_{flav}	Mean I_{flav}	Difference to mean I_{flav}		Species with significant local Moran's *I* (I_{chl})	Local Moran's *I* for I_{chl}		Species with significant local Moran's *I* (I_{ant})	Local Moran's *I* for I_{ant}
1	*Adoxa moschatellina*	-0.05	0.34	-1.19	1	*Acer pseudoplatanus*	0.02	1	*Acaena microphylla*	0.02
2	*Alchemilla albinervia**	0.29	1.89	0.35	2	*Achillea clavennae*	0.18	2	*Acer pseudoplatanus*	0.01
3	*Alchemilla alpigena*	0.18	1.72	0.19	3	*Achillea lingulata*	0.15	3	*Adenostyles alpina*	0.12
4	*Alchemilla atropurpurea*	0.26	1.84	0.31	4	*Aconitum firmum*	0.59	4	*Adenostyles leucophylla*	0.12
5	*Alchemilla conjuncta*	-0.18	1.35	-0.19	5	*Aconitum tanguticum*	0.27	5	*Ajuga pyramidalis*	0.13
6	*Alchemilla glomerulans*	0.26	1.83	0.30	6	*Actaea spicata*	0.06	6	*Allium brevistylum*	0.08
7	*Alchemilla gorcensis*	0.35	1.98	0.45	7	*Allium brevistylum*	0.26	7	*Allium insubricum*	0.10
8	*Alchemilla mollis*	0.20	1.74	0.21	8	*Allium insubricum*	0.47	8	*Allium lusitanicum*	0.41
9	*Alchemilla polatschekiana*	0.18	1.72	0.19	9	*Allium lusitanicum*	0.68	9	*Allium nutans*	0.41
10	*Alchemilla saxatilis*	0.23	1.79	0.26	10	*Allium moly*	0.28	10	*Allium pyrenaicum*	0.07
11	*Alchemilla xanthochlora*	0.23	1.78	0.25	11	*Allium nutans*	0.90	11	*Alnus alnobetula*	0.02
12	*Aruncus dioicus*	-0.11	0.83	-0.70	12	*Allium pyrenaicum*	0.27	12	*Alnus alnobetula subsp. sinuata*	0.04
13	*Astragalus sikkimensis*	0.10	0.84	-0.69	13	*Allium victorialis*	0.09	13	*Anthericum ramosum*	0.02
14	*Cerastium banaticum**	0.21	1.18	-0.35	14	*Alnus alnobetula subsp. sinuata*	0.03	14	*Aquilegia canadensis*	0.39
15	*Cerastium biebersteinii**	0.22	1.18	-0.35	15	*Anemone halleri*	0.09	15	*Aquilegia chrysantha*	0.36
16	*Cerastium candidissimum*	0.34	0.89	-0.64	16	*Anemone pavoniana*	0.10	16	*Aquilegia elegantula*	-0.44
17	*Cerastium carinthiacum subsp. austroalpinum**	0.18	1.25	-0.29	17	*Anthericum ramosum*	0.04	17	*Campanula rapunculoides*	-0.26
18	*Cerastium eriophorum*	0.22	1.16	-0.37	18	*Aquilegia chrysantha*	0.48	18	*Carduus kerneri*	0.09
19	*Cerastium tomentosum*	0.28	1.05	-0.48	19	*Aquilegia desertorum*	0.48	19	*Centaurea fischeri*	0.15
20	*Chenopodium bonus-henricus**	-0.02	1.88	0.35	20	*Aquilegia einseleana*	-0.80	20	*Convolvulus suendermannii*	-0.01
21	*Convallaria majalis*	0.05	0.77	-0.76	21	*Aruncus dioicus*	0.06	21	*Cymbalaria hepaticifolia*	-0.05
22	*Cynoglossum magellense**	0.04	1.21	-0.32	22	*Aster flaccidus*	0.15	22	*Epimedium alpinum*	0.01
23	*Dactylis glomerata*	0.30	0.82	-0.71	23	*Aster tongolensis*	0.15	23	*Festuca paniculata*	0.07
24	*Deschampsia cespitosa*	0.25	0.99	-0.54	24	*Berberis vulgaris*	0.03	24	*Geranium argenteum*	0.27
25	*Festuca nigrescens*	0.34	0.85	-0.68	25	*Celmisia monroi*	0.31	25	*Geranium cinereum*	0.23
26	*Festuca paniculata*	0.23	1.17	-0.36	26	*Cerastium eriophorum*	-0.18	26	*Geranium subcaulescens*	0.24
27	*Fritillaria michailovskyi*	0.13	1.84	0.31	27	*Cicerbita thianschanica*	-0.07	27	*Gerbera maxima*	0.05
28	*Fritillaria pallidiflora**	0.16	2.00	0.47	28	*Clematis alpina*	0.09	28	*Heuchera micrantha var. erubescens*	0.03
29	*Geranium phaeum*	-0.09	0.76	-0.77	29	*Cyanus triumfettii subsp. axillaris*	0.21	29	*Hieracium hyparcticum*	0.17
30	*Hacquetia epipactis*	-0.04	0.98	-0.55	30	*Cymbalaria hepaticifolia*	-0.04	30	*Hypochaeris uniflora*	0.05
31	*Hieracium alpinum*	1.32	0.80	-0.73	31	*Dictamnus albus*	0.01	31	*Iris clarkei*	0.07
32	*Hieracium amplexicaule**	0.40	1.33	-0.20	32	*Doronicum corsicum*	-0.18	32	*Iris forrestii*	0.10
33	*Hieracium erioleucum**	0.31	1.18	-0.35	33	*Epimedium alpinum*	0.06	33	*Iris hookeri*	0.13
34	*Hieracium pannosum subsp. bornmuelleri**	0.26	1.23	-0.30	34	*Euphorbia dulcis*	0.14	34	*Iris orientalis*	0.07
35	*Hieracium prenanthoides*	0.48	1.29	-0.25	35	*Euphorbia hyberna*	0.15	35	*Iris pseudacorus*	0.14
36	*Hieracium tomentosum**	1.06	1.13	-0.40	36	*Gentiana angustifolia*	-0.12	36	*Iris setosa*	0.17
37	*Lilium carniolicum*	0.19	1.85	0.32	37	*Gentiana clusii*	0.15	37	*Iris sibirica*	0.11
38	*Lilium pyrenaicum*	0.20	1.87	0.34	38	*Gentiana pannonica*	0.19	38	*Iris versicolor*	0.14
39	*Luzula nivea*	0.08	1.06	-0.47	39	*Gentiana parryi*	0.12	39	*Leptinella potentillina*	-0.09
40	*Meconopsis grandis*	0.05	1.25	-0.28	40	*Heuchera micrantha var. erubescens*	0.05	40	*Linum flavum*	0.02
41	*Ornithogalum pyrenaicum*	0.02	1.21	-0.32	41	*Hieracium erioleucum*	0.26	41	*Lonicera caerulea*	0.05
42	*Oxyria digyna**	-0.03	1.19	-0.34	42	*Hieracium hyparcticum*	0.45	42	*Lonicera caucasica subsp. caucasica*	0.10
43	*Papaver alpinum*	0.10	1.15	-0.38	43	*Hieracium pannosum subsp. bornmuelleri*	0.23	43	*Lonicera tatarica*	0.10
44	*Papaver victoris*	0.10	0.69	-0.84	44	*Hypochaeris uniflora*	0.07	44	*Narcissus poeticus*	0.15
45	*Penstemon ellipticus**	0.74	2.06	0.53	45	*Iris alberti*	0.49	45	*Narcissus pseudonarcissus*	0.16

46	*Penstemon fruticosus**	1.19	2.09	0.56
47	*Penstemon fruticosus var. scouleri*	0.79	1.71	0.18
48	*Penstemon newberryi*	0.33	1.70	0.17
49	*Potentilla collina**	-0.02	1.22	-0.31
50	*Primula polyneura*	-0.22	0.95	-0.58
51	*Primula warshenewskiana*	0.09	2.00	0.47
52	*Rhaponticum carthamoides**	0.28	2.07	0.54
53	*Rhaponticum centauroides**	0.94	1.89	0.36
54	*Rhaponticum heleniifolium subsp. bicknellii**	0.89	1.84	0.31
55	*Rhaponticum scariosum subsp. rhaponticum*	0.20	1.75	0.22
56	*Rubus idaeus*	-0.03	0.96	-0.57
57	*Scilla lilio-hyacinthus*	0.03	1.20	-0.33
58	*Sedum anacampseros*	0.65	0.70	-0.83
59	*Sedum cauticola*	0.65	0.61	-0.92
60	*Sedum spectabile*	0.05	0.98	-0.55
61	*Sesleria nitida*	0.15	1.25	-0.28
62	*Sorbus sitchensis**	0.07	1.90	0.37
63	*Stachys byzantina**	-0.15	1.12	-0.41
64	*Symphytum asperum*	0.18	1.07	-0.46
65	*Symphytum caucasicum**	0.19	0.89	-0.64
	Species with significant results without shaded plants			
1	*Arabis soyeri subsp. subcoriacea*	-0.36	1.90	0.37
2	*Aubrieta deltoidea*	0.08	1.12	-0.41
3	*Centaurea parlatoris subsp. nigra*	-0.26	1.16	-0.37
4	*Cicerbita thianschanica*	0.16	1.05	-0.48
5	*Cotoneaster juranus*	0.08	1.82	0.29
6	*Isatis tinctoria*	0.08	1.20	-0.33
7	*Leontopodium kamtschaticum*	-0.47	1.89	0.36
8	*Mertensia primuloides*	0.18	1.32	-0.21
9	*Pilosella aurantiaca*	0.18	1.43	-0.10
10	*Prunus padus*	0.03	1.82	0.29
11	*Pseudomertensia echioides*	0.19	1.28	-0.25
12	*Scutellaria alpina*	-0.09	2.12	0.59
13	*Sibiraea altaiensis var. altaiensis*	0.07	1.89	0.36
14	*Sibiraea laevigata*	0.05	1.77	0.24
15	*Spiraea betulifolia*	0.06	1.90	0.37
16	*Tanacetum praeteritum*	0.24	1.14	-0.39
	Species from Kumpula Botanical Garden			
1	*Geranium pratense*	-0.08	1.95	0.57
2	*Hypericum perforatum*	-0.10	1.96	0.58
3	*Sanguisorba minor*	-0.09	1.81	0.43
4	*Vaccinium myrtillus*	-0.94	2.00	0.62

46	*Iris aphylla*	0.61
47	*Iris clarkei*	0.15
48	*Iris graminea*	-0.10
49	*Iris hookeri*	0.75
50	*Iris lutescens*	0.59
51	*Iris missouriensis*	0.13
52	*Iris orientalis*	0.21
53	*Iris pallida*	0.10
54	*Iris perrieri*	-0.12
55	*Iris pseudacorus*	0.42
56	*Iris reichenbachii*	0.15
57	*Iris setosa*	0.54
58	*Iris sibirica*	0.31
59	*Iris versicolor*	0.64
60	*Lactuca alpina*	-0.09
61	*Lactuca macrophylla*	-0.09
62	*Leptinella potentillina*	-0.13
63	*Ligularia macrophylla*	0.13
64	*Linum flavum*	0.02
65	*Lonicera caerulea*	0.04
66	*Lonicera caucasica subsp. caucasica*	0.05
67	*Lonicera tatarica*	0.05
68	*Narcissus poeticus*	0.60
69	*Narcissus pseudonarcissus*	0.63
70	*Ornithogalum pyrenaicum*	-0.04
71	*Oxytropis jacquinii*	-0.09
72	*Podospermum roseum*	0.10
73	*Populus tremula*	0.05
74	*Potentilla diversifolia*	-0.07
75	*Potentilla speciosa*	-0.03
76	*Prunus padus*	0.05
77	*Psephellus transcaucasicus*	-0.43
78	*Ribes lacustre*	0.08
79	*Ribes nigrum*	0.06
80	*Ribes rubrum*	0.06
81	*Rodgersia podophylla*	0.07
82	*Rosa pendulina*	0.03
83	*Rubus idaeus*	0.03
84	*Rubus saxatilis*	0.03
85	*Rudbeckia montana*	0.04
86	*Sanguisorba albiflora*	0.03
87	*Sanguisorba dodecandra*	0.03
88	*Sedum cauticola*	-0.26
89	*Sedum spectabile*	0.02
90	*Spiraea betulifolia*	0.08
91	*Spiraea hypericifolia*	0.09
92	*Tanacetum macrophyllum*	-0.20
93	*Tephroseris integrifolia subsp. capitata*	0.44
94	*Waldsteinia fragarioides*	0.02
	Species from Kumpula Botanical Garden	
1	*Convallaria majalis*	-0.12
2	*Fragaria vesca subsp. americana*	1.12

46	*Paeonia coriacea*	0.16
47	*Paeonia delavayi*	0.25
48	*Paeonia lactiflora*	0.19
49	*Penstemon digitalis*	8.06
50	*Penstemon ellipticus*	0.49
51	*Penstemon fruticosus*	2.45
52	*Penstemon fruticosus var. scouleri*	1.96
53	*Penstemon newberryi*	4.35
54	*Penstemon peckii*	5.96
55	*Penstemon procerus*	1.78
56	*Penstemon rupicola*	2.09
57	*Penstemon serrulatus*	0.96
58	*Petasites paradoxus*	0.08
59	*Podospermum roseum*	0.04
60	*Populus tremula*	0.04
61	*Primula involucrata*	-0.11
62	*Prunus padus*	0.04
63	*Rhamnus pumila*	0.01
64	*Ribes lacustre*	0.08
65	*Ribes nigrum*	0.05
66	*Ribes rubrum*	0.08
67	*Ribes uva-crispa*	0.03
68	*Rodgersia podophylla*	0.06
69	*Rudbeckia laciniata*	0.02
70	*Rudbeckia montana*	0.04
71	*Salix pyrenaica*	0.11
72	*Scutellaria alpina*	-0.04
73	*Spiraea betulifolia*	0.08
74	*Spiraea hypericifolia*	0.10
75	*Syringa josikaea*	0.05
76	*Vaccinium myrtillus*	0.05
77	*Vaccinium uliginosum*	0.05
78	*Veronica urticifolia*	0.23
79	*Veronica wormskjoldii*	0.24
	Species with marginally significant/NS result	
1	*Allium moly*	0.05
2	*Orthilia secunda*	0.01
3	*Salvia przewalskii*	-0.05
	Species from Kumpula Botanical Garden	
1	*Anemone narcissiflora*	-0.35
2	*Anemone nemorosa*	-0.69
3	*Galium odoratum*	-0.15
4	*Gladiolus imbricatus*	0.08
5	*Iris graminea*	0.21
6	*Iris pseudacorus*	0.20
7	*Lysimachia europaea*	-0.11
8	*Primula auricula subsp. auricula*	-0.19

3	*Fragaria virginiana*	0.73
4	*Galium odoratum*	-0.08
5	*Gladiolus imbricatus*	0.24
6	*Iris graminea*	0.71
7	*Iris pallida*	0.55
8	*Iris pseudacorus*	0.96
9	*Iris pumila*	1.08
10	*Primula auricula subsp. auricula*	-0.20
11	*Rubus crataegifolius*	0.07

* Taxa which had significant local autocorrelation for the phylogeny excluding shaded plants (shown for I_{flav} only)

Figure 2
panel A

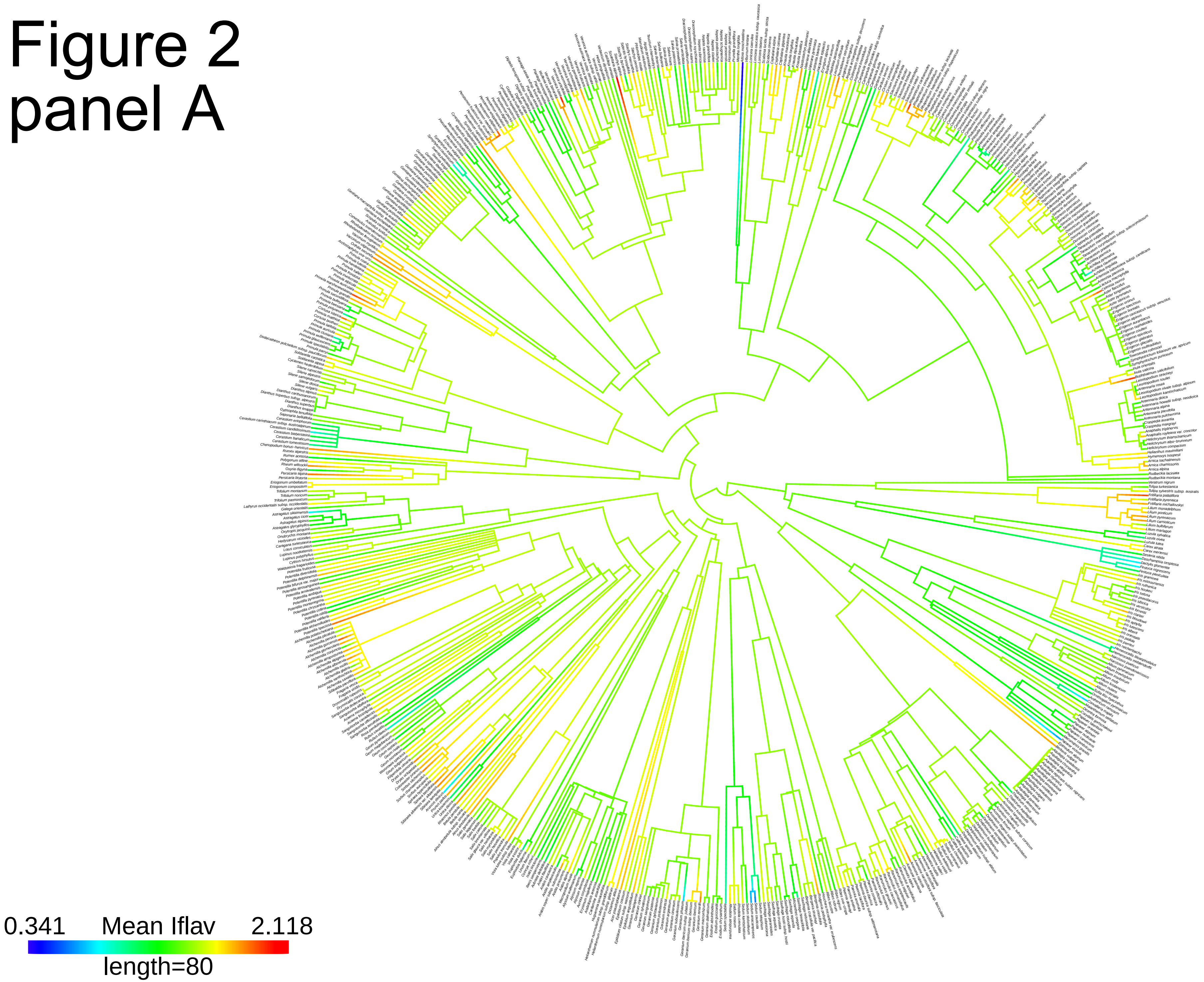

Figure 2
panel B

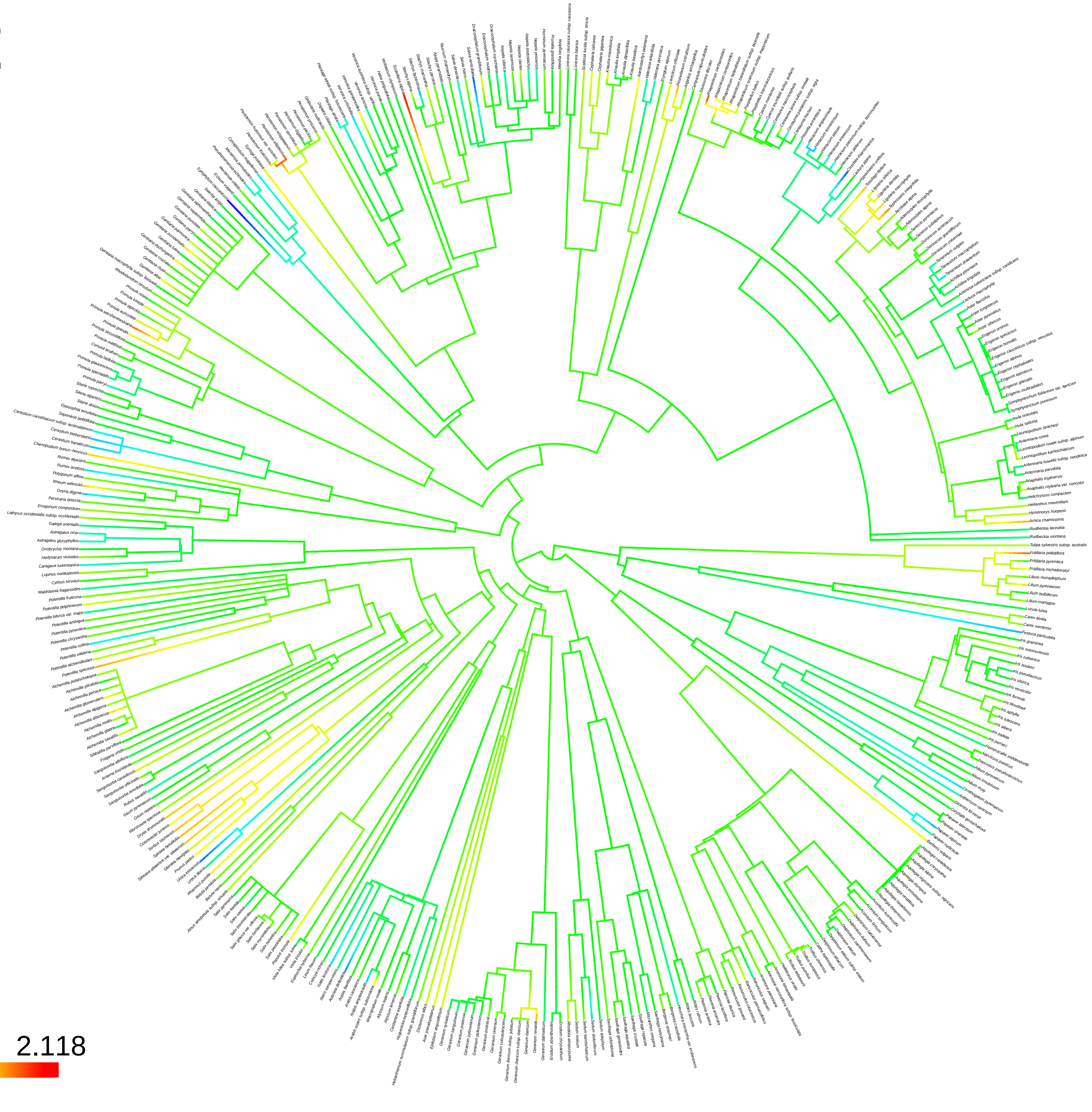

Figure 2
panel C

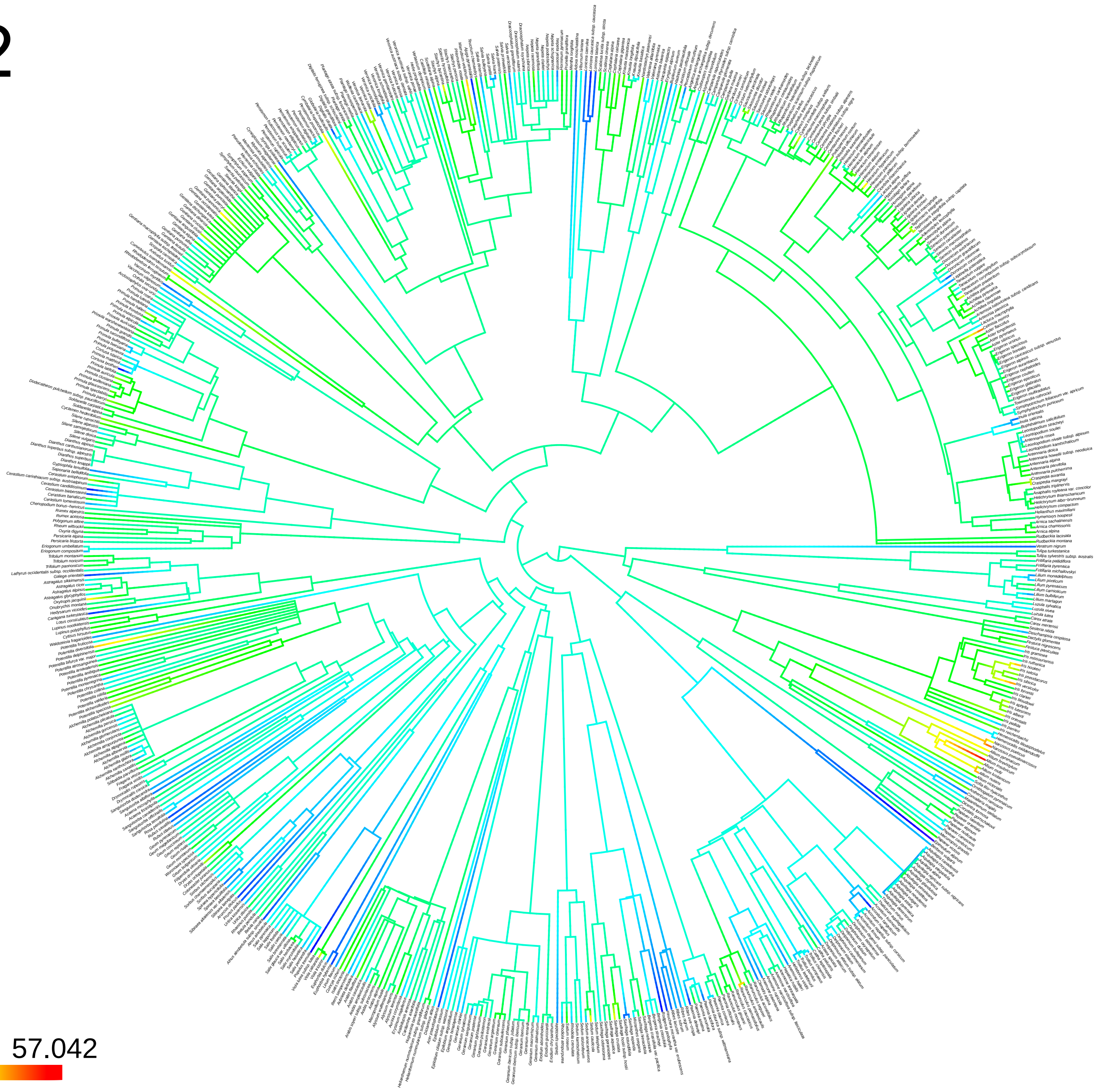

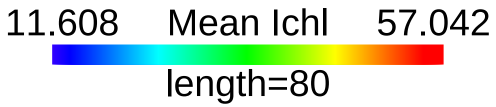

Figure 2 panel D

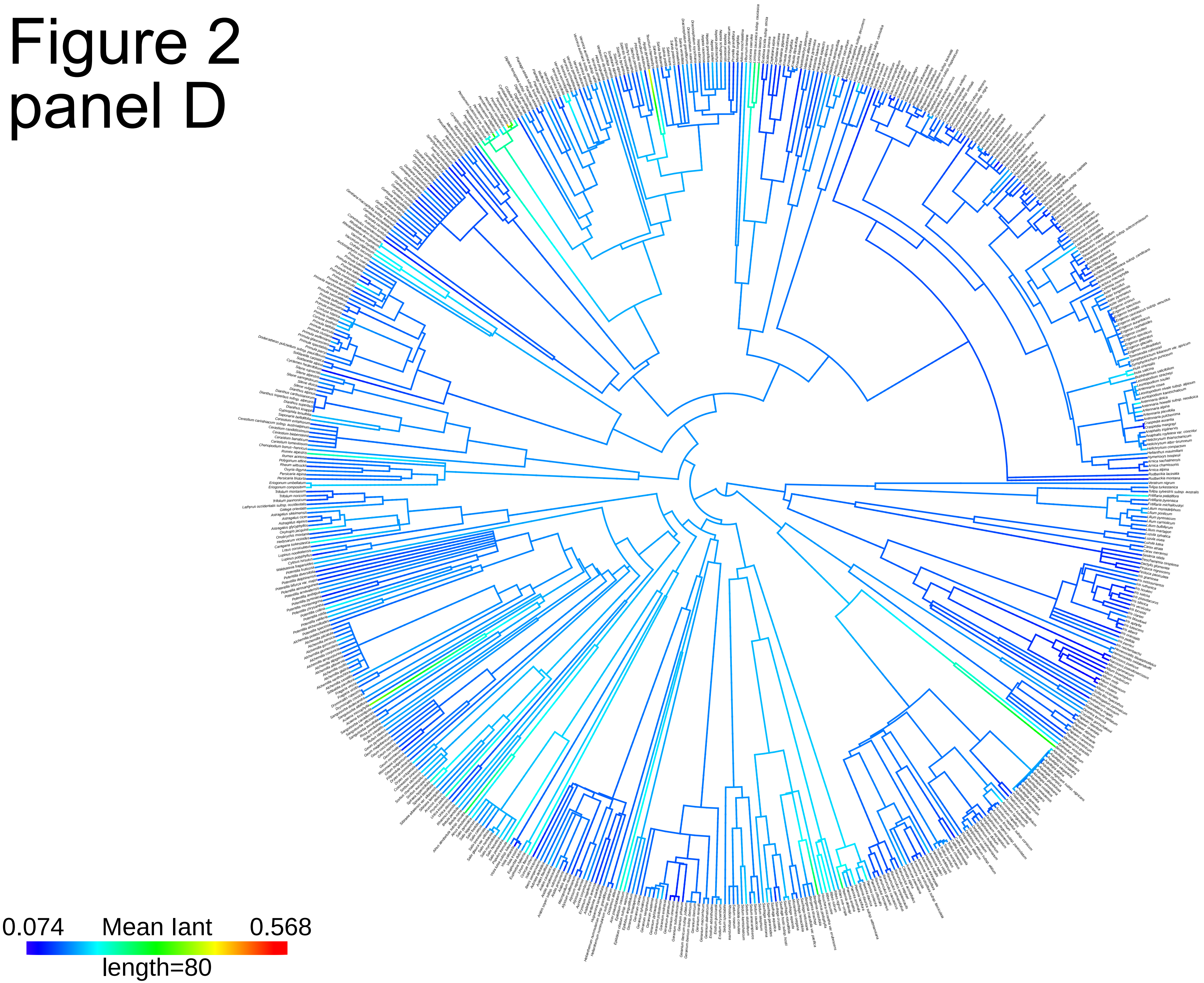

Supplement: Supplementary file 1 [file DataSheet_1.pdf]
