# Supplementary material for: The roles of species’ relatedness and climate of origin in determining optical leaf traits over a large set of taxa growing at high elevation and high latitude
Source: Front Plant Sci. 2022 Dec 16;13:1058162. doi: 10.3389/fpls.2022.1058162 (PMC9800846; doi:10.3389/fpls.2022.1058162)
Supplement: Supplementary file 2 [file DataSheet_2.pdf]

<u>Supplementary Material Appendix 2</u>: Quantifying micro-environmental variation in spectral irradiance, and the relationship between spectral irradiance and mean flavonol/flavone index (I_{flav})

MATERIALS AND METHODS

Solar spectral irradiance between the UV-B and near-infrared regions (290-900 nm) was measured with CCD array spectroradiometer Maya 2000 Pro (Ocean Optics, Dunedin, FL, USA) with D7-H-SMA cosine diffuser (Bentham Instruments Ltd., Reading, UK) from 29 differing locations within the alpine botanical garden. Measurements were done in 2015 during clear-sky weather and these data were used to assess and account for the micro-environmental variation in spectral irradiance throughout the garden. An equivalent set of spectroradiometer data, collected near Kumpula Botanical Garden (Helsinki, Finland) from the Viikki campus at the University of Helsinki (17.06.2015) were added to those made in the alpine botanical garden. We then aimed to crudely estimate the relationship between solar spectral irradiance and plant response for optically measured mean flavonol/flavone index (I_{flav}, arbitrary unit) in our study. The spectroradiometer measurement field protocol may be found in Hartikainen *et al*. (2018, 2020), and technical details related to calibration and stray light corrections may be found in Ylianttila *et al*. (2005), Aphalo *et al*., (2016) and Aphalo (2015, 2017). We used unweighted photon irradiance over wavelength regions (UV-B: 280-315 nm, UV-A: 315-400 nm, photosynthetically active radiation: 400-700 nm, blue: 420-490 nm, green: 500-570 nm, red: 620-680 nm, far-red: 720-750 nm, and near-infra-red: 750-900 nm), their photon ratios (blue:green, blue:red, R:FR: for Smith, 1982; 655-665 nm: 725-735 nm, and from Sellaro *et al*., 2010; 650-670 nm: 720-740 nm) and photon ratios × 1000 (UV-B:UV-A, UV-B:PAR). Furthermore, we calculated the biologically effective UV doses according to different biological spectral weighting functions (BSWFs) (UV action spectra: GEN(G): Green *et al*., 1974 mathematical formulation of generalized plant action spectrum; GEN(T): Thimijan *et al*., 1978 mathematical formulation of generalized plant action spectrum; PG: UV action spectrum for plant growth by Flint & Caldwell, 2003; DNA(N): UV action spectrum for damage to naked DNA by Setlow, 1974; CIE: action spectrum for erythema induced on human skin by McKinlay & Diffey, 1987; FLAV: UV action spectrum for accumulation of flavonol by Ibdah *et al*., 2002).

Solar spectral irradiance measured from an "open" environment, sunlight transmitted through the plant canopy resulting in shade, or deep shade, were associated with mean I_{flav} calculated from plants growing adjacent to spectroradiometer measurement location (mean number of measurements = 140, range of 7-749), according to their categorised light condition from a scale of one (i.e. mostly shaded over the day) to four (i.e. fully exposed). Solar spectral irradiance measured in an open area (Viikki, Helsinki), and only plants categorised as growing in sun-exposed light environments (number of measurements = 220) were used from Kumpula Botanical Garden. Spearman's rank correlation was then used to explore the relationship between solar spectral irradiance and mean I_{flav} of plants growing adjacent to spectroradiometer measurement locations.

RESULTS

We investigated whether there were differences in the relationship of mean I_{flav} and unweighted spectral irradiance, or effective UV doses calculated according to BSWFs, obtained from spectroradiometer measurements made within the alpine botanical garden in 29 locations, and at one location from Kumpula Botanical Garden. For unweighted UV regions, as well as for biologically effective UV doses calculated using BSWFs, solar spectral irradiance was significantly positively correlated ($r = 0.44$-0.52, $p < 0.05$) with mean I_{flav}, with little difference in the result irrespective of whether unweighted UV radiation or the BSWFs were used (A2 Table S1). On the contrary, this relationship was not significant for PAR regions nor for the spectral photon ratios we calculated (A2 Table S1). There was a large gap between the medium and the highest solar irradiance within PAR regions that caused the 95% CIs for loess-based fit (R function loess) to be very large (A2 Figure S1). While for UV regions, and especially the UV-B region, the large gap between values was less evident or absent in the latter case (A2 Figure S1). The unweighted UV-B irradiance measured in the open in Finland was similar to those measured in canopy shade in France (A2 Figure S1). On the contrary, PAR measured from Finland was among the highest, even in comparison to measurements in France (A2 Figure S1). These results were reflected in BSWFs, where those BSWFs in which shorter UV wavelengths have more weight (e.g. GEN(G), DNA(N)) gave lower effective UV doses for open Finnish location compared to open environments in France (A2 Figure S1).

Compared with those efforts previously made to produce comparable dose-response curves for different plant traits (Poorter *et al*., 2010, Esteban *et al*., 2014), our results only give crude estimates of the response of I_{flav} since solar irradiance received by plants was combined with mean I_{flav} calculated from many plants, and a relatively low number of observations were made ($n = 30$). Hence, these values should be treated as indicative of the I_{flav} response of plants to UV radiation. Furthermore, using mean values of traits narrowed the range of the data, and gave quite high values of I_{flav} (1.33-1.79 AU). Nevertheless, our results were reasonably well aligned with increase in I_{flav} accompanying the increase in effective UV doses calculated according to BSWF for flavonol (mesembryanthin) accumulation (FLAV action spectrum by Ibdah *et al*., 2002), which was made using a succulent plant *Mesembryanthemum crystallinum* as a study species.

A2 Table S1. Relationship between optically measured I_{flav} (arbitrary unit) and solar photon irradiance over different spectral regions and biological spectral weighting functions (BSWF) measured in 29 locations within the alpine botanical garden (Col du Lautaret, France) and one location in Helsinki, Finland equivalent to Kumpula Botanical Garden conditions in 2015. The 95 % confidence intervals (CI) for Spearman's rank correlation were computed by bootstrapping (R function spearman.ci from R package RVAideMemoire).

Wavelength region/BSWF	I_{flav}, *r* (95% CI)	Sig. level†
PPFD	0.40 (0.05, 0.65)	NS
UV-B irradiance, µmol m^{-2} s^{-1}	0.47 (0.17, 0.67)	*
UV-A irradiance, µmol m^{-2} s^{-1}	0.45 (0.15, 0.69)	*
Blue, µmol m^{-2} s^{-1}	0.42 (0.08, 0.68)	NS
Green, µmol m^{-2} s^{-1}	0.38 (0.02, 0.66)	NS
Red, µmol m^{-2} s^{-1}	0.39 (0.04, 0.67)	NS
Far-red, µmol m^{-2} s^{-1}	0.34 (-0.06, 0.64)	NS
GEN(G), µmol m^{-2} s^{-1}	0.49 (0.20, 0.69)	*
GEN(T), µmol m^{-2} s^{-1}	0.50 (0.23, 0.73)	*
PG, µmol m^{-2} s^{-1}	0.44 (0.14, 0.67)	*
DNA(N), µmol m^{-2} s^{-1}	0.52 (0.20, 0.74)	*
CIE, µmol m^{-2} s^{-1}	0.50 (0.20, 0.72)	*
FLAV, µmol m^{-2} s^{-1}	0.46 (0.15, 0.66)	*
Infra-red, µmol m^{-2} s^{-1}	0.27 (-0.14, 0.60)	NS
UV-B:UV-A	0.03 (-0.37, 0.42)	NS
UV-B:PAR	-0.06 (-0.44, 0.34)	NS
R:FR (Sellaro)	0.40 (0.05, 0.69)	NS
R:FR (Smith10)	0.38 (0.00, 0.67)	NS
R:FR (Smith20)	0.39 (0.02, 0.67)	NS
Blue:Green	0.14 (-0.25, 0.55)	NS
Blue:Red	-0.14 (-0.50, 0.26)	NS

n = 30, †significance levels: * <0.05, **≤0.01, ***≤0.001, ****≤0.0001 and Benjamini & Hochberg (1995) method was used to adjust p- values. Photon ratios are calculated as blue:green, blue:red, R:FR for Smith, 1982; 655-665 nm: 725-735 nm, and for Sellaro *et al.*, 2010; 650-670 nm: 720-740 nm, or as × 1000 (UV-B:UV-A, UV-B:PAR). The effective UV doses are calculated according to biological spectral weighting functions (BSWFs) (action spectra: GEN(G): Green *et al.*, 1974; GEN(T): Thimijan *et al.*, 1978; PG: Flint & Caldwell, 2003; DNA(N): Setlow, 1974; CIE: McKinlay & Diffey, 1987; FLAV: Ibdah *et al.*, 2002).

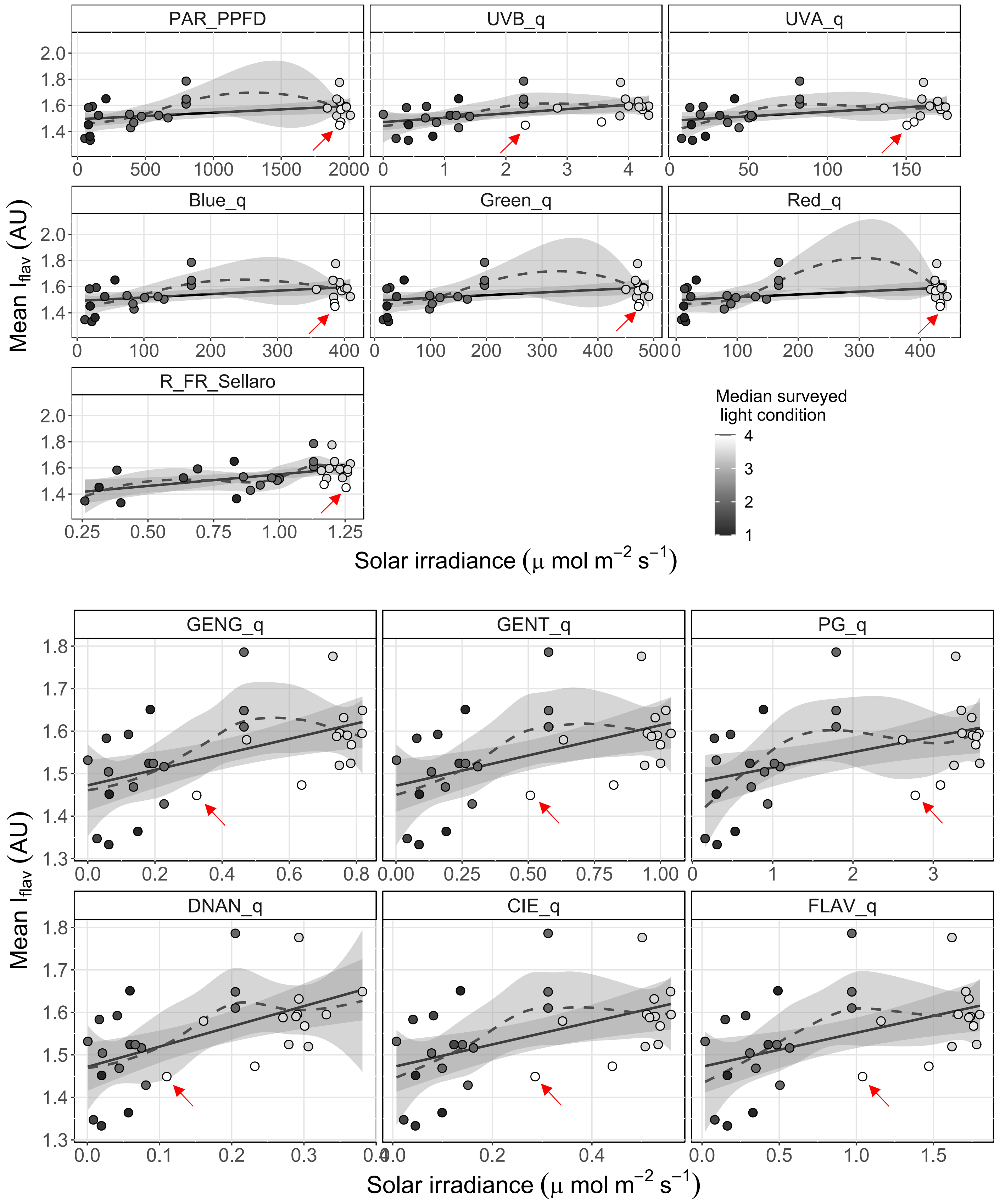

A2 Figure S1. The relationship between spectral photon irradiance and effective UV doses calculated according to BSWFs (action spectra: GEN(G), GEN(T), PG, DNA(N), CIE and FLAV) measured from 29 different locations within the alpine botanical garden and mean I_{flav} (Arbitrary Unit) of adjacent plants. One observation containing plants from an open environment in Kumpula Botanical Garden (Helsinki, Finland) was added by combining their mean I_{flav} with spectroradiometer measurements made around the same time (17.06.2015) from the nearby Viikki campus of the University of Helsinki (datapoint indicated with a red arrow). The colour scale of the points indicates the median of the categorised leaf light condition for the adjacent plants: where one (lowest) indicates "mostly shaded over day" in black, and four (highest), "fully exposed" in white. Plants growing adjacent to spectroradiometer measurement locations were associated with that measurement (i.e. from the open, light transmitted through plant canopy resulting in shade, or deep shade) according to their categorised light condition. The trend lines are given by a linear and loess-based fits (R function loess) to the cloud of points for each site with 95% CI (grey band).

REFERENCES

Aphalo, P. J. (2015). The r4photobiology suite. *UV4Plants Bull*, *1*, 21–29. https://doi.org/10.19232/uv4pb.2015.1.14.

Aphalo, P. J., Robson, T. M., & Piiparinen, J. (2016). How to check an array spectrometer [Online]. Available: http://uv4plants.org/methods/how-to-checkan-array-spectrometer/

Aphalo, P. J. (2017). Quantification of UV Radiation. In Jordan B. R. (Ed.), *UV-B Radiation and Plant Life: Molecular Biology to Ecology*. Oxford: UK: CABI International. https://doi.org/10.1079/9781780648590.0010

Benjamini, Y., & Hochberg, Y. (1995). Controlling the False Discovery Rate: A Practical and Powerful Approach to Multiple Testing. *Journal of the Royal Statistical Society: Series B (Methodological)*, *57*(1), 289–300. https://doi.org/10.1111/j.2517-6161.1995.tb02031.x

Esteban, R., Barrutia, O., Artetxe, U., Fernández-Marín, B., Hernández, A., & García-Plazaola, J. I. (2014). Internal and external factors affecting photosynthetic pigment composition in plants: A meta-analytical approach. *New Phytologist*, *206*(1), 268–280. https://doi.org/10.1111/nph.13186

Flint, S. D., & Caldwell, M. M. (2003). A biological spectral weighting function for ozone depletion research with higher plants. *Physiologia Plantarum*, *117*(1), 137–144. https://doi.org/10.1034/j.1399-3054.2003.1170117.x

Green, A. E. S., Sawada, T., & Shettle, E. P. (1974). The middle ultraviolet reaching the ground. *Photochemistry and Photobiology*, *19*(4), 251–259. https://doi.org/10.1111/j.1751-1097.1974.tb06508.x

Hartikainen, Saara M., Jach, A., Grané, A., & Robson, T. M. (2018). Assessing scale-wise similarity of curves with a thick pen: As illustrated through comparisons of spectral irradiance. *Ecology and Evolution*, *8*(20), 10206–10218. https://doi.org/10.1002/ece3.4496

Hartikainen, Saara Maria, Pieristè, M., Lassila, J., & Robson, T. M. (2020). Seasonal Patterns in Spectral Irradiance and Leaf UV-A Absorbance Under Forest Canopies. *Frontiers in Plant Science*, *10*, 1762. https://doi.org/10.3389/fpls.2019.01762

Ibdah, M., Krins, A., Seidlitz, H. K., Heller, W., Strack, D., & Vogt, T. (2002). Spectral dependence of flavonol and betacyanin accumulation in *Mesembryanthemum crystallinum* under enhanced ultraviolet radiation: UV response of *Mesembryanthemum crystallinum*. *Plant, Cell & Environment*, *25*(9), 1145–1154. https://doi.org/10.1046/j.1365-3040.2002.00895.x

McKinlay, A. F., & Diffey, B. L. (1987). A reference action spectrum for ultraviolet induced erythema in human skin. *CIE Journal 6*, 17–22.

Poorter, H., Niinemets, Ü., Walter, A., Fiorani, F., & Schurr, U. (2010). A method to construct dose–response curves for a wide range of environmental factors and plant traits by means of a meta-analysis of phenotypic data. *Journal of Experimental Botany*, *61*(8), 2043–2055. https://doi.org/10.1093/jxb/erp358

Sellaro, R., Crepy, M., Trupkin, S. A., Karayekov, E., Buchovsky, A. S., Rossi, C., & Casal, J. J. (2010). Cryptochrome as a Sensor of the Blue/Green Ratio of Natural Radiation in Arabidopsis. *Plant Physiology*, *154*(1), 401–409. https://doi.org/10.1104/pp.110.160820

Setlow, R. B. (1974). The Wavelengths in Sunlight Effective in Producing Skin Cancer: A Theoretical Analysis. *Proceedings of the National Academy of Sciences*, *71*(9), 3363–3366. https://doi.org/10.1073/pnas.71.9.3363

Smith, H. (1982). Light Quality, Photoperception, and Plant Strategy. *Annual Review of Plant Physiology*, *33*(1), 481–518. https://doi.org/10.1146/annurev.pp.33.060182.002405

Thimijan, R. W., Carns, H. R., & Campbell, L. E. (1978). *Final Report (EPA-IAG-D6-0168): Radiation sources and related environmental control for biological and climatic effects UV research (BACER)*. Tech. rep. Washington, DC: Environmental Protection Agency.

Ylianttila, L., Visuri, R., Huurto, L., & Jokela, K. (2005). Evaluation of a Single-monochromator Diode Array Spectroradiometer for Sunbed UV-radiation Measurements. *Photochemistry and Photobiology*, *81*(2), 333–341. https://doi.org/10.1111/j.1751-1097.2005.tb00192.x
