# Supplementary material for: The roles of species’ relatedness and climate of origin in determining optical leaf traits over a large set of taxa growing at high elevation and high latitude
Source: Front Plant Sci. 2022 Dec 16;13:1058162. doi: 10.3389/fpls.2022.1058162 (PMC9800846; doi:10.3389/fpls.2022.1058162)
Supplement: Supplementary file 3 [file DataSheet_3.pdf]

<u>Supplementary Material Appendix 3</u>: Comparing the relationship between optically measured flavonol/flavone index (I_{flav}) and absorbance of leaf extracts measured with spectrophotometer

INTRODUCTION

Many well-established methods can provide precise information on leaf flavonoids, such as quantities of specific compounds (Julkunen-Tiitto *et al*., 2015). However, these techniques are also invasive prohibiting undisturbed repeated *in vivo* sampling. Large scale repetitive sampling may be done using an optical leaf-clip method such as with Dualex Scientific + (henceforth Dualex) (Goulas *et al*., 2004; Cerovic *et al*., 2012). Some studies suggest that this technique may be species-specific (Lefebvre *et al*., 2016), and requiring along-side calibration. However, providing data over large variety of species is imperative for better utilisation of these techniques. We compared the optical measurements of I_{flav} (arbitrary units) with the absorbance of whole-leaf extracts measured with a spectrophotometer from 49 taxa growing in the alpine botanical garden (Col du Lautaret, France). In addition to these comparisons between optically measured I_{flav} and absorbance of leaf extracts within differing UV wavelengths (305/375 nm and mean UV-B/UV-A/UV region), we wanted to compare how the sampled taxa would vary in the shape of their UV absorbance spectra. Hence we applied a thick pen transform (TPT) and compared the UV spectra among taxa with thick pen measure of association (TPMA) (Fryzlewicz and Oh, 2011). We also compared the distribution of optically measured leaf traits and absorbance of leaf extracts of these taxa to their phylogenetic relatedness.

MATERIALS AND METHODS

A subset of 49 taxa measured in 2015 were selected to test the relationship between I_{flav} and leaf extracts. Leaves from four plants per taxa were collected at solar noon (± 3 h), and placed in cold and dark conditions in zip-lock bags until extraction in methanol within 1 hour. Two Dualex measurements were taken from the distal part of the adaxial leaf lamina avoiding any major veins. Two leaf-disks (2 × 0.28 cm^2 area) from the same area of the leaf were then punched into 3 ml of methanol (99.9 % MeOH) acidified with HCl 1:200. The samples were kept cool and dark overnight, and absorbance was measured the following day at 305 and 375 nm with spectrophotometer (Beckman DU series 64 UV-VIS, Brea, California, USA) using a quartz cuvette. The sample volume was diluted to keep the absorbance values ≤ 2.7, and data were subsequently normalized to sample volume and leaf-disk fresh weight.

During sampling, also two extra leaf-disks were obtained from an adjacent area of the leaf, immediately weighed for fresh weight, and dried at +60 °C for 48 hours before reweighing for dry weight. After one week the dried samples were extracted in 3 ml of acidified methanol (for 24 h) and spectral absorbance measured with UV-VIS spectrophotometer (Shimadzu UV-2501 PC UV-VIS, Kyoto, Japan) from 200-900 nm using a quartz cuvette. To test the relationship between extract absorbance and Dualex I_{flav} values, five different measures of absorbance were compared: absorbance at 305 and 375 nm (*in situ* extracts from fresh leaves and extracts from dried leaf samples), as well as integrated areas in the UV-B region (290-315 nm), UV-A region (315-400 nm) and the whole UV (UV-B plus UV-A) region (extracts from dried samples only). The region below 290 nm was excluded for its high values. The

relationship between optically measured I_{flav} and absorbance of whole leaf extracts was compared by using Spearman's rank correlation.

Spectral absorbance data from leaf extracts (dried samples) within the UV region (UV-A plus UV-B) was compared to distinguish species with similarities in their spectral absorbance by extracting the species mean spectrum, normalizing the data to a narrower similar scale (0-1) according to maximum values, applying TPT and finally using TPMA to compare the similarities between species' spectra. All details related to using TPT and TPMA may be found from Fryzlewicz and Oh (2011), and applied examples from Hartikainen *et al.* (2018). We also tested for a phylogenetic signal among leaf traits and absorbance of leaf extracts at different wavelengths by using Pagel's lambda (λ) test (Pagel, 1999) from phytools R package (Revell, 2012 & 2013). We used V.PhyloMaker tool (Jin & Qian, 2019) to generate a phylogeny for the studied taxa, utilising a published mega-tree as a backbone (GBOTB.extended.tre, Jin & Qian 2019).

RESULTS

Consistency of I_{flav} and absorbance of leaf extracts

The strongest relationship between I_{flav} and the absorbance of leaf extracts was at 375 nm, for both fresh ($r = 0.42$, $p < 0.0001$) and dried ($r = 0.45$, $p < 0.0001$) samples (A3 Figure S1). The correlations between I_{flav} and absorbance integrated over the different UV regions were all weak but significant: UV-B ($r = 0.20$, $p = 0.01$), UV-A ($r = 0.33$, $p < 0.0001$) and whole UV region ($r = 0.29$, $p = 0.00012$), while the correlations with absorbance at 305 nm was weak but significant for dried samples ($r = 0.19$, $p = 0.013$) but non-significant for the fresh samples ($r = 0.13$, $p = 0.097$) (A3 Figure S1). The relationship between I_{flav} and absorbance of leaf extracts at 375 nm differed for monocotyledons and dicotyledons whereby this relationship was significant only in dicotyledons (A3 Figure S2 panel B). This relationship did not differ for groups with maximum absorbance either around 290 nm or 330 nm (A3 Figure S2 panel A). Furthermore, there was a weak positive relationship between I_{flav} and dry weight of the leaf-disks used in leaf extracts (A3 Figure S3). This was also the case between I_{chl} and dry weight of the leaf-disks ($r = 0.44$, $p < 0.0001$). The taxa-specific relationships between I_{flav} and leaf extracts absorbance at 375 nm were not significant, when fresh and dried samples were combined to achieve sufficient sample size (data not shown).

Comparison of UV absorbance spectra of leaf extracts

Based on wavelength of the maximum value, normalized UV spectra could be divided roughly into two groups, whereby the first group of 23 taxa had highest absorbance values within 290-294 nm, often without distinguishable peak around 330 nm (13 taxa) (A3 Figure S4). Of the remaining 10 taxa within this group, some showed a peak at ~330 nm and some > 330 nm, but higher absorbance around 290 nm affected their normalization and hence spectral shape (A3 Figure S4). Accordingly, TPMA showed that these UV spectra varied greatly, with particularly low cross-dependence around 330 nm (A3 Figure S4).

The second group (21 taxa + *Eryngium alpinum* seedlings separately) had the highest normalized absorbance within 326-336 nm, and TPMA for these spectra showed highest cross-dependence around 330 nm, increasing with used "pen" thickness (A3 Figure S4). TPMA increased also between 380-400 nm (A3 Figure S4). However, cross-dependence among both groups was generally low resulting in negative values (A3 Figure S4). In addition to these

groups two *Allium* species (*A. victorialis, A. nutans*) had highest absorbance around 350 nm, two taxa around 360 nm (*Lilium pyrenaicum*, *Primula auriculata*) and one taxon (*Iris pallida*) did not fit into any groups according to its normalized maximum UV absorbance (A3 Figure S6). In most cases tested, maximum mean absorbance within the UV region was similar (within 3 nm) for all species belonging to the same genus (i.e. for *Alchemilla, Allium*, *Anemone, Erigeron, Eryngium, Gentiana, Geranium, Geum*, *Potentilla* and *Ranunculus*) (data not shown). Despite the visual resemblance in their UV spectra, the TPMA showed many negative values, indicating dissimilarity of the spectra (A3 Figure S6). Finally, we found fitted λ values for Pagel's λ test to be intermediate ($0 < \lambda < 1$) and significant for mean UV-B region and individual wavelengths (305, 375 nm and wavelength of maximum absorbance) for this dataset (A3 Figure S5, A3 Table S1).

DISCUSSION AND CONCLUSIONS

We found an overall relationship between absorbance of leaf extracts at 375 nm and I_{flav} (A3 Figure S1). This relationship differed for monocotyledons compared to dicotyledons, whereby it was only significant for the latter (A3 Figure S2 panel B). Flavonols and flavones are commonly found among flowering plants (Tripp *et al*., 2018 and references therein), and it has sometimes been suggested that major absorbers at 375 nm are flavonols in dicotyledons and flavones in monocotyledons (Cerovic *et al*., 2002 & 2012) despite both sometimes being present (Tripp *et al*., 2018 and references therein). Two *Lilium* species had the highest I_{flav} among monocotyledons in this dataset, but had low absorbance of leaf extracts. This may have contributed to the difference in these results between monocotyledons and dicotyledons.

The taxa-specific relationship was not significant, and a low sample size (n = 8 per species when fresh and dry samples were combined) may have contributed to our results. Furthermore, some phenolic compounds might have been absent from the leaf extracts. For instance, previous studies have found cell wall-bound phenolic compounds to be more common in those *Vaccinium* species with long leaf lifespans (Semerdjieva *et al*., 2003). In our earlier study testing forest understorey species, we found some indication that the relationship between absorbance of leaf extracts within UV-B region and I_{flav} differed between plants measured in spring and in summer, especially for *Aegopodium podagraria* (Hartikainen *et al*., 2020). This might suggest a differing ratio between the compounds measured by Dualex and whole leaf extracts over spring or upon changing light conditions. Previously, some studies have suggested that these optically measured compounds mirrored in I_{flav} may only represent small fraction of the whole leaf phenolics e.g. of *Vitis vinifera* leaves followed diurnally (Csepregi *et al*., 2019). Despite these results, the overall relationship found between I_{flav} and absorbance of leaf extracts at 375 nm suggests that generally optically measured I_{flav} gave relatively consistent and interpretable results as a method to compare adaxial leaf flavonoids in this set-up. Although caution should be taken in the interpretation of comparisons between taxa.

Despite the low TPMA values, absorbance UV spectra of leaf extracts were in general consistent within species from each genus, with some exceptions of varying spectra among species found in *Iris, Lilium, Primula* and *Veronica* (A3 Figure S6). Based on normalized maximum absorbance, two major groups were found where those with a clear peak at 330 nm were more consistent. Despite the distinctively different spectra within these two groups (normalized maximum ~290 and ~330 nm), the relationship between I_{flav} and absorbance of leaf extracts at 375 nm did not seem to differ (A3 Figure S2 panel A). Interestingly, we did not find distinctive patterns in TPT or TPMA according to e.g. class

(monocotyledons/dicotyledons), with the exception of deviant spectra from *Allium* species (A3 Figure S6). Low TPMA which we found from comparisons of UV spectra, may have been affected by too high absorbance at some peak values, and normalization of the spectra is also dependent on the included wavelengths. Including larger spectral region and comparing the peak values thoroughly might have provided further insight into which phenolic groups were present. Previous research has revealed complex glycosylation patterns in flavonoids (e.g. in Schmidt *et al.*, 2010: *Brassica oleracea* var. *sabellica*), and distinguishing these types of patterns would require different approach what we used here.

Our phylogenetic analysis suggests that relatedness might be more important in explaining the values along the phylogeny in absorbance of leaf extracts compared to optically measured counterparts in this subset of taxa (A3 Table S1). It may be that absorbance of leaf extracts even at a particular wavelength better mirrors the qualitative differences in leaf phenolics between taxa, since the shape of the absorbance spectrum is associated with qualitative differences in leaf phenolics. In general absorbance of leaf extracts compared to I_{flav} were more clearly divided between genera with few exceptions. This might have contributed to the significant results from Pagel's λ test.

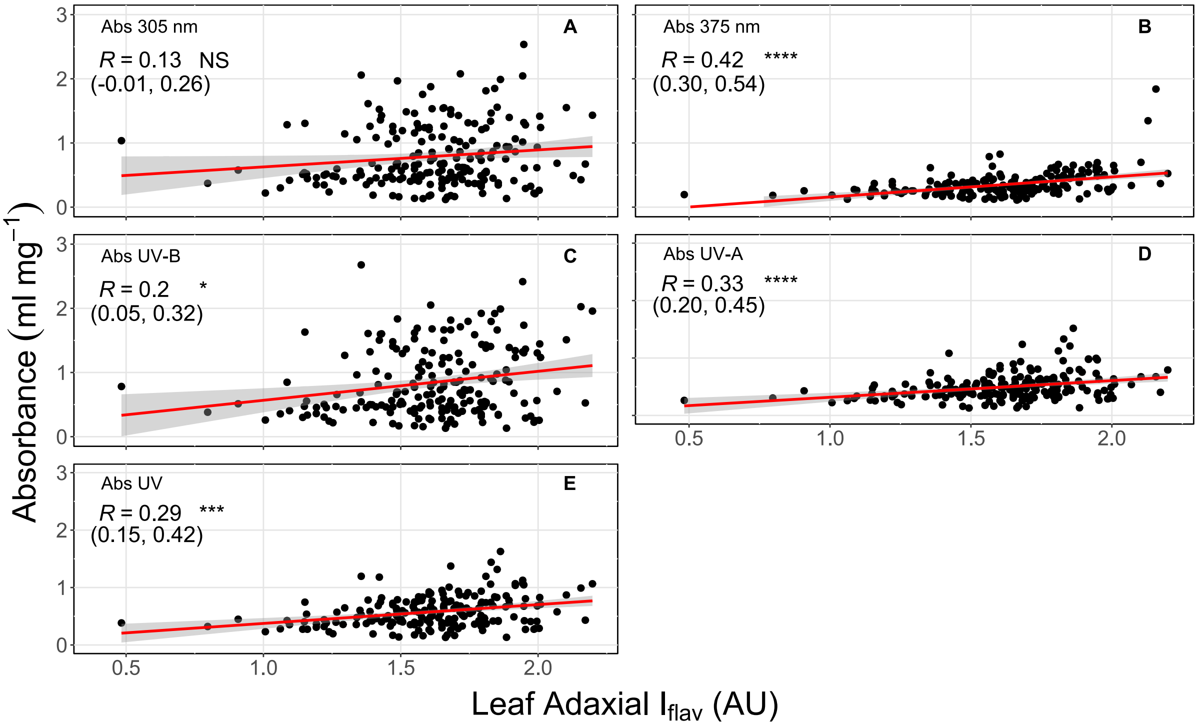

A3 Figure S1. Absorbance of leaf extracts (ml mg^{-1} of fresh weight) measured with the spectrophotometer compared to optically measured I_{flav} (Arbitrary Unit). Absorbance in panels **A** (at 305 nm) and **B** (at 375 nm) are measured from fresh samples, and in panels **C-E** (mean within UV-B, UV-A or entire UV region) are measured from dried samples. Spearman's rank correlation coefficient, significance (* <0.05, **≤0.01, ***≤0.001, ****≤0.0001) of adjusted *p*- values (Benjamini & Hochberg, 1995) and 95% confidence intervals by bootstrapping (R function spearman.ci from R package RVAideMemoire) are shown. A linear trendline is plotted to the points representing samples from 49 taxa with 95% confidence intervals (CI).

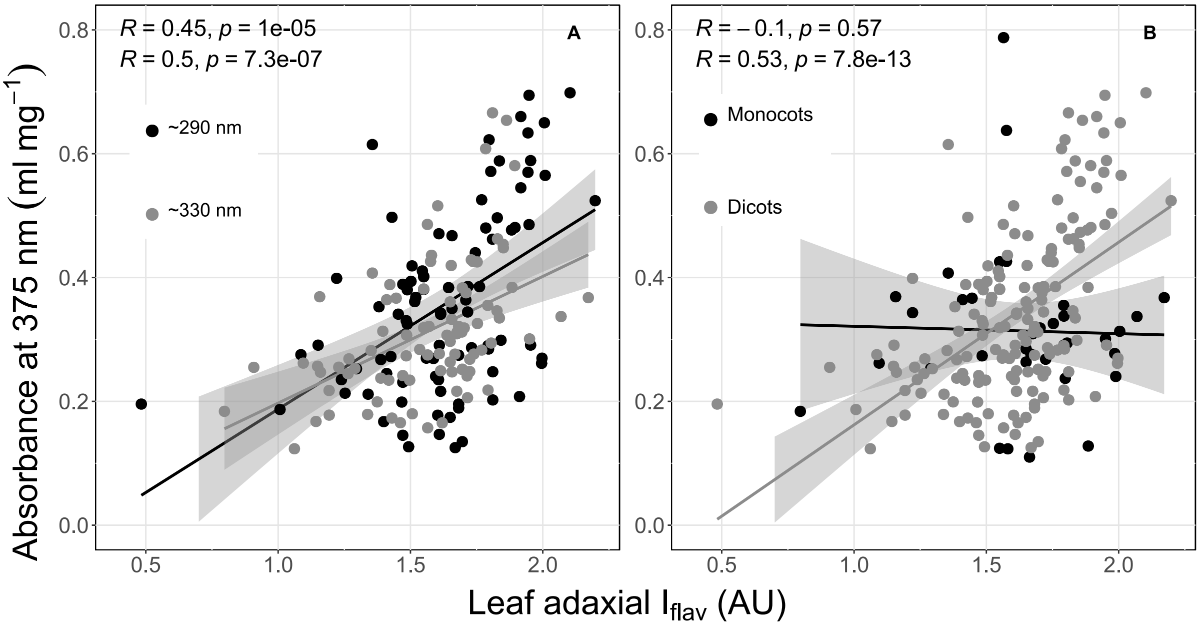

A3 Figure S2. Relationship between optically measured I_{flav} (Arbitrary Unit) and absorbance of whole leaf extracts at 375 nm. Panel **A** shows this relationship for those taxa with maximum normalized absorbance value around 290 nm (black) and around 330 nm (grey). Panel **B** shows the relationship for monocotyledons (black) and dicotyledons (grey). Respective Spearman's rank correlation and *p* -values are given in both panels (upper values are in **A**: ~290 nm, and **B**: monocotyledons). A linear trendline is plotted to the points with 95% CI. N.B. In panel **A** the CI is shown only for positive values, and there are less observations since five taxa had differing spectra (maximum absorbance outside ~290 and ~330 nm i.e. at ~ 350/360 nm or other wavelengths).

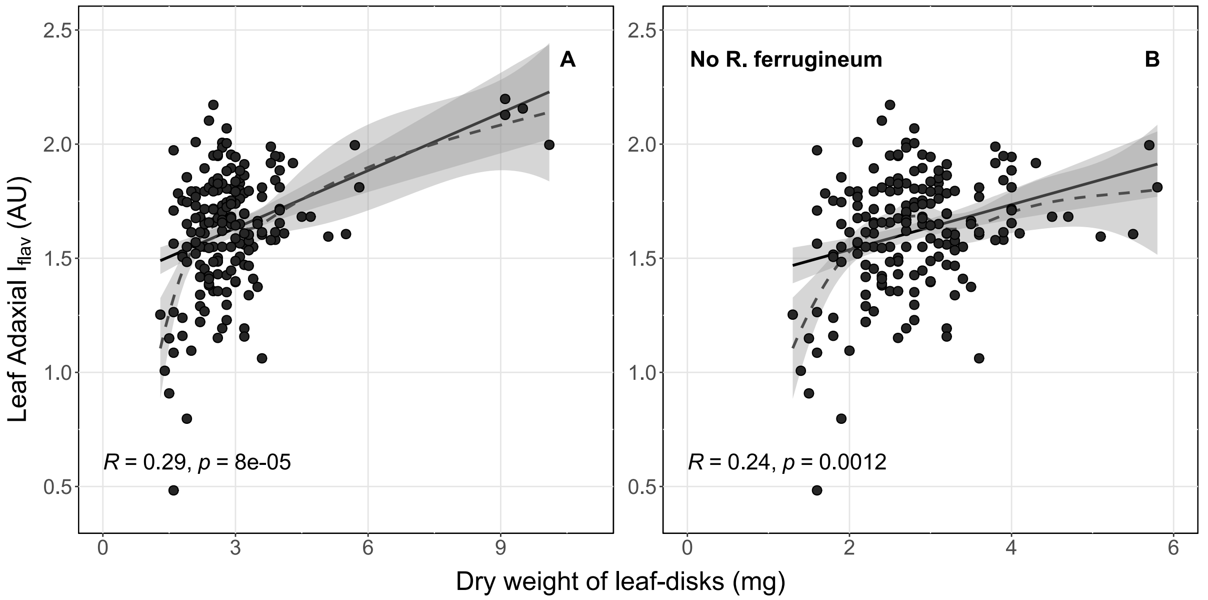

A3 Figure S3. Relationship between I_{flav} (Arbitrary Units) and summed dry weights of the two leaf-disks of 49 studied taxa (**A**). Spearman's rank correlation and respective *p*- value are given in each panel. In panel **B** data are plotted without *Rhododendron ferrugineum* (four points with highest dry weights in panel **A**)

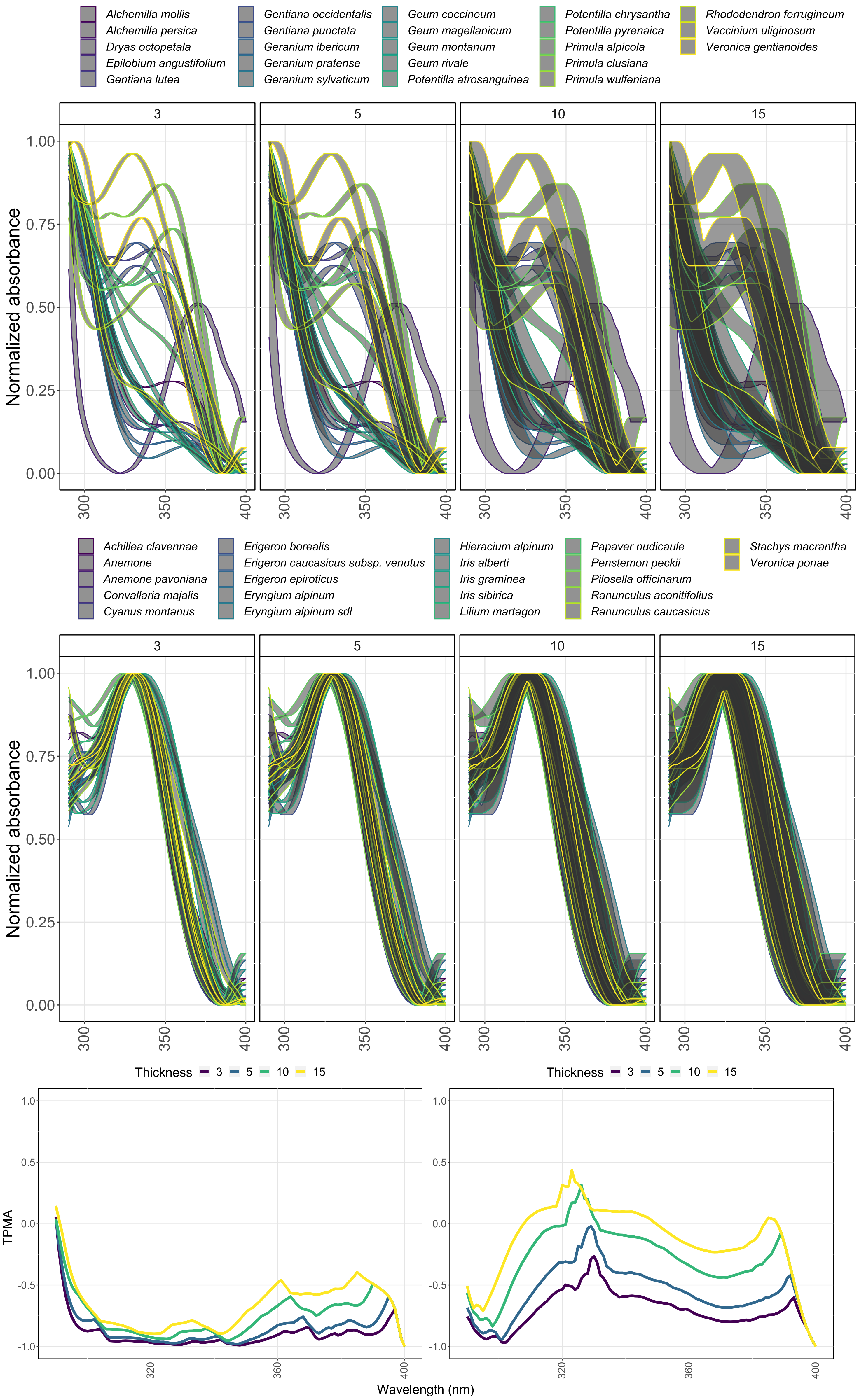

A3 Figure S4. Thick pen transform (TPT) made for normalized UV spectra measured from whole leaf extracts with a spectrophotometer, with panels indicating differing "pen" thicknesses used (above). First group's (23 taxa) highest normalized values were within 290-294 nm, while the other major group's (21 taxa) highest values were within 326-336 nm. Thick pen measure of association (TPMA) in the lower panels, shows low cross-dependence, especially within the spectra from groups of taxa with maximum absorbance ~290 nm (left). The TPMA made for the spectra from group with maximum absorbance ~330 nm is shown on the right. The TPMA shows the similarity of compared UV spectra (degree of cross-dependence) -the positive values indicate similarity and negative dissimilarity. Colours in the TPMA indicate the different thicknesses of pens used to calculate TPT. Five taxa not shown here could not be attributed to either group based on their normalized spectra (see results for details).

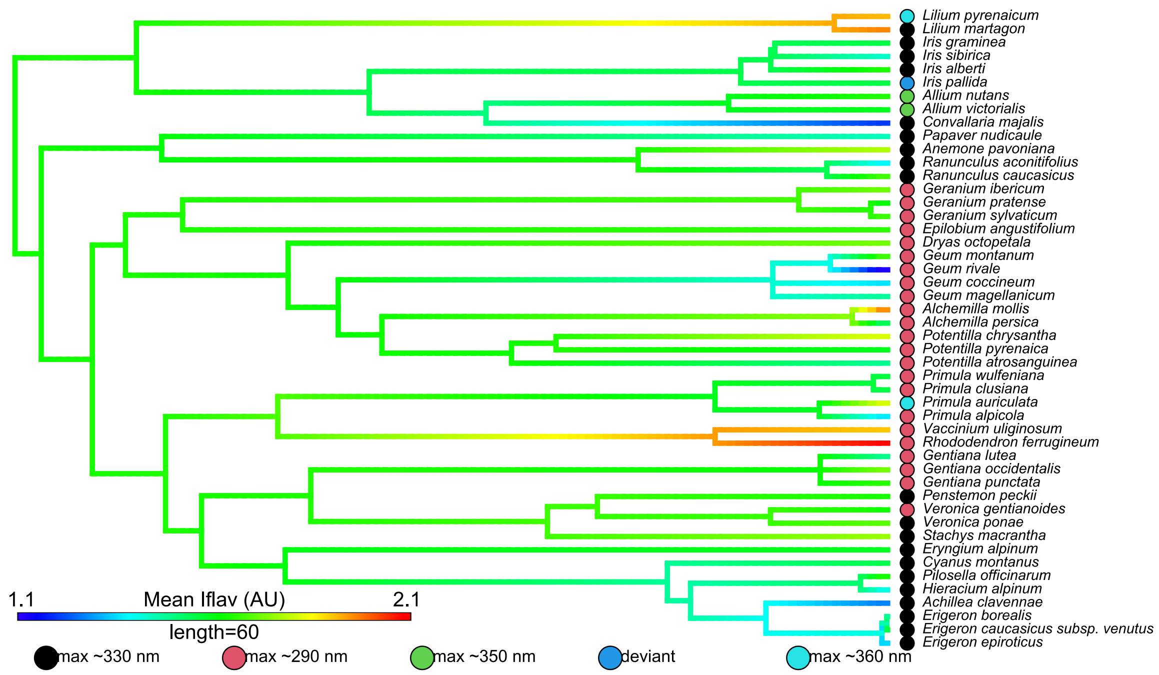

A3 Figure S5. Optically measured mean I_{flav} (Arbitrary Units) shown as trait values (colour scale) at the tips of the phylogenetic tree for the sampled 48 taxa (genus level measurements were left out) from the alpine botanical garden (Col du Lautaret, France). The grouping based on the maximum normalized absorbance values within the UV region (290-400 nm) of the leaf extracts is indicated as a coloured circle at the tip of each branch. A tool and methodology by Jin & Qian (2019, scenario 1) utilising an updated mega-tree (GBOTB.extended.tre) as a backbone was used to generate the phylogeny for the studied species. The visualisation was done by using R package phytools (Revell, 2012 & 2013).

A3 Table S1. Phylogenetic signal (Pagel's λ) calculated for a phylogeny based on Jin & Qian (2019) mega-tree, and optically measured mean leaf traits (I_{flav}, I_{chl}, and I_{ant}, all with arbitrary units) and mean absorbance of different UV regions (305/330/375 nm & UV-B/UV-A/whole UV region). Sampling was done in the alpine botanical garden (Col du Lautaret, France) during summer of 2015.

	Mean I_{flav}	Mean I_{chl}	Mean I_{ant}	Max wavelength within UV	Mean A 305 nm	Mean A 375 nm	Mean A 330 nm	Mean A UV-B region	Mean A UV-A region	Mean A UV region
Fitted value of λ	0.31	0.03	0.31	0.71	0.66	0.67	0.21	0.71	6.588E-05	0.35
Log-likelihood	8.28	-160.98	107.89	-207.44	-21.99	22.52	-25.29	-27.70	4.84	-2.76
Log-likelihood (λ = 0)	1.63	0.06	3.27	25.53	15.82	4.74	0.96	17.18	-2.410E-04	1.71
Significance†	NS	NS	NS	****	****	*	NS	****	NS	NS
Mean (± SE)	1.62 ± 0.019	31.93 ± 0.595	0.14 ± 0.002	313.69 ± 1.720	0.80 ± 0.035	0.35 ± 0.014	0.71 ± 0.031	0.86 ± 0.038	0.50 ± 0.017	0.58 ± 0.020
Median	1.65	30.41	0.14	321.00	0.65	0.31	0.59	0.71	0.46	0.54
Range (min-max)	0.48─2.20	15.17─54.97	0.09─0.25	290.0-365.0	0.12─2.54	0.11─1.84	0.15─2.63	0.13─2.68	0.13─1.52	0.13─1.63

†Significance levels: * <0.05, **≤0.01, ***≤0.001, ****≤0.0001

A3 Figure S6. Thick pen transform (TPT, on the left) and thick pen measure of association (TPMA, on the right) for all genera studied which included multiple species. Similarly, TPT and TPMA are also shown for monocotyledons and the group of taxa with maximum normalized absorbance around 360 nm. On the left, TPT for normalized absorbance UV spectra for each species sampled from the same genus with panels (3, 5, 10, 15) indicating the thickness of TPT used. On right, TPMA shows the similarity between UV spectra of species from the same genus, where positive values (degree of cross-dependence) indicate similarity and negative dissimilarity. Colours indicate TPMA calculated for TPT spectra of different thicknesses.

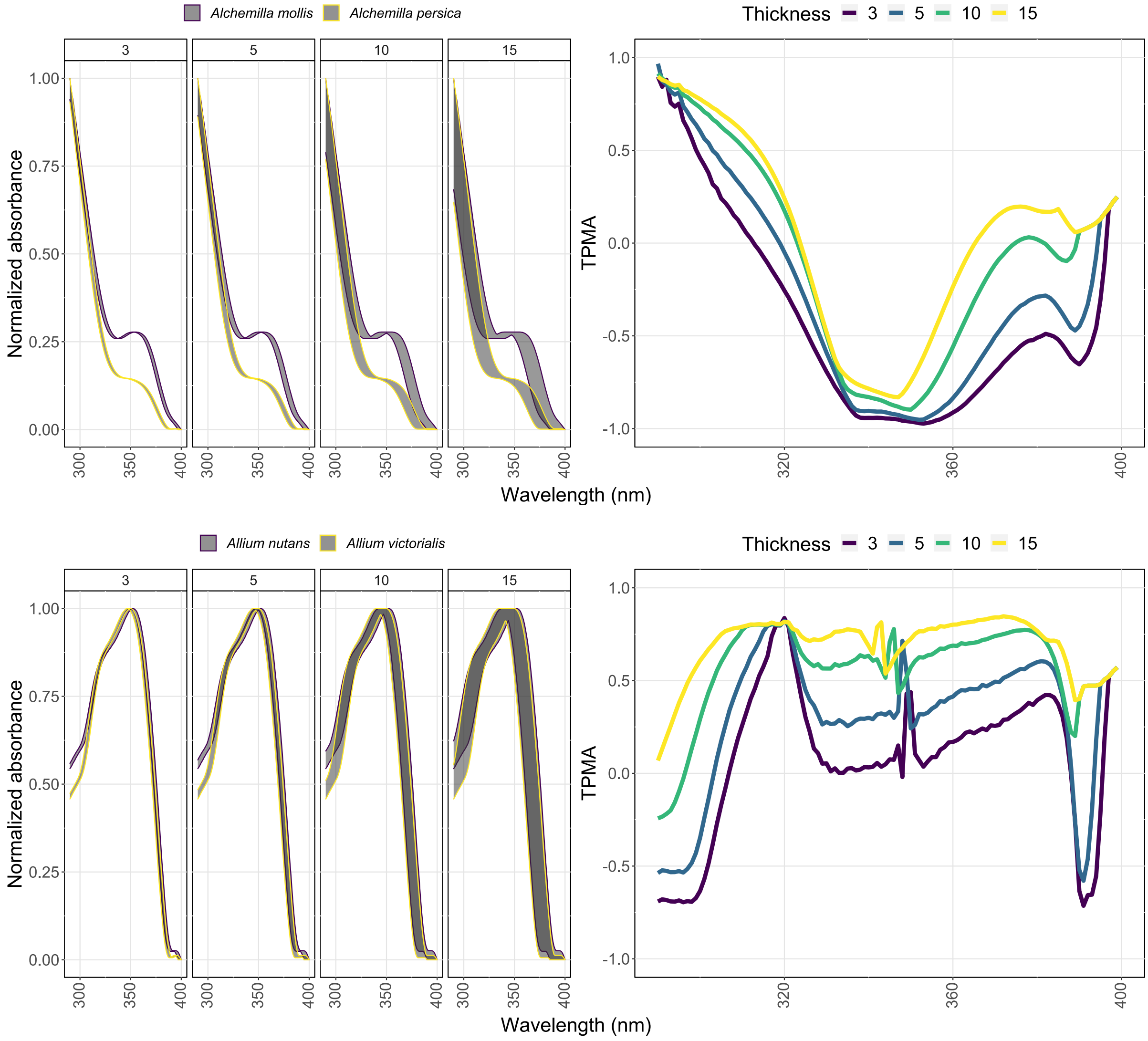

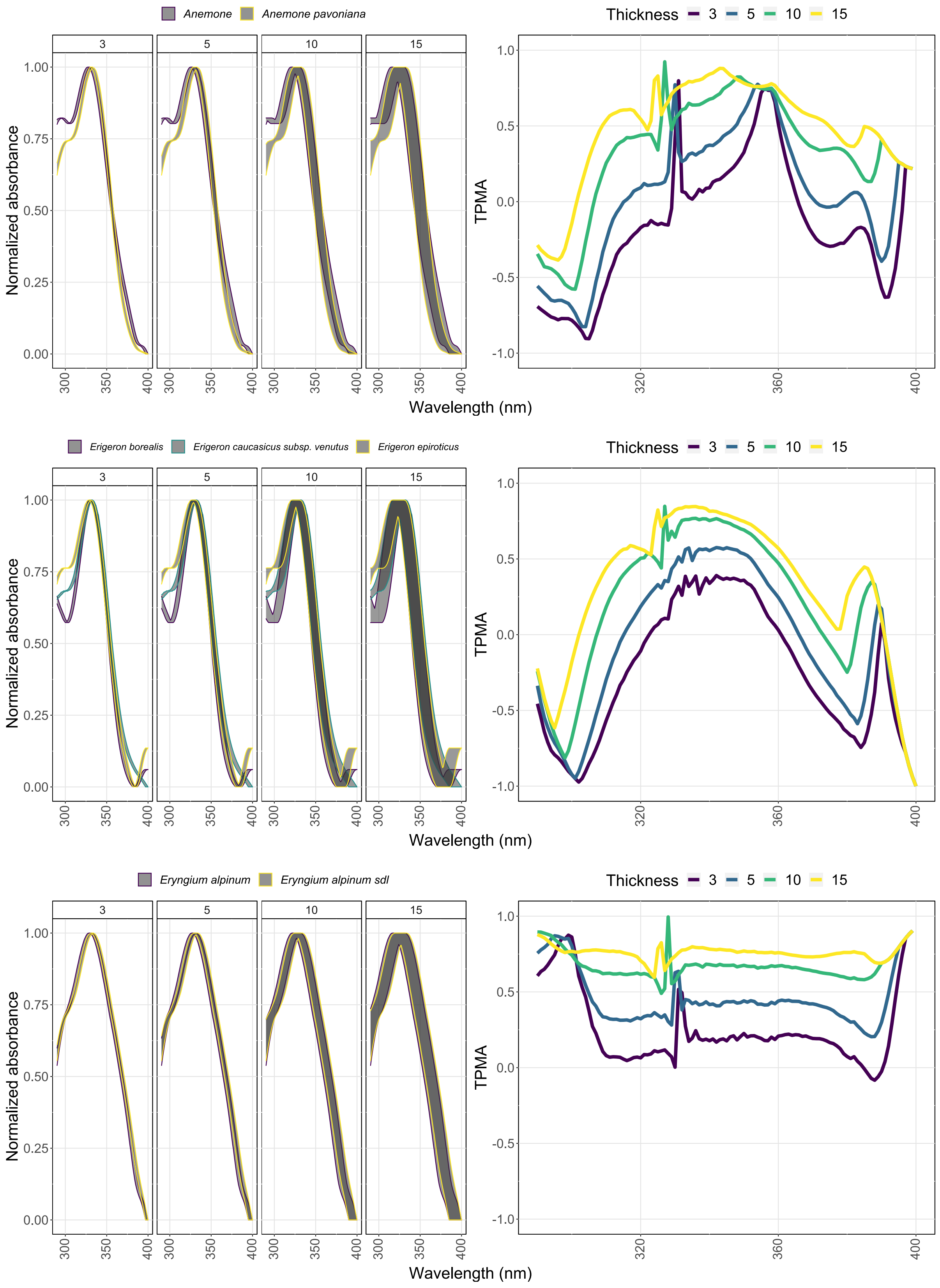
Anemone
Anemone pavoniana
Thickness 3 5 10 15
3
5
10
15
Normalized absorbance
TPMA
Wavelength (nm)
Erigeron borealis
Erigeron caucasicus subsp. venutus
Erigeron epiroticus
Thickness 3 5 10 15
Normalized absorbance
TPMA
Wavelength (nm)
Eryngium alpinum
Eryngium alpinum sdl
Thickness 3 5 10 15
Normalized absorbance
TPMA
Wavelength (nm)

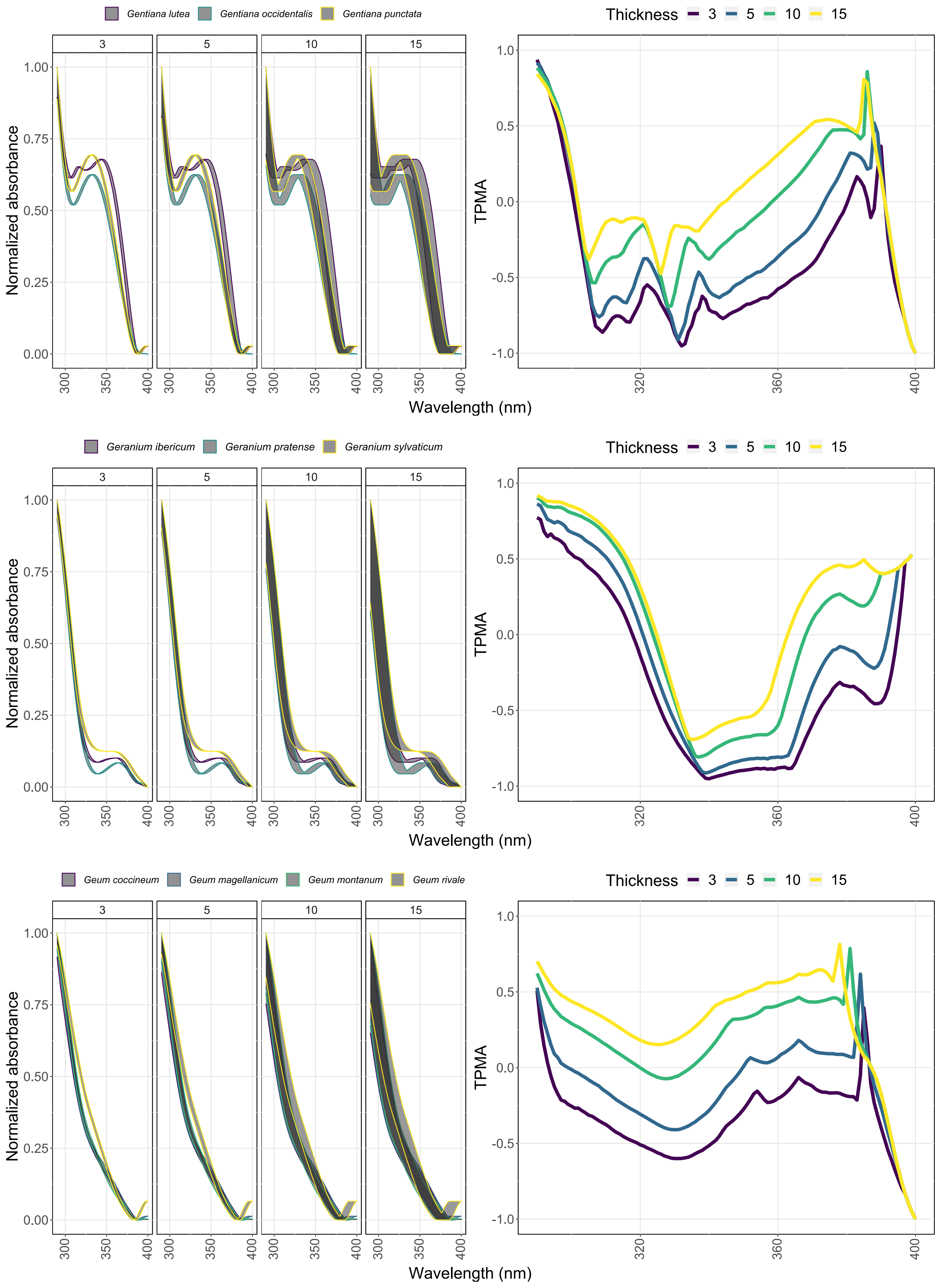

Gentiana lutea
Gentiana occidentalis
Gentiana punctata
Thickness 3 5 10 15
3
5
10
15
Normalized absorbance
TPMA
Wavelength (nm)
Geranium ibericum
Geranium pratense
Geranium sylvaticum
Geum coccineum
Geum magellanicum
Geum montanum
Geum rivale

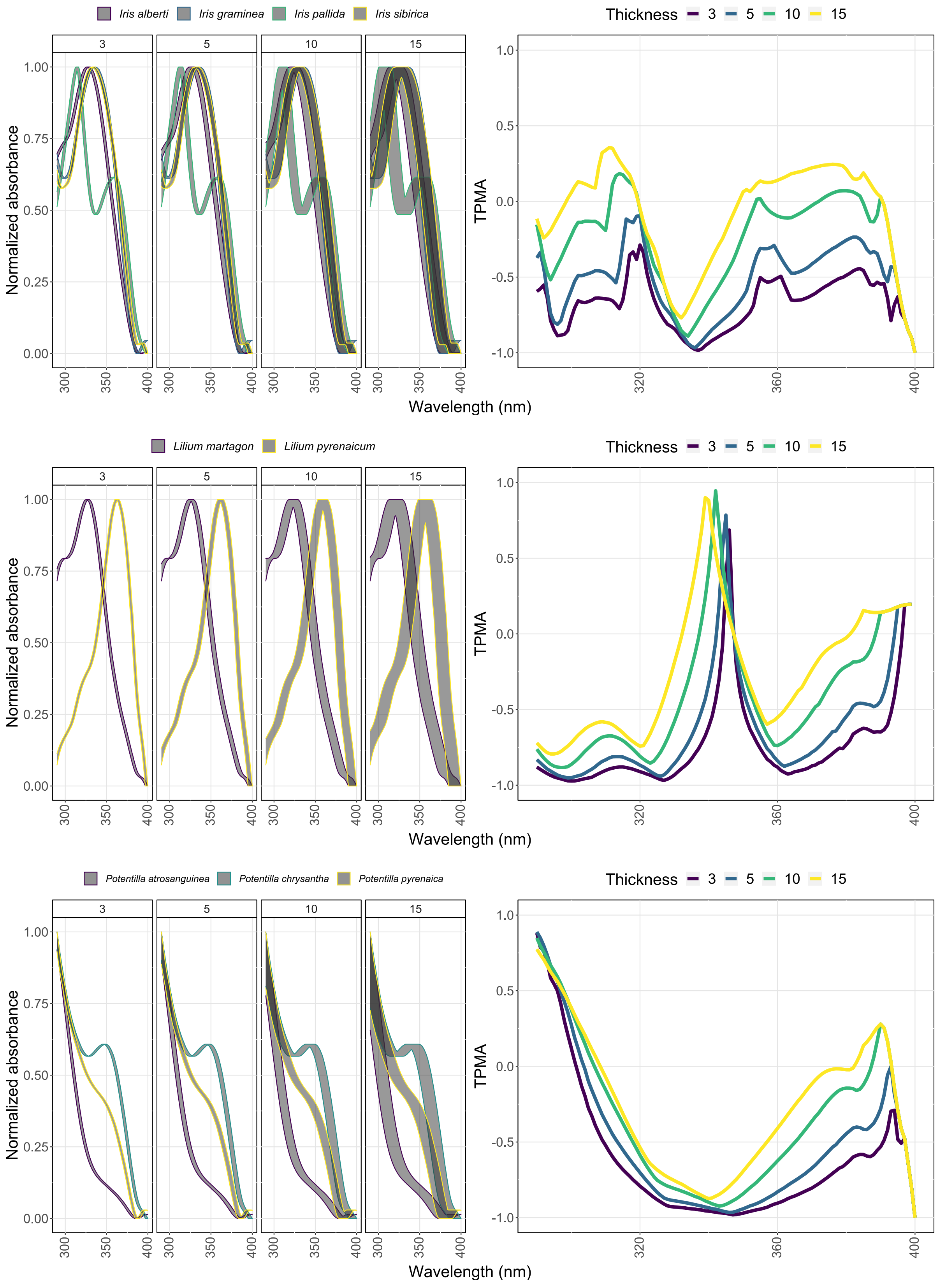

Iris alberti
Iris graminea
Iris pallida
Iris sibirica
Thickness
3
5
10
15
Normalized absorbance
TPMA
Wavelength (nm)
Lilium martagon
Lilium pyrenaicum
Potentilla atrosanguinea
Potentilla chrysantha
Potentilla pyrenaica

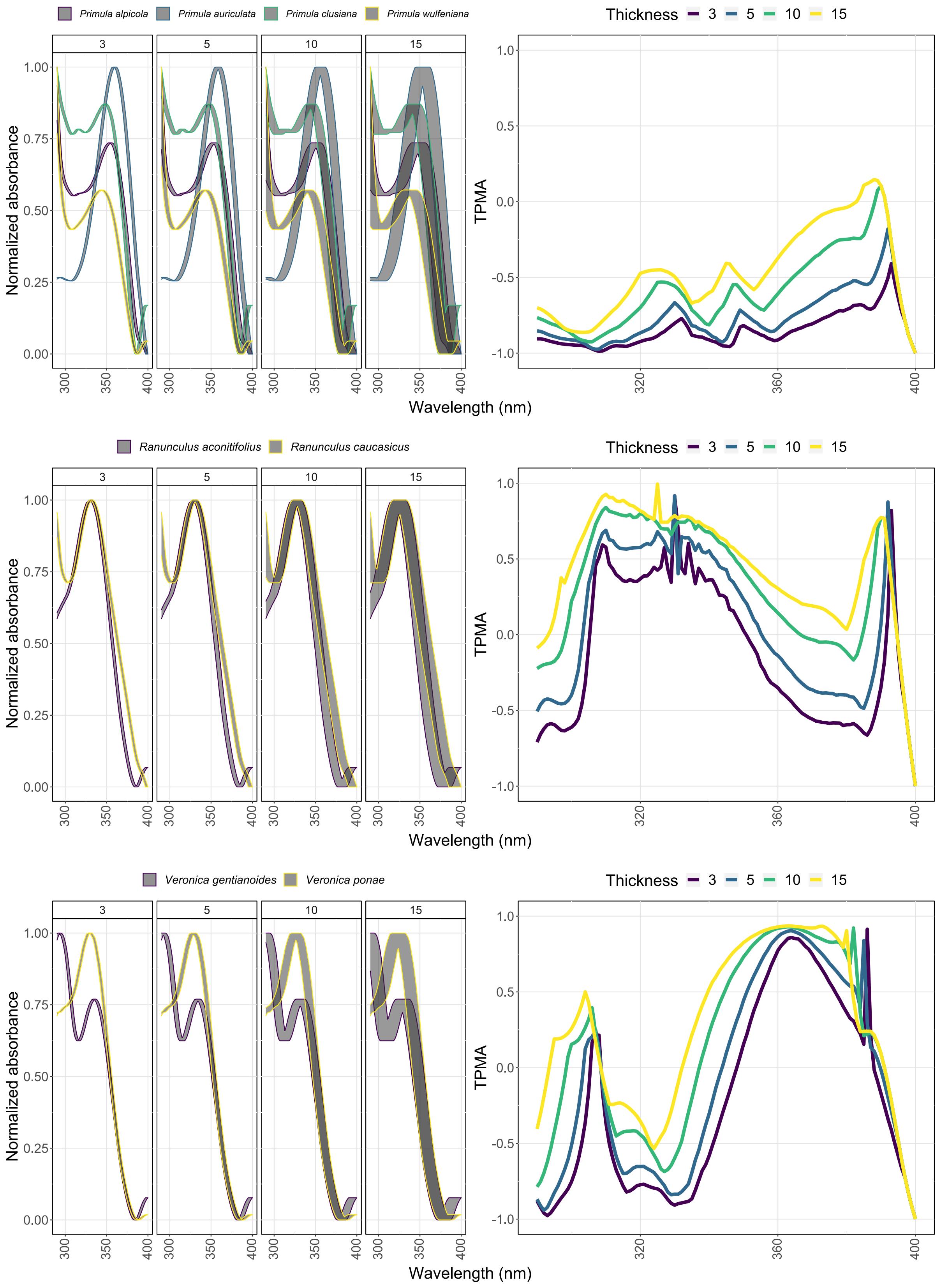

Primula alpicola
Primula auriculata
Primula clusiana
Primula wulfeniana
Thickness 3 5 10 15
3
5
10
15
Normalized absorbance
0.00
0.25
0.50
0.75
1.00
300
350
400
TPMA
-1.0
-0.5
0.0
0.5
1.0
320
360
400
Wavelength (nm)
Ranunculus aconitifolius
Ranunculus caucasicus
Veronica gentianoides
Veronica ponae

Monocotyledons:

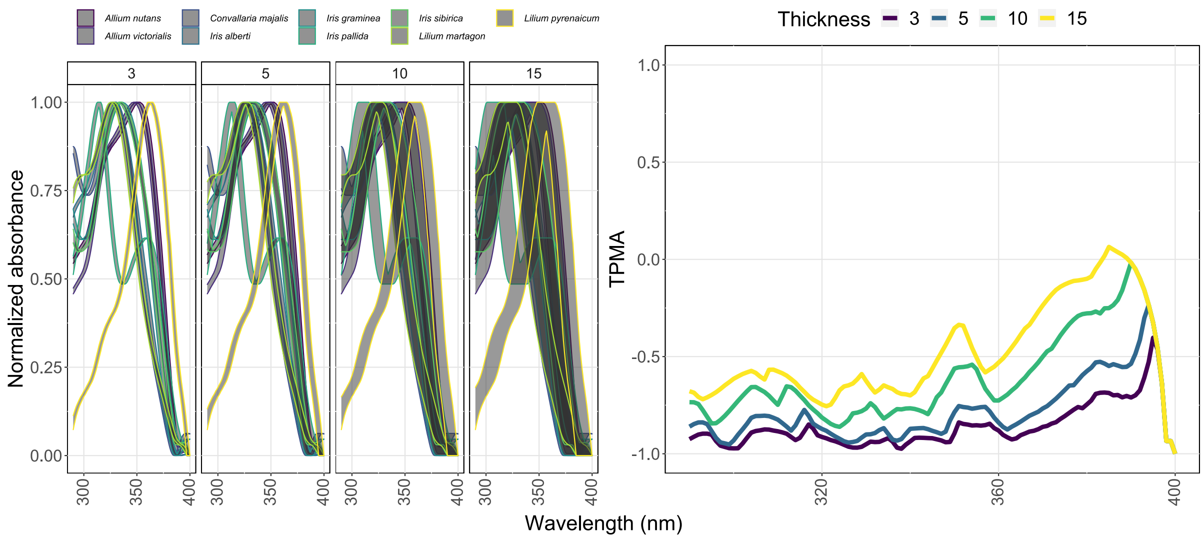

Group of maximum absorbance around 360 nm:

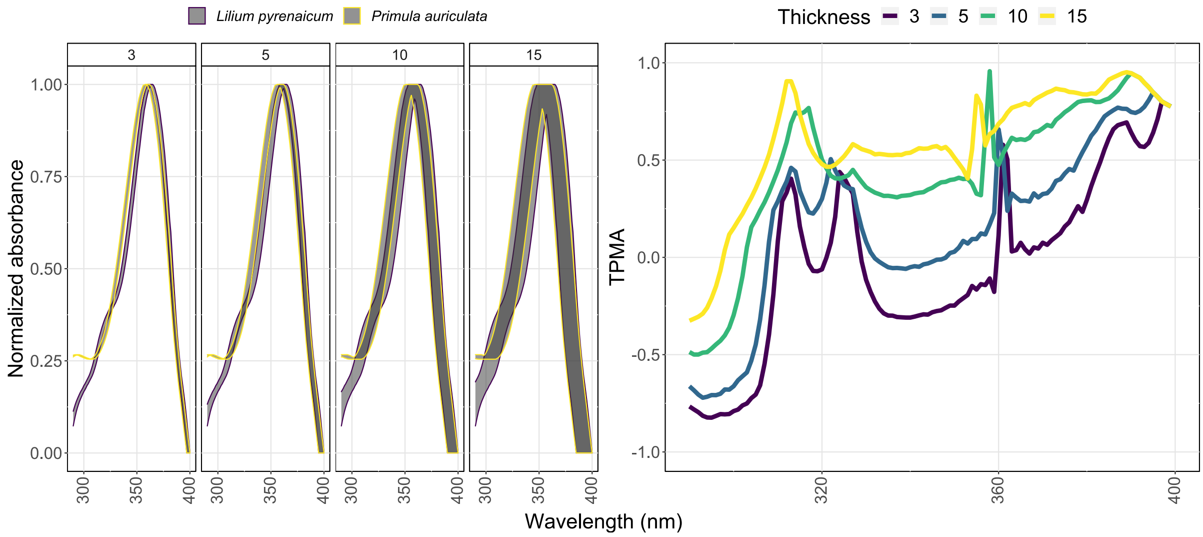

REFERENCES

Cerovic, Z. G., Ounis, A., Cartelat, A., Latouche, G., Goulas, Y., Meyer, S., & Moya, I. (2002). The use of chlorophyll fluorescence excitation spectra for the non-destructive in situ assessment of UV-absorbing compounds in leaves: UV-absorption spectra estimated from fluorescence. *Plant, Cell & Environment*, *25*(12), 1663–1676. https://doi.org/10.1046/j.1365-3040.2002.00942.x

Cerovic, Zoran G., Masdoumier, G., Ghozlen, N. B., & Latouche, G. (2012). A new optical leaf-clip meter for simultaneous non-destructive assessment of leaf chlorophyll and epidermal flavonoids. *Physiologia Plantarum*, *146*(3), 251–260. https://doi.org/10.1111/j.1399-3054.2012.01639.x

Csepregi, K., Teszlák, P., Kőrösi, L., & Hideg, É. (2019). Changes in grapevine leaf phenolic profiles during the day are temperature rather than irradiance driven. *Plant Physiology and Biochemistry*, *137*, 169–178. https://doi.org/10.1016/j.plaphy.2019.02.012

Fryzlewicz, P., & Oh, H.-S. (2011). Thick pen transformation for time series: Thick Pen Transformation. *Journal of the Royal Statistical Society: Series B (Statistical Methodology), 73*(4), 499–529. https://doi.org/10.1111/j.1467-9868.2011.00773.x

Goulas, Y., Cerovic, Z. G., Cartelat, A., & Moya, I. (2004). Dualex: A new instrument for field measurements of epidermal ultraviolet absorbance by chlorophyll fluorescence. *Applied Optics*, *43*(23), 4488. https://doi.org/10.1364/AO.43.004488

Hartikainen, S. M., Pieristè, M., Lassila, J., & Robson, T. M. (2020). Seasonal Patterns in Spectral Irradiance and Leaf UV-A Absorbance Under Forest Canopies. *Frontiers in Plant Science*, *10*, 1762. https://doi.org/10.3389/fpls.2019.01762

Jin, Y., & Qian, H. (2019). V.PhyloMaker: An R package that can generate very large phylogenies for vascular plants. *Ecography*, *42*(8), 1353–1359. https://doi.org/10.1111/ecog.04434

Julkunen-Tiitto, R., Nenadis, N., Neugart, S., Robson, M., Agati, G., Vepsäläinen, J., Zipoli, G., Nybakken, L., Winkler, B., & Jansen, M. A. K. (2015). Assessing the response of plant flavonoids to UV radiation: An overview of appropriate techniques. *Phytochemistry Reviews*, *14*(2), 273–297. https://doi.org/10.1007/s11101-014-9362-4

Lefebvre, T., Millery-Vigues, A., & Gallet, C. (2016). Does leaf optical absorbance reflect the polyphenol content of alpine plants along an elevational gradient? *Alpine Botany*, *126*(2), 177–185. https://doi.org/10.1007/s00035-016-0167-5

Pagel, M. (1999). Inferring the historical patterns of biological evolution. *Nature*, *401*(6756), 877–884. https://doi.org/10.1038/44766

Revell, L. J. (2012). phytools: An R package for phylogenetic comparative biology (and other things): phytools: R package. *Methods in Ecology and Evolution*, *3*(2), 217–223. https://doi.org/10.1111/j.2041-210X.2011.00169.x

Revell, L. J. (2013). Two new graphical methods for mapping trait evolution on phylogenies. *Methods in Ecology and Evolution*, *4*(8), 754–759. https://doi.org/10.1111/2041-210X.12066

Schmidt, S., Zietz, M., Schreiner, M., Rohn, S., Kroh, L. W., & Krumbein, A. (2010). Identification of complex, naturally occurring flavonoid glycosides in kale (*Brassica oleracea* var. *sabellica*) by high-performance liquid chromatography diode-array detection/electrospray ionization multi-stage mass spectrometry: Identification of naturally occurring flavonoid glycosides in kale. *Rapid Communications in Mass Spectrometry*, *24*(14), 2009–2022. https://doi.org/10.1002/rcm.4605

Semerdjieva, S. I., Sheffield, E., Phoenix, G. K., Gwynn-Jones, D., Callaghan, T. V., & Johnson, G. N. (2003). Contrasting strategies for UV-B screening in sub-Arctic dwarf shrubs: UV-B screening in Arctic shrubs. *Plant, Cell & Environment*, *26*(6), 957–964. https://doi.org/10.1046/j.1365-3040.2003.01029.x

Tripp, E. A., Zhuang, Y., Schreiber, M., Stone, H., & Berardi, A. E. (2018). Evolutionary and ecological drivers of plant flavonoids across a large latitudinal gradient. *Molecular Phylogenetics and Evolution*, *128*, 147–161. https://doi.org/10.1016/j.ympev.2018.07.004
